# Synthesis, Characterization and Biological Profile of Cationic Cobalt Complexes with First-Generation Quinolones

**DOI:** 10.3390/molecules30122646

**Published:** 2025-06-19

**Authors:** Alexia Tialiou, Antonios G. Hatzidimitriou, George Psomas

**Affiliations:** Laboratory of Inorganic Chemistry, Department of Chemistry, Aristotle University of Thessaloniki, GR-54124 Thessaloniki, Greece

**Keywords:** first-generation quinolones, cobalt complexes, interaction with calf-thymus DNA, binding with albumins

## Abstract

The interaction of cobalt(II) with first-generation quinolones oxolinic acid (Hoxo), flumequine (Hflmq), pipemidic acid (Hppa) and cinoxacin (Hcx) in the presence of the *N*,*N′*-donor heterocyclic ligands 2,2′-bipyridine (bipy) or 1,10-phenanthroline (phen) afforded a series of novel cobalt complexes, namely [Co(bipy)_2_(oxo)](PF_6_)_2_·H_2_O (**1**), [Co(phen)_2_(oxo)](PF_6_)_2_·0.5CH_3_OH·0.5H_2_O (**2**), [Co(bipy)_2_(flmq)](PF_6_)_2_·0.5CH_3_OH·0.5H_2_O (**3**), [Co(bipy)_2_(ppa)](PF_6_)_2_·CH_3_OH·0.5H_2_O (**4**), [Co(phen)_2_(cx)](PF_6_)_2_·CH_3_OH·0.5H_2_O (**5**), and [Co(phen)_2_(flmq)](PF_6_)·0.5CH_3_OH·H_2_O (**6**). The characterization of the complexes involved physicochemical techniques, various spectroscopies and single-crystal X-ray crystallography. The affinity of complexes to calf-thymus (CT) DNA was monitored with various techniques, suggesting intercalation in-between the DNA-nucleobases as the most probable interaction mode, which may be combined with electrostatic interactions as a result of the cationic nature of the complexes. The affinity of the complexes for bovine and human serum albumin proteins was monitored, and the determined corresponding albumin-binding constants revealed a tight and reversible interaction.

## 1. Introduction

Quinolones (4-quinolones or quinolonecarboxylic acids, HQ) are commonly used antibacterial agents that are used for the treatment of infections [1,2]. Nalidixic acid was the first quinolone introduced in the treatment of urinary tract infections in 1963 [3]. Since then, a variety of synthesized quinolones that have undergone structural modifications have shown a wider spectrum of activity [1,2,4]. The main mode of their activity is the inhibition of DNA replication, which is mainly accomplished via the inhibition of the enzyme topoisomerase, which participates in this biological function [5,6].

**Figure 1:**
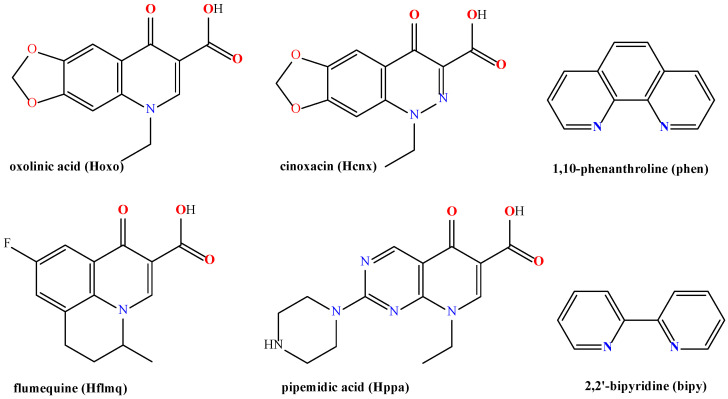
The syntax formula of the quinolones oxolinic acid (Hoxo), flumequine (Hflmq), cinoxacin (Hcnx), and pipemidic acid (Hppa) and the *N*,*N′*-donors 1,10-phenanthroline (phen), and 2,2′-bipyridine (bipy) used in the present study.

Depending on their activity spectrum, quinolones are classified into generations [7]. Besides nalidixic acid, the most common first-generation quinolones (Figure 1) are cinoxacin (Hcnx), flumequine (Hflmq), oxolinic acid (Hoxo) and pipemidic acid (Hppa), which are active against Gram-(−) microorganisms (except *Pseudomonas* species), and they are used only for the treatment of urinary tract infections [6,7,8]. The increasing interest in the synthesis and characterization of transition metal complexes with quinolone ligands is mainly due to the utilization of metal ions in the treatment of various diseases and the synergistic effect stemming from the coordination of the quinolones to metal ions, which may lead to improved pharmacological activity [9,10,11,12,13]. Within this context, a significant number of reports concerning metal (namely Cu(II), Ni(II), Mn(II), Co(II), Zn(II), Cd(II), Ru(II), and Er(III)) complexes of Hoxo [14,15,16], Hflmq [17,18,19,20,21,22,23], Hppa [24,25,26] and Hcnx [27,28,29,30,31,32,33,34] may be found in the literature, where the structural characterization and biological properties of these complexes are described.

The biological role of cobalt is well-established since it has been found in the active center of vitamin B12 (cobalamine), which participates in important biological functions related to DNA [35,36]. Since 1952, when the first reports concerning the toxicity of the optical isomers of [Co(en)_3_](NO_3_)_3_ (en = ethylenediamine) [37], a plethora of cobalt compounds showing biological activities have been found in the literature. More specifically, the cobalt complex Doxovir© (or CTC-96) has shown antiviral activity and completed successfully clinical trials phase II for the treatment of *Herpes simplex labialis* and viral eye infections [38,39], and the Co(II)-porphyrin is a typical potential agent for photodynamic therapy activated by X-ray excitation [40,41]. Furthermore, cobalt compounds are known for their antifungal [42,43], antimicrobial [44,45], antiparasitic [46], antirheumatic [47], antitubercular [48], antiviral [49] and cytotoxic [50,51] activity. Considering quinolones agents as ligands, a series of Co(II) complexes may be found in the literature (i.e., with ciprofloxacin [31], enoxacin [52], enrofloxacin [53], flumequine [18], oxolinic acid [14], norfloxacin, sarafloxacin [54], and sparfloxacin [55]).

Cobalt(III) forms substitutionally inert complexes, allowing for greater structural stability and more controlled biological interactions. This property is particularly advantageous when designing complexes intended for targeted or selective biological effects [56,57]. Co(III) complexes can act as prodrugs or enzyme inhibitors that remain inert under physiological conditions and become activated under specific stimuli such as reduction or light [58,59,60]. This concept is well supported in the literature, where Co(III) complexes with ligands such as 1,10-phenanthroline, 2,2’-bipyridine, or Schiff bases have shown equal or even superior antimicrobial and anticancer activity compared to their Co(II) analogs [56,58,61]. Moreover, polypyridyl ligands stabilize the Co(III) oxidation state effectively, making such complexes well-suited for in vitro and in vivo studies [62]. In hypoxia-targeted therapies, for example, Co(III) complexes serve as stable carriers that release active species only after bioreduction in tumor environments [60,63,64].

Motivated by the biological relevance of cobalt and the market share of quinolones (HQ), we have prepared and characterized a series of cobalt–quinolone complexes, each bearing two *N*,*N′*-donor co-ligands (namely, 2,2′-bipyridine (bipy) or 1,10-phenanthroline (phen), Figure 1). The choice of such *N*,*N′*-donors was based on the enhanced biological activity observed for metal complexes bearing bipy or phen as co-ligands resulting from their chelating binding [13,18,21,62]. The choice of Co(III) ion was based on its distinct coordination properties, potential for selective activation, and strong precedent in the literature demonstrating its bioactivity under appropriate conditions. For the synthesis of these compounds, the previously reported [65,66,67] complexes [Co(bipy)_2_Cl_2_]Cl (**a**) and [Co(phen)_2_Cl_2_](H_2_O) (**b**) were used as precursors. The interaction of compounds **a** and **b** with the quinolones Hoxo, Hflmq, Hppa and Hcx resulted in the isolation of six novel complexes of the general formula [Co(*N*,*N′*-donor)_2_(Q)](PF_6_)_x_, where x = 1 or 2. Five of the complexes, i.e., [Co(bipy)_2_(oxo)](PF_6_)_2_·H_2_O (**1**), [Co(phen)_2_(oxo)](PF_6_)_2_·0.5CH_3_OH·0.5H_2_O (**2**), [Co(bipy)_2_(flmq)](PF_6_)_2_·0.5CH_3_OH·0.5H_2_O (**3**), [Co(bipy)_2_(ppa)](PF_6_)_2_·CH_3_OH·H_2_O (**4**), and [Co(phen)_2_(cx)](PF_6_)_2_·CH_3_OH·0.5H_2_O (**5**) are dicationic cobalt(III) complexes (x = 2), while [Co(phen)_2_(flmq)](PF_6_)·0.5CH_3_OH·H_2_O (**6**) is a monocationic cobalt(II) complex (x = 1). The characterization of the complexes was accomplished by elemental analysis, IR, UV–vis and ^1^H NMR spectroscopies, and single-crystal X-ray crystallography.

To initially evaluate the biological relevance of the complexes, the interaction with calf–thymus (CT) DNA and albumins was examined. The mode and the strength (i.e., determination of the DNA-binding constants of the complexes (K_b_)) of the interaction of the complexes with CT DNA were assessed with UV–vis spectroscopy, DNA–viscosity measurements, cyclic voltammetry, and via competitive binding studies with the classic intercalator ethidium bromide (EB), which were studied with fluorescence emission spectroscopy. Furthermore, the interaction of the complexes with bovine serum albumin (BSA) and human serum albumin (HSA) was monitored with fluorescence emission spectroscopy to check their affinity and to determine the corresponding binding constants.

## 2. Results and Discussion

### 2.1. Synthesis and Spectroscopic Characterization

For the synthesis of complexes **1**–**6**, compounds [Co(bipy)_2_Cl_2_]Cl (**a**) and [Co(phen)_2_Cl_2_](H_2_O) (**b**) were prepared after adjusting the procedure described in the literature [65,67] and were used as precursor compounds. The structures of compounds (**a**) and (**b**) were verified by single-crystal X-ray crystallography. Equimolar quantities of the precursor compounds and the corresponding quinolone HQ were reacted in a polar solvent mixture MeOH/H_2_O (1:1) under aerobic conditions at a basic pH (~9) and refluxed at 60 °C overnight followed by the addition of the bulky anion PF_6_^−^ which led to the isolation on appreciable yields of colored single-crystals via slow evaporation at room temperature. In particular, the isolation of single-crystals of complexes **4** and **5** was time-consuming since it occurred after two to three months of slow evaporation; for this reason, the long time needed to provide an appropriate product was the reason for deterring the studies of these complexes in solution and the interaction with CT DNA and albumins.

Complexes **1**–**6** are stable in air and are soluble in polar solvents MeOH and DMSO and partially soluble in H_2_O. The compounds were characterized with elemental analysis, molar conductivity measurements, IR, UV-vis and ^1^H NMR (used only for the Co(III) complexes **1**–**5**) spectroscopies and single-crystal X-ray crystallography.

The results of elemental analysis combined with X-ray diffraction data may confirm the proposed formulas of the synthesized complexes. Molar conductivity measurements of the complexes were carried out in a DMSO solution (1 mM). According to the literature [68] concerning molar conductivity measurements in DMSO solutions, Λ_Μ_ values in the range ~50–70 mho·cm^2^·mol^−1^ are indicative of a 1:1 electrolyte, and Λ_Μ_ values in the range ~90–110 mho·cm^2^·mol^−1^ are usually derived for 1:2 electrolytes. Complexes **1**–**4** exhibiting Λ_Μ_ values in the range 105–125 mho·cm^2^·mol^−1^ are 1:2 electrolytes, while the Λ_Μ_ value of 60 mho·cm^2^·mol^−1^ found for complex **6** suggests that it is a 1:1 electrolyte. These results are in good agreement with the formulas of the complexes derived with X-ray crystallography.

The coordination of the functional groups of quinolones and the existence of i-donor co-ligands were studied initially using IR spectroscopy (Figure S1). Complexes **1**–**6** present two characteristic bands at the IR spectra of the carboxylate group of quinolone ligands; the first band located in the range 1638–1651 cm^−1^ can be attributed to the antisymmetric stretching vibration (ν_asym_(COO)) of the carboxylato group, and the second band in the region 1410–1452 cm^−1^ can be attributed to the symmetric stretching vibration (ν_sym_(COO)). A marker of the coordination mode of the carboxylato ligand is the value of the parameter Δν(COO) [= ν_asym_(COO) − ν_sym_(COO)] [69]. The Δν(COO) values of complexes **1**–**6** are in the range 191–238 cm^−1^, suggesting a monodentate coordination of the carboxylate group of quinolone ligands [70]. The coordination of quinolone ligands via the pyridone oxygen can be indicated by the slight shift of the stretching vibration ν(C=O)_pyr_ towards 1609–1628 cm^−1^. Thus, the as-expected bidentate coordination of the quinolone ligands via the pyridone oxygen and a carboxylate oxygen may be confirmed [14,15,18,20]. A characteristic band of the out-of-plane ρ(C-H) vibration of the *N*,*N′*-donor co-ligands bipy (ρ(C-H)_bipy_) and phen (ρ(C-H)_phen_) was observed at 769–773 cm^−1^ and at 717–718 cm^−1^, respectively [69]. In addition, the stretching vibration at 840–843 cm^−1^ may be assigned to the existence of the PF_6_^−^ counter anion. The proposed coordination of the ligands was also confirmed by single-crystal X-ray crystallography.

In the UV-vis spectra of the complexes, the band observed in the visible region at 496–514 nm (ε = 90–185 M^−1^cm^−1^) may be attributed to a d-d transition typical for octahedral cobalt complexes [71]. In addition, bands attributed to the intra-ligands transitions typical for the quinolone ligands were also observed in the UV region of the spectra. The ^1^H NMR spectra of the Co(III) complexes were recorded in 95:5 D_2_O:DMSO-d_6_ mixtures and showed all the expected resonance signals (Figure S2), which were shifted slightly in comparison with the spectra of the free quinolones [20,21,72]. Any signals attributed to dissociated ligands were not observed, proving the stability of the complexes in the DMSO solution [20,21,72]. In addition, the ^1^H NMR spectra were recorded both immediately after sample preparation and after 24 h, showing no detectable changes (Figure S3), which suggests that the complexes remain stable in solution over time.

### 2.2. Structures of the Complexes

All compounds prepared (**a**, **b** and **1**–**6**) were isolated as single-crystals suitable for the determination of the structure with single-crystal X-ray crystallography. The structures of the precursor compounds **a** and **b** are similar to those reported in the literature [65,66,67] verifying their formula.

**Figure 2:**
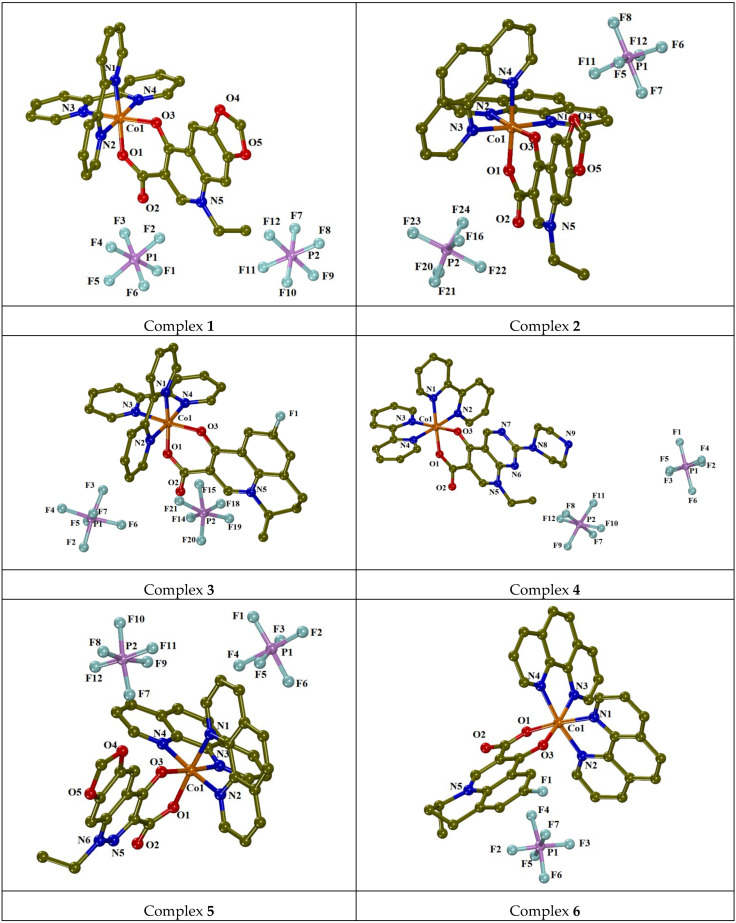
Molecular structures of complexes **1**–**6**. Hydrogen atoms, disordered atoms and solvate molecules are omitted for clarity. Carbon atoms are given in dark yellow.

Complexes **1** and **4** crystallized in the triclinic system and space group *P*-1 (Table S1), while complexes **2**, **3**, **5** and **6** crystallized in the monoclinic system and space groups *P*2_1_/*c* (for **2**, **3** and **6**) or *P*2_1_/*a* (for **5**). The crystal structures of mononuclear complexes **1**–**6** are displayed in Figure 2, and selected angles and bond lengths are summarized in Table 1 and Table S2. Compounds **1**–**5** are Co(III) complexes and **6** is a Co(II) complex.

Compounds **1**–**5** are dicationic complexes, and their +2 charge is neutralized by two PF_6_^−^ counter anions, while compound **6** is a monocationic complex, which is neutralized by a PF_6_^−^ counter anion. The deprotonated quinolone ligands are coordinated in a bidentate chelate way to the cobalt ion via the carboxylato oxygen O1 and the pyridone oxygen O3. The two *N*,*N′*-donor ligands (bipy or phen) are bound to cobalt ions via the nitrogen atoms N1, N2, N3 and N4 in a bidentate chelate fashion. In all these complexes, the Co1 ion is six-coordinated, bearing a CoN_4_O_2_ chromophore with a distorted octahedral geometry.

Considering the bond distances around the cobalt ions, the ones of Co(III) complexes (complexes **1**–**5**) are shorter than those around Co(II) (complex **6**) (Table 1). The Co1-O bond distances (=1.878(2)–1.9055(19) Å in complexes **1**–**5** and ~1.923(2) Å in complex **6**) are slightly shorter than the Co1-N bond distances (=1.904(3)–1.964(3) Å in complexes **1**–**5** and 2.018(2)–2.042(3) Å in complex **6**) (Table 1).

The solvate molecules participate in the formation of intermolecular hydrogen bonds in all complexes, thus contributing to further stabilization of the structures (Table S3).

### 2.3. Interaction of the Complexes with CT DNA

DNA displays different interaction modes with coordination compounds bearing drug ligands; their stability in nature, as well as the structure and nature of their ligands, may influence this interaction [73]. Such interactions can be observed along the double helix of DNA, and complexes may interact in a covalent or non-covalent fashion or by inducing the cleavage of the DNA helix [74,75]. The binding affinity of DNA with complexes bearing ligands with biological interest has been studied extensively. Titration studies with UV-vis spectroscopy, viscosity measurements, cyclic voltammetry and via EB-displacement ability monitored by fluorescence emission spectroscopy were employed to examine the affinity of complexes **1**–**3** and **6** for CT DNA.

UV-vis spectroscopy may provide useful information on the interaction of complexes with CT DNA, resulting in the calculation of the corresponding DNA-binding constant (K_b_). The foregoing study involves two steps; the first step evaluates the shift (λ_max_ = 258 nm) of the UV-band of CT DNA with an increasing amount of the studied compound, while the second step evaluates the changes induced to the intra-ligand bands of the complexes due to the incremental quantities of DNA. In all cases, the red shift may indicate the stabilization of the DNA structure.

**Figure 3:**
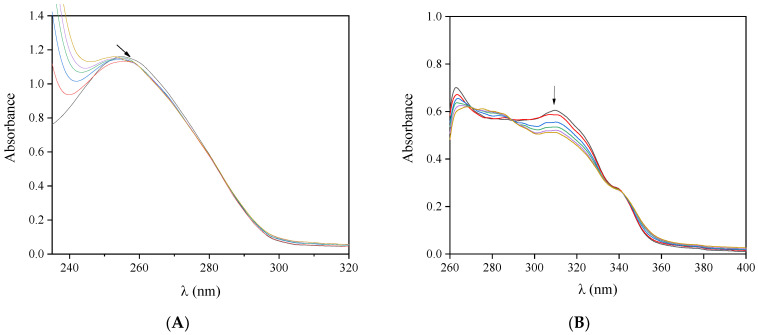
(**A**) UV spectra of CT DNA (0.18 mM) in buffer solution (150 mM NaCl and 15 mM trisodium citrate at pH 7.0) with increasing amounts of complex **3**. The arrow shows the changes upon the increasing amounts of the complexes. (**B**) UV-vis spectra of complex **3** (30 µM) in DMSO solution in the presence of increasing amount of CT DNA. The arrow shows the changes upon the addition of CT DNA.

The UV spectrum of a DNA solution presented in the presence of increasing amounts of complexes **3** (Figure 3A) and **6** a slight hypochromism accompanied by a slight red shift, while in the presence of complexes **1** and **2,** a hyperchromic effect was observed (Figure S4). In the UV-vis spectra of the complexes (Figure 3B and Figure S5), the intra-ligand bands exhibit in the presence of increasing amounts of CT DNA a slight decrease in absorbance (hypochromism) with a simultaneous red shift (Table 2). However, the percentages of the observed hyper- and hypochromism are quite low to lead to safe conclusions regarding the possible DNA-interaction mode. Thus, additional measurements were carried out such as DNA-viscosity measurements, cyclic voltammetry and EB-displacement studies.

The Wolfe–Shimer equation (Equation (S1)) [76] and the plots of [DNA]/(ε_A_ − ε_f_) vs. [DNA] (Figure S6) were used to calculate the K_b_ values of the complexes. The complexes bearing oxolinato ligands demonstrate a higher K_b_ value than free Hoxo, revealing its stronger binding to CT DNA upon coordination (Table 2). Complexes with the same *N*,*N′*-donor co-ligand (bipy for **1** and **3**, or phen for **2** and **6**) demonstrate similar DNA-binding constants, with complex **3** having the highest K_b_ (=2.68 × 10^5^ M^−1^). The magnitude of the DNA-binding constants (K_b_) of the complexes is similar to the known intercalator EB (K_b_ = 1.23 × 10^5^ M^−1^) [77]. Compounds with similar K_b_ values have been reported in the literature, highlighting the replacement of EB over complexes at the EB-DNA adduct, an indication of binding to DNA via intercalation [77]. Intercalation of the complexes is highly possible, since quinolone ligands and *N*,*N′*-donor co-ligands may provide a planar aromatic system to the complex. Similar interaction modes and binding constants have been reported in the literature for a series of transition metal complexes bearing quinolone ligands [14,15,16,18,20,23,53,55].

The mode of DNA-interaction may also be assessed using viscosity measurements. The DNA-length changes are quite sensitive during the titration with the simultaneous presence of increasing amounts of an interacting compound. Thus, viscometry is considered a useful and the least ambiguous method to evaluate the interaction modes of complexes with DNA in solution. In the case of interaction via intercalation, an elongation of the DNA-helix is observed; the DNA-base pairs start to disassemble to fit the new compound between them, thus increasing the DNA-viscosity. An interaction with the external surface of DNA, either electrostatically or via DNA-groove binding may lead to a short bend of the DNA-groove, resulting in a decrease in the length of the DNA-helix with a simultaneous decrease in viscosity, or it may not affect the DNA-length and the viscosity remains rather stable. In the case of DNA-cleavage, the compound ruptures the DNA-helix, leading to shorter fragments and a significant decrease in viscosity [78].

**Figure 4:**
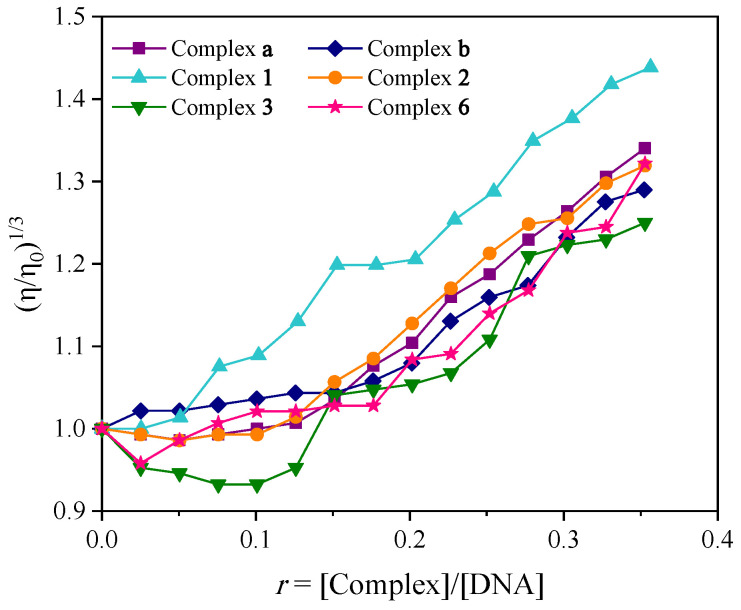
Relative viscosity (η/η_0_)^1/3^ of CT DNA (0.1 mM) in buffer solution (150 mM NaCl and 15 mM trisodium citrate at pH 7.0) in the presence of the complexes at increasing amounts (*r* = [complex]/[DNA]).

The DNA-viscosity changes of a CT DNA solution (0.1 mM) were assessed in the presence of increasing amounts of the compounds. In most cases, the addition of most complexes resulted (up to *r*~0.15, Figure 4) in a slight decrease in DNA-viscosity, suggesting that an external interaction with DNA may take place initially. Further addition of the complexes resulted in a gradual increase of the relative DNA-viscosity, revealing the existence of an intercalative interaction mode. Hence, we may suggest that the complexes interact with CT DNA probably via two combined modes: initially, externally (obviously electrostatically due to their cationic nature) and afterwards via intercalation (as a result of the π-π stacking interactions of the aromatic ligands in-between the DNA-bases) [78].

The interaction of DNA with metal complexes may also be monitored with cyclic voltammetry. The cyclic voltammetry experiments are carried out in order to assess the mode and strength of interaction of the reduced and oxidized form of metal in the presence of CT DNA. Thus, this technique may provide complementary information regarding the mechanism of interaction of metal complexes with DNA. The changes in the cyclic voltammograms were monitored upon incremental additions of CT DNA in a solution (1:2 DMSO:buffer) of the complex (0.33 mM). Electrostatic interactions with DNA may result in a negative shift in electrochemical potential, while intercalation may induce a positive shift in electrochemical potential. The absolute values of the shifts of these potentials may also indicate the strength of the interaction [79,80].

The cyclic voltammograms of the complexes were recorded in the absence and presence of DNA (Figure S7). For complexes **1**–**3**, the waves of the redox Co(III)/Co(II) were located at positive potentials, while those corresponding to couple Co(II)/Co(I) were found in the negative region of the cyclic voltammogram (Table 3). The positive shift of the potentials of the redox couple Co(II)/Co(I) for the complexes may suggest an intercalating fashion of interaction with CT DNA (Table 3). In addition, the presence of a negative shift of the second potential may suggest the co-existence of electrostatic interactions. In total, we may conclude that a combination of intercalation with electrostatic interactions may exist, leading to tighter binding [14,81].

Regarding the intensity of the current, in most cases (i.e., for complexes **1**–**3**), a significant decrease was observed (Figure S7). Among the cobalt–quinolone complexes, the most significant drop in the current intensity was observed for complex **3,** which also exhibited the highest K_b_ value. The ratio of the DNA-binding constants for the reduced (K_red_) and oxidized forms (K_ox_) of the metal ions (K_red_/K_ox_) was calculated according to Equation (S2) [82] and may be used to estimate the redox equilibrium. For most complexes, the ratio K_red_/K_ox_ is higher than 1 (Table 3) and may reveal that CT DNA may interact selectively with the oxidized form of the complexes [82].

Ethidium bromide (EB) is a well-known DNA intercalator since its planar phenanthridine ring can be inserted between the DNA bases and form an EB-DNA adduct [83,84]. An intense fluorescence emission band at 592 nm emerged, after excitation at 540 nm, and is attributed to the presence of EB-DNA adduct at the solution. The presence of compounds that tend to compete with EB-DNA adduct for the DNA-binding site may result in displacing the EB and may induce quenching of the EB-DNA fluorescence emission [85,86].

**Figure 5:**
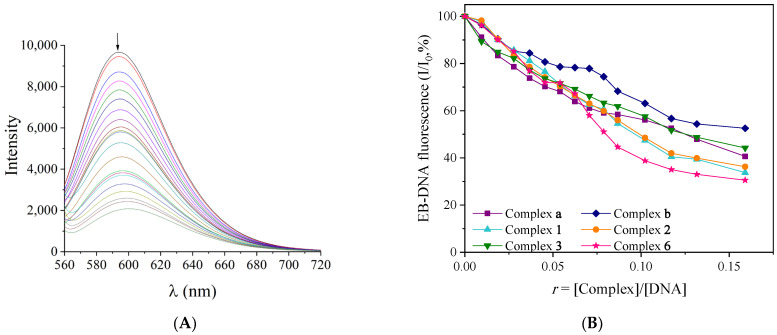
(**A**) Fluorescence emission spectra (λ_exc_ = 540 nm) for EB-DNA in buffer solution (150 mM NaCl and 15 mM trisodium citrate at pH 7.0) in the absence and presence of increasing amounts of complex **1**. (**B**) Plot of EB-DNA relative fluorescence intensity at λ_em_ = 592 nm (I/I_0_, %) vs. *r* (*r* = [complex]/[DNA]) for the compounds (up to 40.6% of the initial EB-DNA fluorescence intensity for **a**, 52.6% for **b**, 30.0% for **1**, 26.6% **2**, 35.5% **3**, and 27.8% **6**).

Fluorescence emission spectroscopy was used to study the ability of the synthesized complexes to antagonize EB for the EB-binding site of the EB-DNA adduct. A quenching of the emission band at 592 nm (Figure 5A and Figure S8) occurred upon the addition of complexes under study in an EB-CT DNA pretreated solution (40 μM EB, 40 μM CT DNA) and can be assigned to the displacement of the EB from EB-DNA (Figure 5B, Table 4). Hence, as the EB seems to be displaced by the complexes, the most probable mode of DNA interaction is intercalation.

The ability of the complexes to quench fluorescence was further evaluated via the calculation of Stern–Volmer constant (K_SV_) and quenching constant (k_q_) using the Stern–Volmer equations (Equation (S3)) and the corresponding Stern–Volmer plots (Figure S9). The high K_SV_ values indicate the ability of the complexes to antagonize and replace the EB as well as to tightly bind to DNA via intercalation with complex **1** having the highest value (Table 4). The K_sv_ values are in close range with the reported metal-quinolone ones [14,15,16,18,20,23,53,55]. Taking into consideration the value of 23 ns as the fluorescence lifetime of EB-DNA (τ_0_) [87], the k_q_ values were calculated with Equation (S4) [86]. The presence of a static quenching mechanism can be verified via the k_q_ values of the complexes (Table 4), which are higher than 10^10^ M^−1^s^−1^ [86]. A static quenching mechanism may indicate the formation of a new adduct between the studied complexes and DNA, indirectly confirming intercalation as the most possible mode of interaction.

### 2.4. Interaction of the Complexes with Serum Albumins

One of the most important and abundant proteins present in the human blood plasma of the circulatory system is serum albumin (SA). One of its key roles is the maintenance of the osmotic pressure, which is crucial to maintaining normal volume and water content levels of mesenchymal fluid and other tissues. The interaction of SA with quinolones or their compounds may lead to a loss or increase in their biological properties and/or assist in drug circulation, as albumin also has the ability to carry and bind in a reversible way to drugs or other biomolecules [88,89]. Thus, the aforementioned complexes were studied for their possible interaction with both HSA and BSA, which are homologs. An intense fluorescence emission band was observed in the spectra recorded for solutions of HSA (342 nm) and BSA (348 nm). The fluorescence emission spectra of SA (3 μM) buffer solution (15 mM trisodium citrate, 150 mM sodium chloride) were recorded in the presence of increasing amounts of the compounds (Figures S10 and S11). In terms of precision of the quantitative studies, the subtraction of the spectra was performed from the free compound. The inner-filter effect was further evaluated with Equation (S6), and it was negligible to affect the measurements [90].

**Figure 6:**
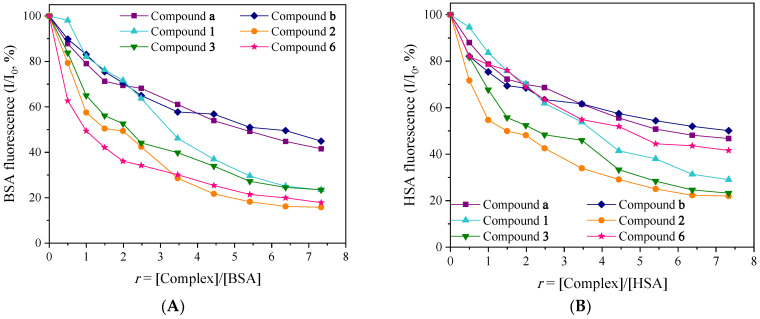
(**A**) Plot of relative fluorescence emission intensity of BSA at λ_em_ = 348 nm (I/I_0_, %) vs. *r* (*r* = [complex]/[BSA]) for the complexes (up to 23.5% of the initial BSA fluorescence for **1**, 15.8% for **2**, 23.5% for **3**, 17.8% for **6**, 41.5% for **a**, and 44.9% for **b**) in buffer solution. (**B**) Plot of relative fluorescence emission intensity of HSA at λ_em_ = 342 nm (I/I_0_, %) vs. *r* (*r* = [complex]/[HSA]) for the complexes (up to 29.7% of the initial HSA fluorescence for **1**, 23.0% for **2**, 23.2% for **3**, 41.6% for **6**, 46.7% for **a**, and 50.1% for **b**) in buffer solution.

Changes in protein conformation, the association of subunits, the binding of substrates or even denaturation may also affect the fluorescence spectra of the albumins. The addition of increasing amounts of a compound to these solutions results in a decrease in the intensity of the fluorescence emission band of the BSA and HSA. This quenching is an indication of the interaction of the compounds with the albumin. Thus, the resulting quenching from the changes in the secondary structure of the albumins may be attributed to possible alterations of tryptophan residues of SAs [86]. Specifically, all the quinolone complexes may induce a decrease in the fluorescence emission band for both HSA and BSA; complex **2** demonstrates the highest decrease in the emission fluorescence intensity of both albumins (Figure 6).

Further evaluation of the interaction was performed with the Stern–Volmer and Scatchard equations and the corresponding plots. The quenching (k_q_) constants were calculated with the Stern–Volmer equation (Equations (S3) and (S4), Figures S12 and S13) with τ_0_ = 10^−8^ s as the fluorescence lifetime of tryptophan in SAs. The k_q_ values for both albumins (Table 5) are much higher than 10^10^ M^−1^s^−1^, indicating the existence of a static quenching mechanism [86], which may confirm the interaction of the albumin and the complexes. The SA-binding constants (K) were calculated with the Scatchard equation (Equation (S6)) and corresponding plots (Figures S14 and S15) and their magnitude (of the 10^5^ M^−1^) may reveal a rather tight binding of the complexes with the albumins. The derived SA-quenching and SA-binding constants of the complexes (Table 5) are comparable with reported transition metal-quinolone complexes ligands [14,16,18,20,23,53,55].

Avidin demonstrates binding constants (K_avid_) with various compounds at about 10^15^ M^−1^, which is considered the highest value among all known non-covalent interactions. The K values of all complexes under study are significantly lower than K_avid_; hence, these values may indicate that the quinolone complexes can bind reversibly to the albumins, and they can be released by SAs to the intended targets [91].

## 3. Experimental

### 3.1. Materials—Instrumentation—Physical Measurements

All chemicals and solvents were purchased from commercial sources and were used as received. Hoxo, Hflmq, Hcx, Hppa, CoCl_2_·6H_2_O, phen, bipy, NH_4_PF_6_, KOH, HCl (35% *v*/*v*), NaCl, trisodium citrate, CT DNA, BSA, HSA, and EB were purchased from Sigma-Aldrich (Saint Louis, MO, USA). All solvents were reagent grade and purchased from Chemlab (Zedelgem, Belgium). [Co(bipy)_2_Cl_2_]Cl and [Co(phen)_2_Cl_2_](H_2_O) were synthesized according to literature procedures [92].

Infrared (IR) spectra were recorded with samples prepared as KBr pellets in the range of 400–4000 cm^−1^ on a Nicolet (Madison, WI, USA) FT-IR 6700 spectrometer (abbreviations used: (vs) for very strong; (s) for strong; (m) for medium; (w) for weak; Δν(COO) = ν_asym_(COO) − ν_sym_(COO)). UV-visible (UV-vis) spectra were recorded in solution with a concentration range between 10^−5^ and 5 × 10^−3^ M on a Hitachi (Hitachi, Tokyo, Japan) U-2001 dual-beam spectrophotometer. ^1^H NMR spectra were recorded on a Bruker (Billerica, MA, USA) 300 and 400 MHz spectrometer, with trimethylsilane (TMS) δH = 0 ppm, or residual protic solvent peak [DMSO-d_6_, δH = 2.50 ppm; CD_3_OD, δH = 3.31 ppm; D_2_O, δH = 4.79 ppm] as the internal standard. The numbering of the hydrogen atoms used for the assignment of the NMR spectra is given in Figure S2. Chemical shifts are given in ppm (δ) and MestreNova v14.1.2-25024 software was used to analyze the NMR spectra. The fluorescence spectra of the compounds were recorded on a Hitachi F-7000 fluorescence spectrometer (Hitachi, Tokyo, Japan). C, H and N elemental analysis was performed on a Perkin–Elmer (PerkinElmer, Waltham, MA, USA) 240B elemental analyzer. Molar conductivity measurements were carried out at room temperature (25 °C) in a type-C cuvette in DMSO-complex solution (1 mM) monitored on a Crison Basic 30 conductometer (Crison Instruments, Barcelona, Spain). Viscosity experiments were carried out using an ALPHA L Fungilab rotational viscometer (Fungilab S.A., Barcelona, Spain) equipped with an 18-mL LCP spindle and the measurements were performed at 100 rpm.

Cyclic voltammetry assays were performed on an Eco ChemieAutolab Electrochemical analyzer (Utrecht, The Netherlands). A three-electrode assembly with an electrolytic cell (30 mL) containing a Pt working electrode (for oxidation), a separate Pt single-sheet auxiliary electrode and an Ag/AgCl reference electrode saturated with KCl, was deployed for the experiments. The cyclic voltammograms of the complexes were recorded in 0.4 mM DMSO:buffer solutions (1:2) at a scan rate of v = 100 mM s^−1^, where the buffer was the supporting electrolyte. Oxygen was removed by purging the solutions with pure nitrogen, which had been previously saturated with solvent vapors. All electrochemical measurements were performed at room temperature (25 °C).

CT DNA stock solution was prepared by dilution of CT DNA in buffer (150 mM NaCl and 15 mM trisodium citrate). The solution was stirred vigorously for three days at 4 °C and was kept at 4 °C for no longer than ten days. The stock solution gave a ratio of UV absorbance at 260 and 280 nm (A_260_/A_280_) of ~1.90, an indication that the DNA is adequately free of protein contamination [93]. The DNA concentration was determined after 1:20 dilution by measuring the absorbance at 260 nm (ε = 6600 M^−1^ cm^−1^) [94].

### 3.2. Synthesis of the Complexes

#### 3.2.1. Synthesis of Compounds [Co(bipy)_2_Cl_2_]Cl (**a**) and [Co(phen)_2_Cl_2_](H_2_O) (**b**)

For the synthesis of complexes **1**–**6**, [Co(bipy)_2_Cl_2_]Cl (**a**) and [Co(phen)_2_Cl_2_](H_2_O) (**b**) were used as precursor compounds and were synthesized according to a modified procedure of that described in the literature [65,67]. Specifically, a methanolic solution (10 mL) of bipy (2 mmol, 312 mg) or phen (2 mmol, 360 mg) was mixed slowly with a methanolic solution (5 mL) of CoCl_2_·6H_2_O (1 mmol, 290 mg) under stirring. The solution was stirred for 1 h under oxygen bubbling. A small amount of concentrated HCl (12 M, 37% *v*/*v*) was then added, and the solution was continually stirred for an additional hour. The solutions were allowed to rest for a few days affording purple and/or dark green single-crystals of [Co(bipy)_2_Cl_2_]Cl (**a**) (286 mg, 47.3%, indicating a dichroism effect [66]), or dark red crystals of [Co(phen)_2_Cl_2_](H_2_O) (**b**) (193 mg, 47.5%). Single-crystals for both compounds, suitable for X-ray crystallography, were isolated in order to confirm their structures. These complexes **a** and **b** were further employed as precursor compounds in the synthesis of complexes **1**–**6**.

#### 3.2.2. Synthesis of Complexes [Co(N,N′-donor)_2_(Q)](PF_6_)_x_ (x = 2 for **1**–**5** and x = 1 for **6**)

For the synthesis of complexes **1**–**6**, an aqueous solution of the appropriate precursor **a** or **b** (0.05 mol) was mixed with a methanolic solution containing the corresponding quinolone HQ (0.05 mmol) and KOH (0.05 mmol, 0.05 mL 1 M). After 1 h of stirring, the pH of the solution was adjusted with KOH to a value of 9, and the solution was refluxed at 60 °C overnight. Afterwards, NH_4_PF_6_ (0.10 mmol, 16 mg) dissolved in MeOH was added, and the solution was left to slowly evaporate at room temperature. Single-crystals of complexes **1**–**6** suitable for X-ray crystallography were observed after diverse time periods.

[Co(bipy)_2_(oxo)](PF_6_)_2_·H_2_O, (**1**): For the synthesis of complex **1**, complex **a** (0.05 mmol, 24 mg) was used as starting material, and Hoxo (0.05 mmol, 13 mg) was the corresponding HQ. Orange-red single-crystals (28 mg, yield: 60%) of **1** suitable for X-ray determination were obtained after three weeks. Anal. Calc. for [Co(bipy)_2_(oxo)](PF_6_)_2_·H_2_O (C_33_H_28_CoF_12_N_5_O_6_P_2_) (MW = 939.47): C, 42.19; H, 3.00; N, 7.45%. Found: C, 42.34; H, 3.15; N, 7.28%. IR (KBr disk), ν_max_ (cm^−1^): ν_asym_(COO), 1648 (s); ν(C=O)_pyridone_, 1628 (s); ν_sym_(COO), 1410 (νs); Δν(COO) = 238; ν(PF_6_) = 843 (s); ρ(C-H)_bipy_ = 773 (m). ^1^H NMR (95:5 D_2_O:DMSO-d_6_), δ (ppm): 8.98 (s, 1H, H^3,oxo^), 8.53–8.82 (m, 8H, H^bipy^), 8.35–8.40 (m, 2H, H^bipy^), 7.91–7.98 (m, 2H, H^bipy^), 7.57 (m, 4H, H^bipy^), 7.45 (s, 1H, H^6,oxo^), 7.24 (s, 1H, H^5,oxo^), 6.21 (d, 2H, H^4,oxo^), 4.56 (q, 2H, H^2,oxo^), 1.52 (t, 3H, H^1,oxo^). UV-vis in DMSO, λ/nm (ε/M^−1^cm^−1^): 496 (180), 344 (10,190), 315 (15,900), 304 (16,200). The complex is soluble in DMSO, MeOH, EtOH, and DMF, and partially soluble in H_2_O, CH_3_CN, CH_2_Cl_2,_ and acetone. Λ_Μ_ (in 1 mM DMSO solution) = 118 mho·cm^2^·mol^−1^.

[Co(phen)_2_(oxo)](PF_6_)_2_·0.5MeOH·0.5H_2_O, (**2**): In order to obtain complex **2**, complex **b** (0.05 mmol, 25 mg) was used as a precursor compound, and Hoxo (0.05 mmol, 13 mg) was the corresponding HQ. Orange-red single-crystals (25 mg, yield 50%) of **2** suitable for X-ray determination were obtained after 20 days. Anal. Calc. for [Co(phen)_2_(oxo)](PF_6_)_2_·0.5MeOH· 0.5H_2_O (C_37.5_H_29_CoF_12_N_5_O_6_P_2_) (MW = 994.53): C, 45.29; H, 2.94; N, 7.04%. Found: C, 45.49; H, 2.75; N, 6.90%. IR (KBr disk), ν_max_ (cm^−1^): ν_asym_(COO), 1647 (s); ν(C=O)_pyridone_, 1618 (s); ν_sym_(COO), 1414 (s); Δν(COO) = 233; ν(PF_6_) = 841 (vs); ρ(C-H)_bipy_ = 718 (m). ^1^H NMR (95:5 D_2_O:DMSO-d_6_), δ (ppm): 9.17 (m, 3H, H^phen^), 9.04 (s, 1H, H^3,oxo^), 8.84–8.98 (m, 3H, H^phen^), 8.26–8.44 (m, 6H, H^phen^), 7.63–7.69 (m, 4H, H^phen^) 7.44 (s, 1H, H^6,oxo^), 7.06 (s, 1H, H^5,oxo^), 6.14 (d, 2H, H^4,oxo^), 4.58 (q, 2H, H^2,oxo^), 1.53 (t, 3H, H^1,oxo^). UV-vis in DMSO, λ/nm (ε/M^−1^cm^−1^): 514 (182), 344 (9873). The complex is soluble in DMSO, MeOH, EtOH, and DMF, and partially soluble in H_2_O, CH_3_CN, CH_2_Cl_2,_ and acetone. Λ_Μ_ (in 1 mM DMSO solution) = 115 mho·cm^2^·mol^−1^.

[Co(bipy)_2_(flmq)](PF_6_)_2_·0.5MeOH·0.5H_2_O, (**3**): For the synthesis of complex **3**, complex **a** (0.05 mmol, 24 mg) was used as starting material, and Hflmq (0.05 mmol, 13 mg) was the corresponding HQ. Dark red single-crystals (28 mg, yield 60%) of **3** suitable for X-ray determination were obtained after three weeks. Anal. Calc. for [Co(bipy)_2_(flmq)](PF_6_)_2_·0.5MeOH· 0.5H_2_O (C_34.50_H_30_CoF_13_N_5_O_4_P_2_) (MW = 946.50): C, 43.78; H, 3.19; N, 7.40%. Found: C, 44.02; H, 3.01; N, 7.35%. IR (KBr disk), ν_max_ (cm^−1^): ν_asym_(COO), 1638 (s); ν(C=O)_pyridone_, 1609 (s); ν_sym_(COO), 1426 (s); Δν(COO) = 212; ν(PF_6_) = 841 (vs); ρ(C-H)_bipy_ = 769 (m). ^1^H NMR (95:5 D_2_O:DMSO-d_6_), δ (ppm): 9.12 (s, 1H, H^7,flmq^), 8.55–8.84 (m, 8H, H^bipy^), 8.36–8.41 (m, 2H, H^bipy^), 8.00 (m, 1H, H^6,flmq^), 7.94 (m, 1H, H^5,flmq^), 7.50–7.61 (m, 6H, H^bipy^), 4.93 (m, 1H, H^2,flmq^), 3.20 (m, 3H, H^4,flmq^), 2.25 (m, 2H, H^3,flmq^), 1.51 (t, 3H, H^1,flmq^). UV-vis in DMSO, λ/nm (ε/M^−1^cm^−1^): 500 (98), 343 (9470), 311 (20,300). The complex is soluble in DMSO, MeOH, EtOH, and DMF, and partially soluble in H_2_O, CH_3_CN, CH_2_Cl_2,_ and acetone. Λ_Μ_ (in 1 mM DMSO solution) = 105 mho·cm^2^·mol^−1^.

[Co(bipy)_2_(ppa)](PF_6_)_2_·MeOH·H_2_O, (**4**): In order to obtain complex **4**, complex **a** (0.05 mmol, 24 mg) was used precursor compound, and Hppa (0.05 mmol, 13 mg) was the corresponding HQ. Dark red single-crystals (15 mg, yield 30%) of complex **4,** suitable for X-ray determination, were obtained after two months of slow evaporation. Anal. Calc. for [Co(bipy)_2_(ppa)](PF_6_)_2_· MeOH·H_2_O (C_35_H_38_CoF_12_N_9_O_5_P_2_) (MW = 1013.60): C, 41.47; H, 3.78; N, 12.44%. Found: C, 41.21; H, 3.89; N, 12.19%. IR (KBr disk), ν_max_ (cm^−1^): ν_asym_(COO), 1643 (s); ν(C=O)_pyridone_, 1618 (s); ν_sym_(COO), 1452 (νs); Δν(COO) = 191; ν(PF_6_) = 841 (vs); ρ(C-H)_bipy_ = 769 (m). ^1^H NMR (DMSO-d_6_), δ (ppm): 9.22 (s, 1H, H^6,ppa^), 8.96–9.11 (m, 4H, H^bipy^), 8.93 (s, 1H, H^5,ppa^), 8.69 (m, 4H, H^bipy^), 8.46 (m, 2H, H^bipy^), 7.99–8.10 (m, 2H, H^bipy^), 7.65 (m, 3H, H^bipy^), 4.48 (q, 2H, H^2,ppa^), 4,07 (m, 4H, H^4,ppa^), 3,17 (m, 4H, H^3,ppa^), 1,36 (t, 3H, H^1,ppa^). UV-vis in DMSO, λ/nm (ε/M^−1^cm^−1^): 499 (138), 366 (7500), 320 (33,750), 286 (60,700). The complex is soluble in DMSO, MeOH, EtOH, and DMF, and partially soluble in H_2_O, CH_3_CN, CH_2_Cl_2,_ and acetone. Λ_Μ_ (in 1 mM DMSO solution) = 125 mho·cm^2^·mol^−1^.

[Co(phen)_2_(cx)](PF_6_)_2_·MeOH·0.5H_2_O, (**5**): For the preparation of complex **5**, complex **b** (0.05 mmol, 25 mg) was used as the precursor compound, and Hcx (0.05 mmol, 13 mg) was the corresponding HQ. Orange-reddish single-crystals (10 mg, yield 20%) of complex **5**, suitable for X-ray determination, were obtained after three months of slow evaporation. Anal. Calc. for [Co(phen)_2_(cx)](PF_6_)_2_·MeOH·0.5H_2_O (C_37_H_30_CoF_12_N_6_O_6.5_P_2_) (MW = 1011.54): C, 43.93; H, 2.99; N, 8.31%. Found: C, 44.23; H, 2.78; N, 8.12%. IR (KBr disk), ν_max_ (cm^−1^): ν_asym_(COO), 1651 (s); ν(C=O)_pyridone_, 1610 (s); ν_sym_(CO_2_), 1433 (s); Δν(CO_2_) = 218; ν(PF_6_) = 840 (vs); ρ(C-H)_bipy_ = 717 (m). ^1^H NMR (95:5 D_2_O:DMSO-d_6_), δ (ppm): 9.00–9.22 (m, 6H, H^phen^), 8.85–8.89 (dd, 2H, H^phen^), 8.28–8.45 (m, 6H, H^phen^), 7.68 (m, 4H, H^phen^), 7.58 (s, 1H, H^5,cx^), 7.05 (s, 1H, H^4,cx^), 6.25 (d, 2H, H^3,cx^), 4.87 (m, 2H, H^2,cx^), 1.58 (t, 3H, H^1,cx^). The complex is soluble in DMSO, MeOH, EtOH, and DMF, and partially soluble in H_2_O, CH_3_CN, CH_2_Cl_2,_ and acetone.

[Co(phen)_2_(flmq)](PF_6_)·0.5MeOH·H_2_O, (**6**): In order to prepare complex **6**, complex **b** (0.05 mmol, 25 mg) was used as the precursor compound, and Hflmq (0.05 mmol, 13 mg) was the corresponding HQ. Dark red single-crystals (26 mg, yield 60%) of complex **6**, suitable for X-ray determination, were obtained after three weeks of slow evaporation. Anal. Calc. for [Co(phen)_2_(flmq)](PF_6_)·0.5MeOH·H_2_O (C_38.5_H_31_CoF_7_N_5_O_4.5_P) (MW = 858.59): C, 53.86; H, 3.64; N, 8.16%. Found: C, 54.02; H, 3.76; N, 7.99%. IR (KBr disk), ν_max_ (cm^−1^): ν_asym_(COO), 1644 (s); ν(C=O)_pyridone_, 1627 (s); ν_sym_(COO), 1429 (s); Δν(COO) = 215; ν(PF_6_) = 841 (vs); ρ(C-H)_phen_ = 717 (m). UV-vis in DMSO, λ/nm (ε/M^−1^cm^−1^): 506 (89), 344 (10,420), 330 (12,860). The complex is soluble in DMSO, MeOH, EtOH, and DMF, and partially soluble in H_2_O, CH_3_CN, CH_2_Cl_2,_ and acetone. Λ_Μ_ (in 1 mM DMSO solution) = 60 mho·cm^2^·mol^−1^.

### 3.3. Crystallographic Data Collection and Structure Determination

Slow evaporation of methanolic solutions of **1**–**6** afforded suitable single-crystals analyzed by single-crystal X-ray diffraction. The crystals were mounted on a Bruker Kappa APEX II X-ray diffractometer (Bruker, Billerica, MA, USA), equipped with a TRIUMPH monochromator. Radiation from a Mo source was used to record the diffraction measurements at room temperature. A minimum of 130 reflections in the range of 15 < θ < 20° were employed to accomplish cell dimensions refinement, while φ and ω scan modes were used to collect intensity data. Bruker SAINT software version 1.1 was used to process the reflection data for each crystal via a narrow-frame algorithm [95]. The SADABS numerical method, based on the dimensions of the crystals, was used to correct the data for absorption [96]. All structures were solved by charge flipping methods implemented in Superflip [97] and refined by a full matrix least-squares procedure based on *F*^2^ using CRYSTALS software v14.61_build_6236 [98].

All asymmetric units in all crystals contain a cationic main complex, PF_6_^−^ counter anions and/or water and methanol solvate molecules. Non-hydrogen atoms in all main complexes were non-disordered and refined anisotropically (except the terminal piperazine part in complex **4**, which was found disordered over two positions with equal occupation factors). Most of the hexafluorophosphate anions and solvate molecules were found disordered over two or even more positions. All non-hydrogen, non-disordered atoms were refined anisotropically. For the disordered atoms, their occupation factors and positions under fixed isotropic parameters (Uiso = 0.05) were first refined. After this step, all refined isotropically were under fixed occupation factors.

Hydrogen atoms riding on parent non-disordered atoms were located on their expected positions and refined with isotropic displacement parameters Uiso(H) = 1.2Ueq(C) or 1.5Ueq(methyl, -NH and -OH hydrogens) and at distances C-H 0.95 Å, N-H 0.83 Å and O-H 0.82 Å. All methyl, amine and OH hydrogen atoms were allowed to rotate. Hydrogen atoms riding on disordered oxygen atoms of methanol and water solvent molecules were positioned geometrically to fulfill hydrogen bonding demands. The rest methyl hydrogen atoms were positioned geometrically to their parent atoms. Crystallographic data are listed in Table S1.

CCDC deposition numbers 2,451,691–2,451,696 contain the supplementary crystallographic data for this paper. These data can be obtained free of charge via www.ccdc.cam.ac.uk (or from the Cambridge Crystallographic Data Centre, 12 Union Road, Cambridge CB21EZ, UK; fax: (+44) 1223-336-033; or deposit@ccdc.cam.ac.uk).

### 3.4. Study of the In Vitro Biological Profile of the Compounds

The interaction of the compounds with DNA and albumins was evaluated in vitro after the compounds were dissolved in DMSO (1 mM), due to their low solubility in water. The studies were performed in the presence of aqueous buffer solutions, where the mixing of each solution did not exceed 5% DMSO (*v*/*v*) in the final solution. The effect of DMSO on the data was monitored through control experiments. The spectra of the albumins SAs or CT DNA did not present any appreciable differences, and the relevant appropriate corrections were carried out where necessary.

UV-vis spectroscopy, viscosity measurements, and cyclic voltammetry, as well as competitive studies with EB by fluorescence emission spectroscopy, were employed to screen the interaction of the compounds with CT DNA. Tryptophan fluorescence quenching experiments were carried out to assess the binding of the compounds with serum albumins. Detailed procedures of the in vitro biological activity studies are given in the Supporting Information file (Sections S1–S2) [76,82,86,87,90,99,100].

## 4. Conclusions

Six cationic cobalt complexes of the general formula [Co(*N*,*N′*-donor)_2_(Q)](PF_6_)_x_, where x = 1 or 2, were synthesized via the reaction of complexes [Co(bipy)_2_Cl_2_]Cl and [Co(phen)_2_Cl_2_](H_2_O), which were used as precursor compounds with the quinolones oxolinic acid, flumequine, pipemidic acid and cinoxacin, and were characterized successfully by diverse spectroscopies and single-crystal X–ray crystallography. Five of the six complexes were found to be dicationic Co(III) complexes bearing the formula [Co(N,N′-donor)_2_(Q)](PF_6_)_2_ (namely [Co(bipy)_2_(oxo)](PF_6_)_2_·H_2_O (**1**), [Co(phen)_2_(oxo)](PF_6_)_2_·0.5CH_3_OH·0.5H_2_O (**2**), [Co(bipy)_2_(flmq)](PF_6_)_2_·0.5CH_3_OH·0.5H_2_O (**3**), [Co(bipy)_2_(ppa)](PF_6_)_2_·CH_3_OH·H_2_O (**4**), and [Co(phen)_2_(cx)](PF_6_)_2_·CH_3_OH·0.5H_2_O (**5**)), while complex **6** with the formula [Co(phen)_2_(flmq)](PF_6_)·0.5CH_3_OH·H_2_O is a monocationic Co(II) complex. In all these complexes, the quinolone ligands are bound to the metal in a bidentate fashion through the pyridone oxygen and carboxylate oxygen.

The biological profile of the resultant complexes **1**–**6** was evaluated for their affinity for CT DNA and their binding with serum albumins by diverse techniques. The interaction of the complexes with CT DNA is rather tight (based on the magnitude of DNA-binding constants) and may take place via a combination of intercalation (due to π-π stacking interaction developed between the aromatic ligands of the complexes and DNA bases) and electrostatic interactions (stemming from the cationic nature of the complexes) with the external phosphate groups of CT DNA. All studied complexes exhibited strong and reversible binding to both albumins (BSA and HSA), with albumin-binding constants in the range of 10^4^–10^5^ M^−1^. Such tight yet reversible interactions are considered essential for potential pharmacological applications, as they enable efficient transport via albumins and facilitate the controlled release of the complexes at their biological targets.

These findings highlight the potential of cationic cobalt complexes of first-generation quinolones as promising scaffolds for further exploration in bioinorganic and medicinal chemistry. The incorporation of π-conjugated co-ligands appears to enhance biomolecular recognition, facilitating the modulation of the biological activity of known antibacterial agents through metal coordination. Future work may focus on their antimicrobial and cytotoxic properties toward developing cobalt-based therapeutic candidates.

## Figures and Tables

**Table 1:** Selected bond lengths (Å) and bond angles (°) for complexes **1**–**6**.

Compound	1–5	6
**Bond**	**Length (Å)**	**Length (Å)**
Co1—O1	1.879 (2)–1.901 (2)	1.923 (2)
Co1—O3	1.878 (2)–1.9055 (19)	1.9235 (19)
Co1—N1	1.927 (3)–1.962 (3)	2.042 (3)
Co1—N2	1.922 (3)–1.940 (3)	2.018 (2)
Co1—N3	1.926 (3)–1.963 (2)	2.034 (3)
Co1—N4	1.904 (3)–1.964 (3)	2.031 (3)
**Bonds**	**Angle (°)**	**Angle (°)**
O1—Co1—O3	94.09 (10)–95.28 (9)	91.97 (9)
O1—Co1—N *trans*	174.47 (11)–177.31 (11)	174.35 (10)
O1—Co1—N *cis*	85.58 (10)–94.63 (11)	90.40 (10)–93.55 (9)
O3—Co1—N *trans*	174.27 (11)–177.35 (11)	173.30 (10)
O3—Co1—N *cis*	81.97 (12)–94.54 (11)	87.29 (9)–92.71 (10)
N—Co1—N chelate	81.97 (12)–84.46 (13)	80.88 (12)–81.09 (11)
N—Co1—N *trans*	175.77 (11)–177.92 (12)	171.33 (11)
N—Co1—N *cis*	90.59 (12)–97.07 (11)	90.96 (10)–93.83 (11)

**Table 2:** UV-vis spectra feature of the interaction of the complexes with CT DNA. UV-band (λ_max_, in nm) (percentage of the observed hyper-/hypo-chromism (ΔA/A_0_, in %), blue-/red-shift of the λ_max_ (Δλ_max_, in nm)) and DNA-binding constants (K_b_, in M^−1^).

Compound	λ_max_ (nm) (ΔA/A_0_ (%)) ^a^, Δλ_max_ (nm) ^b^)	K_b_ (M^−1^)
Hoxo [15]	324(+50 ^a^), 334(+45 ^a^)	3.02(±0.10) × 10^3^
Hflmq [17,18]	326 (−15); 340 (+7)	3.53(±0.45) × 10^5^
[Co(bipy)_2_(oxo)](PF_6_)_2_, (**1**)	315 (−11, +4)	2.03(±0.19) × 10^5^
[Co(phen)_2_(oxo)](PF_6_)_2_, (**2**)	344 (−10, +1)	1.07(±0.08) × 10^4^
[Co(bipy)_2_(flmq)](PF_6_)_2_, (**3**)	310 (−15, +1)	2.68(±0.15) × 10^5^
[Co(phen)_2_(flmq)](PF_6_), (**6**)	325 (+5, 0)	2.09(±0.18) × 10^4^
[Co(bipy)_2_Cl_2_]Cl, (**a**)	296 (+4.5, +3); 315 (−30, +10)	1.27(±0.12) × 10^6^
[Co(phen)_2_Cl_2_](H_2_O), (**b**)	278 (−3, 0)	8.61(±0.11) × 10^6^

^a^ “+” denotes hyperchromism., “−” denotes hypochromism. ^b^ “+” denotes red-shift, “−” denotes blue-shift.

**Table 3:** Cathodic and anodic potentials (mV) for the redox couples Co(II)/Co(I) of the complexes in 1:2 DMSO:buffer solution in the presence and absence of CT DNA. Ratio of equilibrium binding constants (K_r_/K_ox_).

Complex	E_pc(f)_ ^a^	E_pc(b)_ ^b^	ΔE_pc_ ^c^	E_pa(f)_ ^a^	E_pa(b)_ ^b^	ΔE_pa_ ^c^	$E_{\left(b\right)}^{o}$ ^d^	$E_{\left(f\right)}^{o}$ ^d^	K_red_/K_ox_
[Co(bipy)_2_(oxo)](PF_6_)_2_, **1**	−726	−714	+12	−497	−521	−24	−617.5	−611.5	0.90
[Co(phen)_2_(oxo)](PF_6_)_2_, **2**	−732	−723	+9	−474	−437	+37	−580	−603	1.48
[Co(bipy)_2_(flmq)](PF_6_)_2_, **3**	−740	−730	+10	−403	−388	+15	−559	−571.5	1.24
[Co(phen)_2_(flmq)](PF_6_), **6**	−791	−798	−7	−388	−378	+10	−588	−589.5	1.03
[Co(bipy)_2_Cl_2_]Cl, **a**	−762	−764	−2	−408	−479	−71	−621.5	−585	0.54
[Co(phen)_2_Cl_2_](H_2_O), **b**	−842	−945	−103	−425	−420	+5	−682.5	−633.5	0.44

^a^ Ep_c/a_ in DMSO/buffer in the absence of CT DNA (Ep_c/a(f)_). ^b^ Ep_c/a_ in DMSO/buffer in the presence of CT DNA (Ep_c/a(b)_). ^c^ ΔEp_c/a_ = Ep_c/a(b)_ − Ep_c/a(f)_. ^d^ $E_{\left(b\right)}^{o}$ and $E_{\left(f\right)}^{o}$ are the formal potentials (E^o^_(b/f)_ = (E_pc(b/f)_ + E_pa(b/f)_)/2) used for the calculation of the ratio K_red_/K_ox_ with Equation (S2).

**Table 4:** Fluorescence features of the EB-displacement studies for the compounds. Percentage of EB-DNA fluorescence emission quenching (ΔI/I_0_, in %), Stern–Volmer constants (K_SV_, in M^−1^), and quenching constants (k_q_, in M^−1^s^−1^).

Compound	ΔI/I_0_ (%)	K_SV_ (M^−1^)	k_q_(M^−1^s^−1^)
Hoxo [15]	~0	Not defined	-
Hflmq [17,18]	55.0	1.19(±0.06) × 10^6^	5.17(±0.26) × 10^13^
[Co(bipy)_2_(oxo)](PF_6_)_2_, **1**	70.0	3.77(±0.12) × 10^5^	1.64(±0.05) × 10^13^
[Co(phen)_2_(oxo)](PF_6_)_2_, **2**	73.4	2.63(±0.08) × 10^5^	1.14(±0.03) × 10^13^
[Co(bipy)_2_(flmq)](PF_6_)_2_, **3**	64.5	2.87(±0.07) × 10^5^	1.25(±0.03) × 10^13^
[Co(phen)_2_(flmq)](PF_6_), **6**	72.8	3.28(±0.12) × 10^5^	1.42(±0.05) × 10^13^
[Co(bipy)_2_Cl_2_]Cl, **a**	59.4	1.58(±0.03) × 10^5^	6.88(±0.15) × 10^12^
[Co(phen)_2_Cl_2_](H_2_O), **b**	47.4	1.02(±0.02) × 10^5^	4.44(±0.10) × 10^12^

**Table 5:** Fluorescence features of the albumin-interaction studies for the compounds. Percentage of albumin-fluorescence emission quenching (ΔI/I_0_, in %), albumin-quenching constants (k_q_, in M^−1^s^−1^) and albumin-binding constants (K, in M^−1^).

Compound	ΔI/I_0_ (%)	k_q_ (M^−1^s^−1^)	K (M^−1^)
**BSA**			
Hoxo [15]		5.01(±0.22) × 10^12^	1.09(±0.09) × 10^5^
Hflmq [17,18]		8.26(±0.36) × 10^12^	6.67 × 10^4^
[Co(bipy)_2_(oxo)](PF_6_)_2_, **1**	76.5	1.59(±0.07) × 10^13^	2.38(±0.14) × 10^4^
[Co(phen)_2_(oxo)](PF_6_)_2_, **2**	84.2	2.80(±0.10) × 10^13^	1.39(±0.05) × 10^5^
[Co(bipy)_2_(flmq)](PF_6_)_2_, **3**	76.5	1.53(±0.05) × 10^13^	1.87(±0.08) × 10^5^
[Co(phen)_2_(flmq)](PF_6_), **6**	81.2	1.97(±0.07) × 10^13^	4.93(±0.23) × 10^5^
[Co(bipy)_2_Cl_2_]Cl, **a**	58.5	7.27(±0.22) × 10^11^	1.13(±0.04) × 10^4^
[Co(phen)_2_Cl_2_](H_2_O), **b**	55.1	6.49(±0.17) × 10^11^	9.00(±0.33) × 10^3^
**HSA**			
Hoxo [15]		6.39(±0.26) × 10^12^	1.13(±0.20) × 10^5^
Hflmq [17,18]		1.00(±0.17) × 10^13^	2.37 × 10^6^
[Co(bipy)_2_(oxo)](PF_6_)_2_, **1**	70.3	1.16(±0.04) × 10^13^	3.76(±0.09) × 10^4^
[Co(phen)_2_(oxo)](PF_6_)_2_, **2**	77.0	1.75(±0.05) × 10^13^	3.13(±0.12) × 10^5^
[Co(bipy)_2_(flmq)](PF_6_)_2_, **3**	76.8	1.53(±0.03) × 10^13^	1.49(±0.04) × 10^5^
[Co(phen)_2_(flmq)](PF_6_), **6**	59.4	6.25(±0.25) × 10^12^	1.16(±0.04) × 10^5^
[Co(bipy)_2_Cl_2_]Cl, **a**	53.3	6.16(±0.20) × 10^12^	1.31(±0.05) × 10^5^
[Co(phen)_2_Cl_2_](H_2_O), **b**	49.9	4.49(±0.12) × 10^12^	2.38(±0.07) × 10^5^

## Data Availability

All necessary supplementary data are included in the supplementary files.

## Supplementary Materials

The following supporting information can be downloaded at: https://www.mdpi.com/article/10.3390/molecules30122646/s1, Cif and checkcif files for compounds **1**–**6**. Protocols and equations regarding binding studies with CT DNA (Section S1), and albumin–binding studies (Section S2), Tables S1–S3 and Figures S1–S15 are included in the ESI file [76,82,86,87,90,99,100].

## Abbreviations

The following abbreviations are used in this manuscript:

bipy	2,2′-bipyridine
BSA	bovine serum albumin
CT	calf-thymus
cx^−^	anion of cinoxacin
EB	ethidium bromide
flmq^−^	anion of flumequine
Hcx	cinoxacin
Hflmq	flumequine
Hoxo	oxolinic acid
Hppa	pipemidic acid
HQ	quinolone
HSA	human serum albumin
K	SA-binding constant
K_avid_	binding constant with avidin
K_b_	DNA-binding constant
K_ox_	DNA-binding constant for the oxidized form of the complex
k_q_	quenching constant
K_red_	DNA-binding constant for the reduced form of the complex
K_SV_	Stern–Volmer constant
oxo^−^	oxolinate anion
phen	1,10-phenanthroline
ppa^−^	pipemidate anion
Q^−^	anion of quinolone
Δν(COO)	ν_asym_(COO) − ν_sym_(COO)

## References

[1] Andriole V.T. (2000). The Quinolones.

[2] Millanao A.R., Mora A.Y., Villagra N.A., Bucarey S.A., Hidalgo A.A. (2021). Biological Effects of Quinolones: A Family of Broad-Spectrum Antimicrobial Agents. Molecules.

[3] Newman R.L., Holt R.J., Frankcombe C.H. (1966). Nalidixic Acid: Microbiological and Clinical Studies on Urinary Infections in Children. Arch. Dis. Child..

[4] Wagman A.S., Wentland M.P. (2007). Quinolone Antibacterial Agents. Compr. Med. Chem. II.

[5] Tuma J., Connors W.H., Stitelman D.H., Richert C. (2002). On the Effect of Covalently Appended Quinolones on Termini of DNA Duplexes. J. Am. Chem. Soc..

[6] Pham T.D.M., Ziora Z.M., Blaskovich M.A.T. (2019). Quinolone Antibiotics. Medchemcomm.

[7] King D.E., Malone R., Lilley S.H. (2000). New Classification and Update on the Quinolone Antibiotics. Am. Fam. Physician.

[8] Shams W.E., Evans M.E. (2012). Guide to Selection of Fluoroquinolones in Patients with Lower Respiratory Tract Infections. Drugs.

[9] Turel I. (2002). The Interactions of Metal Ions with Quinolone Antibacterial Agents. Coord. Chem. Rev..

[10] Duffy C.P., Elliott C.J., O’Connor R.A., Heenan M.M., Coyle S., Cleary I.M., Kavanagh K., Verhaegen S., O’Loughlin C.M., NicAmhlaoibh R. (1998). Enhancement of Chemotherapeutic Drug Toxicity to Human Tumour Cells In Vitro by a Subset of Non-Steroidal Anti-Inflammatory Drugs (NSAIDs). Eur. J. Cancer.

[11] Uivarosi V. (2013). Metal Complexes of Quinolone Antibiotics and Their Applications: An Update. Molecules.

[12] Ferreira M., Gameiro P., Ruiz J., Pons M.J. (2021). Fluoroquinolone-Transition Metal Complexes: A Strategy to Overcome Bacterial Resistance. Microorganisms.

[13] Psomas G., Kessissoglou D.P. (2013). Quinolones and Non-Steroidal Anti-Inflammatory Drugs Interacting with Copper(II), Nickel(II), Cobalt(II) and Zinc(II): Structural Features, Biological Evaluation and Perspectives. Dalton Trans..

[14] Irgi E.P., Geromichalos G.D., Balala S., Kljun J., Kalogiannis S., Papadopoulos A., Turel I., Psomas G. (2015). Cobalt(II) Complexes with the Quinolone Antimicrobial Drug Oxolinic Acid: Structure and Biological Perspectives. RSC Adv..

[15] Tarushi A., Psomas G., Raptopoulou C.P., Kessissoglou D.P. (2009). Zinc Complexes of the Antibacterial Drug Oxolinic Acid: Structure and DNA-Binding Properties. J. Inorg. Biochem..

[16] Zampakou M., Akrivou M., Andreadou E.G., Raptopoulou C.P., Psycharis V., Pantazaki A.A., Psomas G. (2013). Structure, Antimicrobial Activity, DNA- and Albumin-Binding of Manganese(II) Complexes with the Quinolone Antimicrobial Agents Oxolinic Acid and Enrofloxacin. J. Inorg. Biochem..

[17] Chalkidou E., Perdih F., Turel I., Kessissoglou D.P., Psomas G. (2012). Copper(II) Complexes with Antimicrobial Drug Flumequine: Structure and Biological Evaluation. J. Inorg. Biochem..

[18] Tsitsa I., Tarushi A., Doukoume P., Perdih F., De Almeida A., Papadopoulos A., Kalogiannis S., Casini A., Turel I., Psomas G. (2016). Structure and Biological Activities of Metal Complexes of Flumequine. RSC Adv..

[19] Skyrianou K.C., Perdih F., Turel I., Kessissoglou D.P., Psomas G. (2010). Nickel-Quinolones Interaction. Part 3—Nickel(II) Complexes of the Antibacterial Drug Flumequine. J. Inorg. Biochem..

[20] Tarushi A., Kljun J., Turel I., Pantazaki A.A., Psomas G., Kessissoglou D.P. (2013). Zinc(II) Complexes with the Quinolone Antibacterial Drug Flumequine: Structure, DNA- and Albumin-Binding. New J. Chem..

[21] Tarushi A., Lafazanis K., Kljun J., Turel I., Pantazaki A.A., Psomas G., Kessissoglou D.P. (2013). First- and Second-Generation Quinolone Antibacterial Drugs Interacting with Zinc(II): Structure and Biological Perspectives. J. Inorg. Biochem..

[22] Nfor E.N., Burrows A.D., Keenan L.L. (2014). A Zinc(II) Coordination Polymer Containing Flumequine: Synthesis, Crystal Structure and Luminescence Properties. Inorg. Chem. Commun..

[23] Arnaouti E., Georgiadou C., Hatzidimitriou A.G., Kalogiannis S., Psomas G. (2024). Erbium(III) Complexes with Fluoroquinolones: Structure and Biological Properties. J. Inorg. Biochem..

[24] Sha J.Q., Li X., Qiu H.B., Zhang Y.H., Yan H. (2012). Nickel Complexes of the Different Quinolone Antibacterial Drugs: Synthesis, Structure and Interaction with DNA. Inorganica Chim. Acta.

[25] Huang J., Hu W.P., An Z. (2008). Poly[[Bis-[Μ2-8-Ethyl-5-Oxo-2-(Piperazin-1-Yl)-5, 8-Dihydro-Pyrido [2,3-d]Pyrimidine-6-Carboxyl-Ato]Manganese(II)] Dihydrate]. Acta Crystallogr. Sect. E Struct. Rep. Online.

[26] Qi X., Shao M., Li C.X. (2009). Poly[[Bis-[Μ2-8-Ethyl-5-Oxo-2-(Piperazin-1-Yl)-5, 8-Dihydro-Pyrido [2,3-d]Pyrimidine-6-Carboxyl-Ato]Cobalt(II)] Dihydrate]. Acta Crystallogr. Sect. E Struct. Rep. Online.

[27] Kljun J., Bratsos I., Alessio E., Psomas G., Repnik U., Butinar M., Turk B., Turel I. (2013). New Uses for Old Drugs: Attempts to Convert Quinolone Antibacterials into Potential Anticancer Agents Containing Ruthenium. Inorg. Chem..

[28] Ruiz M., Ortiz R., Perello L., Latorre J., Server-Carrio J. (1997). Potentiometric and Spectroscopic Studies of Transition-Metal Ions Complexes with a Quinolone Derivative (Cinoxacin). Crystal Structures of New Cu(II) and Ni(II) Cinoxacin Complexes. J. Inorg. Biochem..

[29] Chulvi C., Munoz M.C., Perello L., Ortiz R., Arriortua M.I., Via J., Urtiaga K., Amigo J.M., Ochando L.E. (1991). Coordination Behavior of Cinoxacine: Synthesis and Crystal Structure of Tris(Cinoxacinate)Cobaltate(II) of Sodium Hexahydrate (HCx = 1-Ethyl-4(1H)-Oxo-(1,3)Dioxolo-(4,5g)Cinnoline-3-Carboxylic Acid). J. Inorg. Biochem..

[30] Ruiz M., Perello L., Server-Carrio J., Ortiz R., Garcia-Granda S., Diaz M.R., Canton E. (1998). Cinoxacin Complexes with Divalent Metal Ions. Spectroscopic Characterization. Crystal Structure of a New Dinuclear Cd(II) Complex Having Two Chelate-Bridging Carboxylate Groups. Antibacterial Studies. J. Inorg. Biochem..

[31] Lopez-Gresa M.P., Ortiz R., Perello L., Latorre J., Liu-Gonzalez M., García-Granda S., Perez-Priede M., Canton E. (2002). Interactions of Metal Ions with Two Quinolone Antimicrobial Agents (Cinoxacin and Ciprofloxacin): Spectroscopic and X-Ray Structural Characterization. Antibacterial Studies. J. Inorg. Biochem..

[32] Kljun J., Bytzek A.K., Kandioller W., Bartel C., Jakupec M.A., Hartinger C.G., Keppler B.K., Turel I. (2011). Physicochemical Studies and Anticancer Potency of Ruthenium H6-p-Cymene Complexes Containing Antibacterial Quinolones. Organometallics.

[33] Ruiz M., Perello L., Ortiz R., Castineiras A., Maichle-Mossmer C., Canton E. (1995). Synthesis, Characterization, and Crystal Structure of [Cu(Cinoxacinate)_2_]·2H_2_O Complex: A Square-Planar CuO_4_ Chromophore. Antibacterial Studies. J. Inorg. Biochem..

[34] Bivian-Castro E.Y., Cervantes-Lee F., Mendoza-Díaz G. (2004). Synthesis, Characterization and Crystal Structure of Copper(II) Ternary Complex with Cinoxacin and Histamine. Inorganica Chim. Acta.

[35] Zoroddu M.A., Aaseth J., Crisponi G., Medici S., Peana M., Nurchi V.M. (2019). The Essential Metals for Humans: A Brief Overview. J. Inorg. Biochem..

[36] Yamada K. (2013). Cobalt: Its Role in Health and Disease. Metal Ions in Life Sciences.

[37] Dwyer F.P., Gyarfas E.C., Rogers W.P., Koch J.H. (1952). Biological Activity of Complex Ions. Nature.

[38] Schwartz J.A., Lium E.K., Silverstein S.J. (2001). Herpes Simplex Virus Type 1 Entry Is Inhibited by the Cobalt Chelate Complex CTC-96. J. Virol..

[39] Farrer N.J., Sadler P.J. (2011). Medicinal Inorganic Chemistry: State of the Art, New Trends, and a Vision of the Future. Bioinorganic Medicinal Chemistry.

[40] Anthony E.J., Bolitho E.M., Bridgewater H.E., Carter O.W.L., Donnelly J.M., Imberti C., Lant E.C., Lermyte F., Needham R.J., Palau M. (2020). Metallodrugs Are Unique: Opportunities and Challenges of Discovery and Development. Chem. Sci..

[41] O’Hara J.A., Douple E.B., Abrams M.J., Picker D.J., Giandomenico C.M., Vollano J.F. (1989). Potentiation of Radiation-Induced Cell Kill by Synthetic Metalloporphyrins. Int. J. Radiat. Oncol. Biol. Phys..

[42] Shreaz S., Sheikh R.A., Bhatia R., Neelofar K., Imran S., Hashmi A.A., Manzoor N., Basir S.F., Khan L.A. (2011). Antifungal Activity of α-Methyl Trans Cinnamaldehyde, Its Ligand and Metal Complexes: Promising Growth and Ergosterol Inhibitors. BioMetals.

[43] Patil M., Hunoor R., Gudasi K. (2010). Transition Metal Complexes of a New Hexadentate Macroacyclic N_2_O_4_-Donor Schiff Base: Inhibitory Activity against Bacteria and Fungi. Eur. J. Med. Chem..

[44] Dhanaraj C.J., Johnson J. (2015). Quinoxaline Based Bio-Active Mixed Ligand Transition Metal Complexes: Synthesis, Characterization, Electrochemical, Antimicrobial, DNA Binding, Cleavage, Antioxidant and Molecular Docking Studies. J. Photochem. Photobiol. B.

[45] Tabrizi L., McArdle P., Erxleben A., Chiniforoshan H. (2015). Nickel(II) and Cobalt(II) Complexes of Lidocaine: Synthesis, Structure and Comparative In Vitro Evaluations of Biological Perspectives. Eur. J. Med. Chem..

[46] Prabhakara C.T., Patil S.A., Kulkarni A.D., Naik V.H., Manjunatha M., Kinnal S.M., Badami P.S. (2015). Synthesis, Spectral, Thermal, Fluorescence, Antimicrobial, Anthelmintic and DNA Cleavage Studies of Mononuclear Metal Chelates of Bi-Dentate 2H-Chromene-2-One Schiff Base. J. Photochem. Photobiol. B.

[47] Sherif Y.E., Hosny N.M. (2014). Anti-Rheumatic Potential of Ethyl 2-(2-Cyano-3-Mercapto-3-(Phenylamino) Acrylamido)-4,5,6,7-Tetrahydrobenzo[b]Thiophene-3-Carboxylate and Its Co(II), Cu(II) and Zn(II) Complexes. Eur. J. Med. Chem..

[48] Netalkar P.P., Netalkar S.P., Budagumpi S., Revankar V.K. (2014). Synthesis, Crystal Structures and Characterization of Late First Row Transition Metal Complexes Derived from Benzothiazole Core: Anti-Tuberculosis Activity and Special Emphasis on DNA Binding and Cleavage Property. Eur. J. Med. Chem..

[49] Galal S.A., Abd El-All A.S., Hegab K.H., Magd-El-Din A.A., Youssef N.S., El-Diwani H.I. (2010). Novel Antiviral Benzofuran-Transition Metal Complexes. Eur. J. Med. Chem..

[50] Pires B.M., Giacomin L.C., Castro F.A.V., dos S. Cavalcanti A., Pereira M.D., Bortoluzzi A.J., Faria R.B., Scarpellini M. (2016). Azido- and Chlorido-Cobalt Complex as Carrier-Prototypes for Antitumoral Prodrugs. J. Inorg. Biochem..

[51] Eshkourfu R., Cobeljic B., Vujcic M., Turel I., Pevec A., Sepcic K., Zec M., Radulovic S., Srdic-Radic T., Mitic D. (2011). Synthesis, Characterization, Cytotoxic Activity and DNA Binding Properties of the Novel Dinuclear Cobalt(III) Complex with the Condensation Product of 2-Acetylpyridine and Malonic Acid Dihydrazide. J. Inorg. Biochem..

[52] Jimenez-Garrido N., Perello L., Ortiz R., Alzuet G., Gonzalez-Alvarez M., Cantan E., Liu-Gonzalez M., Garcia-Granda S., Perez-Priede M. (2005). Antibacterial Studies, DNA Oxidative Cleavage, and Crystal Structures of Cu(II) and Co(II) Complexes with Two Quinolone Family Members, Ciprofloxacin and Enoxacin. J. Inorg. Biochem..

[53] Protogeraki C., Andreadou E.G., Perdih F., Turel I., Pantazaki A.A., Psomas G. (2014). Cobalt(II) Complexes with the Antimicrobial Drug Enrofloxacin: Structure, Antimicrobial Activity, DNA- and Albumin-Binding. Eur. J. Med. Chem..

[54] He J.H., Xiao D.R., Chen H.Y., Sun D.Z., Yan S.W., Wang X., Ye Z.L., Luo Q.L., Wang E.B. (2013). A Series of 2D Metal–Quinolone Complexes: Syntheses, Structures, and Physical Properties. J. Solid. State Chem..

[55] Kouris E., Kalogiannis S., Perdih F., Turel I., Psomas G. (2016). Cobalt(II) Complexes of Sparfloxacin: Characterization, Structure, Antimicrobial Activity and Interaction with DNA and Albumins. J. Inorg. Biochem..

[56] Janzen L., Miller R.G., Metzler-Nolte N. (2024). Synthesis, Characterisation and Antimicrobial Activity of Supramolecular Cobalt-Peptide Conjugates. Dalton Trans..

[57] Chang E.L., Simmers C., Knight D.A. (2010). Cobalt Complexes as Antiviral and Antibacterial Agents. Pharmaceuticals.

[58] Takeuchi T., Böttcher A., Quezada C.M., Meade T.J., Gray H.B. (1999). Inhibition of Thermolysin and Human α-Thrombin by Cobalt(III) Schiff Base Complexes. Bioorg Med. Chem..

[59] Louie A.Y., Meade T.J. (1998). A Cobalt Complex That Selectively Disrupts the Structure and Function of Zinc Fingers. Proc. Natl. Acad. Sci. USA.

[60] Blower P.J., Dilworth J.R., Maurer R.I., Mullen G.D., Reynolds C.A., Zheng Y. (2001). Towards New Transition Metal-Based Hypoxic Selective Agents for Therapy and Imaging. J. Inorg. Biochem..

[61] Kanina A., Mei H., Palma C., Neary M.C., Cheng S.-Y., Zhang G. (2025). Synthesis, Reductive Reactivity and Anticancer Activity of Cobalt(III)– and Manganese(III)–Salen Complexes. Chemistry.

[62] Law B.Y.K., Qu Y.Q., Mok S.W.F., Liu H., Zeng W., Han Y., Gordillo-Martinez F., Chan W.-K., Wong K.M.-C., Wong V.K.W. (2017). New Perspectives of Cobalt Tris(Bipyridine) System: Anti-Cancer Effect and Its Collateral Sensitivity towards Multidrug-Resistant (MDR) Cancers. Oncotarget.

[63] Wilson W.R., Moselen J.W., Cliffe S., Denny W.A., Ware D.C. (1994). Exploiting Tumor Hypoxia Through Bioreductive Release of Diffusible Cytotoxins: The Cobalt(III)-Nitrogen Mustard Complex SN 24771. Int. J. Radiat. Oncol. Biol. Phys..

[64] Allardyce C.S., Dyson P.J. (2016). Metal-Based Drugs That Break the Rules. Dalton Trans..

[65] Rubin-Preminger J.M., Kozlov L., Goldberg I. (2008). Hydrogen-Bonding and π–π Stacking Interactions in Aquachloridobis(1,10-Phenanthroline)Cobalt(II) Chloride Dichloridobis(1,10-Phenanthroline)Cobalt(II) Hexahydrate. Acta Crystallogr. C.

[66] Vlcek A.A. (1967). Preparation of Co(Dipy)_2_X_2_^+^ Complexes (X^−^ = Chloride, Bromide, Iodide, Nitrite) by Controlled Oxidative Processes. Inorg. Chem..

[67] McKenzie E.D. (1971). The Steric Effect in Bis(2,2′-Bipyridyl) and Bis(1,10-Phenanthroline) Metal Compounds. Coord. Chem. Rev..

[68] Geary W.J. (1971). The Use of Conductivity Measurements in Organic Solvents for the Characterisation of Coordination Compounds. Coord. Chem. Rev..

[69] Nakamoto K. (2008). Infrared and Raman Spectra of Inorganic and Coordination Compounds: Part B: Applications in Coordination, Organometallic, and Bioinorganic Chemistry.

[70] Szorcsik A., Nagy L., Sletten J., Szalontai G., Kamu E., Fiore T., Pellerito L., Kálmán E. (2004). Preparation and Structural Studies on Dibutyltin(IV) Complexes with Pyridine Mono- and Dicarboxylic Acids. J. Organomet. Chem..

[71] Bernhardt P.V., Lawrance G.A. (2003). Cobalt. Comprehensive Coordination Chemistry II.

[72] Kakoulidou C., Kalogiannis S., Angaridis P., Psomas G. (2019). Synthesis, Characterization and Biological Activity of Zn Coordination Compounds with the Quinolone Gatifloxacin. Polyhedron.

[73] Hadjiliadis N.D., Sletten E., Hadjiliadis N., Sletten E. (2009). Metal Complex-DNA Interactions.

[74] Rehman S.U., Sarwar T., Husain M.A., Ishqi H.M., Tabish M. (2015). Studying Non-Covalent Drug–DNA Interactions. Arch. Biochem. Biophys..

[75] Zeglis B.M., Pierre V.C., Barton J.K. (2007). Metallo-Intercalators and Metallo-Insertors. Chem. Commun..

[76] Wolfe A., Shimer G.H., Meehan T. (1987). Polycyclic Aromatic Hydrocarbons Physically Intercalate into Duplex Regions of Denatured DNA. Biochemistry.

[77] Dimitrakopoulou A., Dendrinou-Samara C., Pantazaki A.A., Alexiou M., Nordlander E., Kessissoglou D.P. (2008). Synthesis, Structure and Interactions with DNA of Novel Tetranuclear, [Mn_4_(II/II/II/IV)] Mixed Valence Complexes. J. Inorg. Biochem..

[78] Bravo-Anaya L., Rinaudo M., Martínez F. (2016). Conformation and Rheological Properties of Calf-Thymus DNA in Solution. Polymers.

[79] Arjmand F., Aziz M., Tabassum S. (2010). Cyclic Voltammetry-An Electrochemical Approach to Study Metal-Based Potential Antitumor Drug-DNA Interaction. Curr. Anal. Chem..

[80] Arshad N., Farooqi S.I. (2018). Cyclic Voltammetric DNA Binding Investigations on Some Anticancer Potential Metal Complexes: A Review. Appl. Biochem. Biotechnol..

[81] Zivec P., Perdih F., Turel I., Giester G., Psomas G. (2012). Different Types of Copper Complexes with the Quinolone Antimicrobial Drugs Ofloxacin and Norfloxacin: Structure, DNA- and Albumin-Binding. J. Inorg. Biochem..

[82] Carter M.T., Rodriguez M., Bard A.J. (1989). Voltammetric Studies of the Interaction of Metal Chelates with DNA. 2. Tris-Chelated Complexes of Cobalt(III) and Iron(II) with 1,10-Phenanthroline and 2,2′-Bipyridine. J. Am. Chem. Soc..

[83] Garbett N.C., Hammond N.B., Graves D.E. (2004). Influence of the Amino Substituents in the Interaction of Ethidium Bromide with DNA. Biophys. J..

[84] Tsai C.C., Jain S.C., Sobell H.M. (1977). Visualization of Drug-Nucleic Acid Interactions at Atomic Resolution: I. Structure of an Ethidium/Dinucleoside Monophosphate Crystalline Complex, Ethidium:5-Iodouridylyl (3′–5′) Adenosine. J. Mol. Biol..

[85] Wilson W.D., Ratmeyer L., Zhao M., Strekowski L., Boykin D. (1993). The Search for Structure-Specific Nucleic Acid-Interactive Drugs: Effects of Compound Structure on RNA versus DNA Interaction Strength. Biochemistry.

[86] Lakowicz J.R. (2006). Principles of Fluorescence Spectroscopy.

[87] Heller D.P., Greenstock C.L. (1994). Fluorescence Lifetime Analysis of DNA Intercalated Ethidium Bromide and Quenching by Free Dye. Biophys. Chem..

[88] He X.M., Carter D.C. (1992). Atomic Structure and Chemistry of Human Serum Albumin. Nature.

[89] Olson R.E., Christ D.D. (1996). Chapter 33. Plasma Protein Binding of Drugs. Annu. Rep. Med. Chem..

[90] Stella L., Capodilupo A.L., Bietti M. (2008). A Reassessment of the Association between Azulene and [60]Fullerene. Possible Pitfalls in the Determination of Binding Constants through Fluorescence Spectroscopy. Chem. Commun..

[91] Laitinen O.H., Hytonen V.P., Nordlund H.R., Kulomaa M.S. (2006). Genetically Engineered Avidins and Streptavidins. Cell. Mol. Life Sci..

[92] Strenger I., Rosu T., Negoiu M. (2000). Refinement of the Crystal Structure of Cw-Bis(2,2′-Bipyridyl)-Dichlorocobalt(III) Chloride Dihydrate, [C_20_H_16_N_4_CoCl_2_]Cl·2H_2_O. Z. Für Krist.—New Cryst. Struct..

[93] Marmur J. (1961). A Procedure for the Isolation of Deoxyribonucleic Acid from Micro-Organisms. J. Mol. Biol..

[94] Reichmann M.E., Rice S.A., Thomas C.A., Doty P. (1954). A Further Examination of the Molecular Weight and Size of Desoxypentose Nucleic Acid. J. Am. Chem. Soc..

[95] Bruker Analytical X-Ray Systems, Inc. (2006). Apex2, Version 2 User Manual.

[96] Siemens Industrial Automation, Inc. (1996). SADABS: Area–Detector Absorption Correction.

[97] Betteridge P.W., Carruthers J.R., Cooper R.I., Prout K., Watkin D.J. (2003). CRYSTALS Version 12: Software for Guided Crystal Structure Analysis. J. Appl. Crystallogr..

[98] Palatinus L., Chapuis G. (2007). SUPERFLIP—A Computer Program for the Solution of Crystal Structures by Charge Flipping in Arbitrary Dimensions. J. Appl. Crystallogr..

[99] Wang Y.-Q., Zhang H.-M., Zhang G.-C., Tao W.-H., Tang S.-H. (2007). Interaction of the Flavonoid Hesperidin with Bovine Serum Albumin: A Fluorescence Quenching Study. J. Lumin..

[100] de Meulenaer J., Tompa H. (1965). The Absorption Correction in Crystal Structure Analysis. Acta Crystallogr..

