# Supplementary material for: Synthesis, Characterization and Biological Profile of Cationic Cobalt Complexes with First-Generation Quinolones
Source: Molecules. 2025 Jun 19;30(12):2646. doi: 10.3390/molecules30122646 (PMC12196173; doi:10.3390/molecules30122646)
Supplement: Supplementary file 1 [file molecules-30-02646-s001.zip › molecules-3700234-ESI.pdf]

Synthesis, Characterization and Biological Profile of Cationic Cobalt Complexes with First-Generation Quinolones

Alexia Tialiou, Antonios G. Hatzidimitriou and George Psomas *

Laboratory of Inorganic Chemistry, Department of Chemistry, Aristotle University of Thessaloniki, GR-54124 Thessaloniki, Greece

* Correspondence: gepsomas@chem.auth.gr

Supplementary material

Contents

EXPERIMENTAL PROTOCOLS 4
S1 Binding studies with CT DNA 4
S1.1 Binding study with CT DNA by UV−vis spectroscopy 4
S1.2 CT DNA−binding studies by viscosity measurements 4
S1.3 Study of the DNA–interaction by cyclic voltammetry 4
S1.4 EB−displacement studies 5
S2 Interaction with serum albumins 5
S3 References 7
TABLES 8
Table S1. Crystallographic data for complexes **1–6** 8
Table S2. Selected bond lengths (Å) and bond angles (°) for complexes **1-6**. 10
Table S3. Hydrogen–bonding interactions (lengths Å in and angles in º) in structures of complexes **1-6** 11
FIGURES 12
Figure S1. IR spectra (KBr) of the complexes. 12
Figure S2. ^{1}H NMR spectra of the complexes in a 95:5 mixture of D_2O : DMSO-d_6. 15
Figure S3. ^{1}H NMR spectra of complex **1** in a 95:5 mixture of D_2O : DMSO-d_6 for two different time intervals (0 h and 24 h). 18
Figure S4. UV−vis spectra of a CT DNA buffer solution, in the presence of increasing amounts of the complexes. 19
Figure S5. UV−vis spectra of a DMSO solution of the complexes, in the presence of increasing amounts of CT DNA 20
Figure S6. Plots of [DNA]$(\varepsilon A - \varepsilon f)$ *versus* [DNA] for the complexes. 21
Figure S7. Cyclic voltammograms of the complexes in the absence (black line) or presence (green line) of CT DNA 22
Figure S8. Fluorescence emission spectra for EB-DNA in buffer solution in the absence and presence of increasing amounts of the compounds. 23
Figure S9. Stern−Volmer plots of the EB−DNA quenching experiments upon addition of the compounds. 24
Figure S10. Fluorescence emission spectra of BSA in buffer solution in the presence of increasing amounts of the compounds. 25

Figure S11. Fluorescence emission spectra of HSA in buffer solution in the presence of increasing amounts of the compounds. 26

Figure S12. Stern–Volmer plots of the BSA-quenching experiments upon addition of the compounds. 27

Figure S13. Stern–Volmer plots of the HSA-quenching experiments upon addition of the compounds. 28

Figure S14. Scatchard plots of the BSA-quenching experiments upon addition of the compounds. 29

Figure S15. Scatchard plots of the HSA-quenching experiments upon addition of the compounds. 30

EXPERIMENTAL PROTOCOLS

S1 Binding studies with CT DNA

The interaction of the compounds with CT–DNA was investigated by UV–vis spectroscopy, viscosity measurements, cyclic voltammetry, and fluorescence emission spectroscopy studies.

S1.1 Binding study with CT DNA by UV−vis spectroscopy

UV–vis spectroscopy was used for the evaluation of the interaction of the compounds with CT DNA, and specifically the possible binding modes of the compounds to CT DNA. Control experiments with DMSO were performed and no changes in the spectra of CT DNA were observed.

In order to determine the binding mode, the UV–vis spectra of the compounds were recorded for a constant concentration (3×10^{-5} – 5×10^{-5} M) at the corresponding λ_{max} with increasing concentrations of CT DNA for diverse r values (r = [complex]/[DNA]). Effective use of the changes in the absorbance of the UV–vis spectra was made and the DNA–binding constants (K_b, in M^{-1}) of the compounds were calculated by the Wolfe–Shimer equation (equation S1)) [1] and the plots [DNA]/(ε_A–ε_f) *versus* [DNA]:

$$\frac{[\mathrm{DNA}]}{(\varepsilon_A-\varepsilon_f)}=\frac{[\mathrm{DNA}]}{(\varepsilon_b-\varepsilon_f)}+\frac{1}{K_b(\varepsilon_b-\varepsilon_f)} \qquad \text{(equation S1)}$$

where [DNA] = the concentration of DNA in base pairs, ε_f= the extinction coefficient for the free compound at the corresponding λ_{max}, $\varepsilon_A = A_{obsd}$/[compound] and ε_b= the extinction coefficient for the compound in the fully bound form. K_b is given by the ratio of slope to the y intercept in plots [DNA]/(ε_A–ε_f) *versus* [DNA].

S1.2 CT DNA–binding studies by viscosity measurements

The viscosity of DNA (0.1 mM) in buffer solution was measured in the absence and presence of increasing amounts of the compounds. The experiments were executed at room temperature and the measurements are devised in a plot $(\eta/\eta_0)^{1/3}$ *versus* r (r = [complex]/[DNA]), where η = the viscosity of DNA in the presence of the compound, and η_0 = the viscosity of neat DNA in buffer solution.

S1.3 Study of the DNA–interaction by cyclic voltammetry

Cyclic voltammetry can be also used in order to calculate the corresponding equilibrium constant for the redox process. The oxidized and reduced forms are associated with a third species (DNA) in the solution using the following equation [2] :

$$\Delta E^o = E^o_{(b)} - E^o_{(f)} = 0.059 \times \log \frac{K_r}{K_{ox}} \qquad \text{(equation S2)}$$

where $E^o_{(b)}$ and $E^o_{(f)}$ are the formal potentials of $M^{(oxidized)}/M^{(reduced)}$ couple in the fully bound and free complexes, respectively. K_{ox} and K_r are the binding constants for the binding of the oxidized and reduced species to DNA, respectively.

S1.4 EB–displacement studies

In order to determine and confirm the DNA–binding mode of the compounds, a competitive study with EB as an intercalating marker is performed by fluorescence emission spectroscopy. Therefore, the EB–displacing ability of the compounds from its EB–DNA conjugate was examined. The DNA–EB adduct was prepared by addition of 20 μM EB and 26 μM CT DNA in buffer solution (150 mM NaCl and 15 mM trisodium citrate at pH 7.0). The potential intercalation of the compounds between the DNA–bases was studied by the addition of a certain amount of the compound solution into the EB–DNA adduct solution. The influence of the compounds on the EB–DNA solution was monitored through the changes of the fluorescence emission spectra at excitation wavelength (λ_{ex}) at 540 nm) [3]. The tested compounds do not show any significant fluorescence at room temperature in solution or in the presence of DNA, under the same experimental conditions (λ_{ex} = 540 nm). Bearing that in mind, the observed quenching of the EB–DNA solution is evidently associated to the displacement of EB from its EB–DNA adduct. The quenching efficiency (K_{SV}, in M^{-1}) for each compound was assessed according to the Stern–Volmer equation (equation S3)) [3]:

$$\frac{Io}{I} = 1 + k_q \tau_0 [Q] = 1 + K_{SV}[Q] \qquad \text{(equation S3)}$$

where I_0 and I = the fluorescence emission intensities of EB–DNA in the absence and presence of the quencher, respectively, [Q] = the concentration of the quencher (i.e. compounds). K_{SV} is obtained from the Stern–Volmer plots by the slope of the diagram I_0/I *versus* [Q]. Taking τ_0 = 23 ns as the fluorescence lifetime of the EB–DNA system [4], the EB–DNA quenching constants (k_q, in $M^{-1}s^{-1}$) of the compounds can be determined according to equation S4:

$$K_{SV} = k_q \tau_o \qquad \text{(equation S4)}$$

S2 Interaction with serum albumins

The albumin–binding study for the compounds was carried out by fluorescence emission quenching experiments using BSA (3 μM) or HSA (3 μM) in buffer solution (15 mM trisodium citrate and 150 mM NaCl at pH 7.0). The tested compounds were used as quenchers with gradually increasing concentrations to monitor the quenching of the

emission intensity of tryptophan residues of the albumin [3]. The fluorescence emission spectra were recorded between 300–500 nm with excitation wavelength of 295 nm. All the experiments were conducted at room temperature. The fluorescence spectra of the compounds were recorded under the same experimental conditions and some of them presented a low–intensity emission band in the region 370–415 nm. Consequently, the BSA–fluorescence emission spectra were modified properly, by subtracting the spectra of the compounds, and quantitative studies followed.

The extent of the inner–filter effect can be roughly estimated with the following formula:

$$I_{corr} = I_{meas} \times 10^{\frac{\varepsilon(\lambda_{exc})cd}{2}} \times 10^{\frac{\varepsilon(\lambda_{em})cd}{2}} \qquad \text{(equation S5)}$$

where I_{corr} = corrected intensity, I_{meas} = the measured intensity, c = the concentration of the quencher, d = the cuvette length (1 cm), $\varepsilon(\lambda_{exc})$ and $\varepsilon(\lambda_{em})$ = the ε of the quencher at the excitation and the emission wavelength, respectively, as calculated from the UV–vis spectra of the complexes [5] .

The interaction of the quencher (i.e. compounds) with serum albumins was studied through the Stern–Volmer and Scatchard equations [3] and corresponding graphs. The values of the respective Stern–Volmer constant K_{SV} (in M^{-1}), the quenching constant k_q (in $M^{-1}s^{-1}$), the albumin–binding constant K (in M^{-1}) and the number of binding sites per albumin (n) were calculated.

According to Stern–Volmer quenching equation (equation S3) [3], where I_0 = the initial tryptophan fluorescence intensity of the albumin, I = the tryptophan fluorescence intensity of the albumin after the addition of the quencher, k_q = the quenching rate constants of the albumin, K_{SV} = the dynamic quenching constant, τ_0 = the average lifetime of the albumin without the quencher, [Q] = the concentration of the quencher), the Stern–Volmer constant (K_{SV}, M^{-1}) can be obtained by the slope of the diagram I_0/I *versus* [Q]. Taking $\tau_0 = 10^{-8}$ s as fluorescence lifetime of tryptophan in the albumin [3], the quenching constant (k_q, $M^{-1}s^{-1}$) is calculated from equation S4.

From the Scatchard equation (equation S6) [3]:

$$\frac{\Delta I/I_0}{[Q]} = nK - K\frac{\Delta I}{I_0} \qquad \text{(equation S6)}$$

where n = the number of binding sites per albumin and K = the albumin–binding constant. The K constant (M^{-1}) is calculated from the slope in plots $(\Delta I/I_0)/[Q]$ *versus* $(\Delta I/I_0)$ and n is given by the ratio of y intercept to the slope [6].

S3 References

[1] A. Wolfe, G.H. Shimer, T. Meehan, Polycyclic Aromatic Hydrocarbons Physically Intercalate into Duplex Regions of Denatured DNA, Biochemistry 26 (1987) 6392–6396. https://doi.org/10.1021/bi00394a013.

[2] M.T. Carter, M. Rodriguez, A.J. Bard, Voltammetric studies of the interaction of metal chelates with DNA. 2. Tris-chelated complexes of cobalt(III) and iron(II) with 1,10-phenanthroline and 2,2'-bipyridine, J Am Chem Soc 111 (1989) 8901–8911. https://doi.org/10.1021/ja00206a020.

[3] J.R. Lakowicz, Principles of Fluorescence Spectroscopy, Springer US, Boston, MA, 2006. https://doi.org/10.1007/978-0-387-46312-4.

[4] D.P. Heller, C.L. Greenstock, Fluorescence lifetime analysis of DNA intercalated ethidium bromide and quenching by free dye, Biophys Chem 50 (1994) 305–312. https://doi.org/10.1016/0301-4622(93)E0101-A.

[5] L. Stella, A.L. Capodilupo, M. Bietti, A reassessment of the association between azulene and [60]fullerene. Possible pitfalls in the determination of binding constants through fluorescence spectroscopy, Chemical Communications (2008) 4744–4746. https://doi.org/10.1039/b808357f.

[6] Y.-Q. Wang, H.-M. Zhang, G.-C. Zhang, W.-H. Tao, S.-H. Tang, Interaction of the flavonoid hesperidin with bovine serum albumin: A fluorescence quenching study, J Lumin 126 (2007) 211–218. https://doi.org/10.1016/J.JLUMIN.2006.06.013.

[7] J. de Meulenaer, H. Tompa, The absorption correction in crystal structure analysis, Acta Crystallogr 19 (1965) 1014–1018. https://doi.org/10.1107/S0365110X65004802.

TABLES

Table S1. Crystallographic data for complexes **1–6**.

	Complex **1**	Complex **2**	Complex **3**	Complex **4**	Complex **5**	Complex **6**
**Crystal data**						
Chemical formula	$C_{33}H_{28}CoF_{12}N_5O_6P_2$	$C_{37.50}H_{29}Co_1F_{12}N_5O_6P_2$	$C_{34.50}H_{30}Co_1F_{13}N_5O_4P_2$	$C_{35}H_{38}CoF_{12}N_9O_5P_2$	$C_{37}H_{30}Co_1F_{12}N_6O_{6.50}P_2$	$C_{38.50}H_{31}Co_1F_7N_5O_{4.50}P$
M_r	939.47	994.53	946.50	1013.60	1011.54	858.59
Crystal system	Triclinic	Monoclinic	Monoclinic	Triclinic	Monoclinic	Monoclinic
Space group	*P*-1	$P2_1/c$	$P2_1/c$	*P*-1	$P2_1/a$	$P2_1/c$
Temperature (K)	295	295	295	294	295	295
a (Å)	10.923 (2)	15.7047 (16)	16.218 (2)	10.3630 (15)	14.5285 (17)	17.840 (3)
b (Å)	12.278 (3)	18.2941 (17)	16.218 (2)	16.4879 (19)	18.108 (2)	13.639 (2)
c (Å)	14.169 (4)	14.4680 (14)	17.274 (2)	16.672 (2)	15.4842 (16)	17.109 (3)
α (°)	84.505 (13)	90	90	61.688 (6)	90	90
β (°)	86.603 (11)	91.730 (3)	95.715 (4)	87.777 (7)	90.389 (3)	105.694 (4)
γ (°)	78.723 (11)	90	90	84.807 (6)	90	90
V ($Å^3$)	1853.5 (8)	4154.8 (7)	3907.5 (9)	2497.6 (6)	4073.5 (8)	4007.6 (11)
Z	2	4	4	2	4	4
Radiation type	Mo*Kα*	Mo*Kα*	Mo*Kα*	Mo*Kα*	Mo*Kα*	Mo*Kα*
μ (mm^{-1})	0.66	0.60	0.63	0.50	0.61	0.55
Crystal size (mm)	0.20 × 0.17 × 0.15	0.22 × 0.16 × 0.15	0.24 × 0.19 × 0.15	0.22 × 0.18 × 0.11	0.20 × 0.15 × 0.14	0.24 × 0.19 × 0.16
**Data collection**						
Diffractometer	Bruker Kappa Apex2					
Absorption correction	Numerical, Analytical Absorption [7]					
T_{min}, T_{max}	0.89, 0.91	0.91, 0.91	0.89, 0.91	0.91, 0.95	0.91, 0.92	0.90, 0.92

Measured reflections	47210	47349	50578	44782	28482	40455
Independent reflections	7076	7913	7446	9554	7705	7598
Observed [$I > 2.0\sigma(I)$] reflections	5406	5146	5688	6905	5801	5450
R_{int}	0.033	0.021	0.029	0.022	0.015	0.025
$(\sin \theta/\lambda)_{max}$ (Å^{-1})	0.613	0.612	0.611	0.615	0.615	0.614
**Refinement**						
$R[F^2 > 2\sigma(F^2)]$	0.051	0.053	0.052	0.057	0.060	0.055
$wR(F^2)$	0.080	0.105	0.094	0.115	0.113	0.097
S	1.00	1.00	1.00	1.00	1.00	1.00
Reflections	5406	5146	5688	6905	5801	5450
Parameters	532	559	525	617	575	508
Restraints	-	42	13	22	12	8
H-atom treatment	H-atom parameters constrained	H-atom parameters constrained	H-atom parameters constrained	H atoms treated by a mixture of independent and constrained refinement	H-atom parameters constrained	H-atom parameters constrained
$\Delta\rho_{max}$, $\Delta\rho_{min}$ (e Å^{-3})	0.57, −0.30	0.63, −0.48	0.48, −0.37	0.52, −0.28	0.50, −0.96	0.39, −0.24

Table S2. Selected bond lengths (Å) and bond angles (°) for complexes **1-6**.

**Compound**	Complex **1**	Complex **2**	Complex **3**	Complex **4**	Complex **5**	Complex **6**
**Bond**	**Length (Å)**	**Length (Å)**	**Length (Å)**	**Length (Å)**	**Length (Å)**	**Length (Å)**
Co1—O1	1.879 (2)	1.889 (3)	1.893 (2)	1.891 (2)	1.901 (2)	1.923 (2)
Co1—O3	1.881 (2)	1.879 (2)	1.9055 (19)	1.878 (2)	1.892 (2)	1.9235 (19)
Co1—N1	1.935 (2)	1.927 (3)	1.962 (3)	1.943 (3)	1.943 (3)	2.042 (3)
Co1—N2	1.937 (3)	1.940 (3)	1.926 (3)	1.939 (3)	1.922 (3)	2.018 (2)
Co1—N3	1.934 (2)	1.926 (3)	1.963 (2)	1.928 (3)	1.943 (3)	2.034 (3)
Co1—N4	1.904 (3)	1.940 (3)	1.959 (2)	1.964 (3)	1.924 (3)	2.031 (3)
**Bonds**	**Angle (°)**	**Angle (°)**	**Angle (°)**	**Angle (°)**	**Angle (°)**	**Angle (°)**
O1—Co1—O3	95.28 (9)	94.83 (11)	94.85 (9)	95.09 (10)	94.09 (10)	91.97 (9)
O1—Co1—N1	177.31 (11)	89.28 (12)	174.47 (11)	176.41 (11)	176.56 (12)	174.35 (10)
O3—Co1—N1	86.43 (9)	92.15 (13)	87.91 (10)	86.28 (11)	86.76 (11)	87.29 (9)
O1—Co1—N2	94.63 (11)	87.42 (11)	93.26 (10)	93.06 (10)	93.91 (12)	93.35 (10)
O3—Co1—N2	85.37 (10)	175.92 (13)	89.06 (10)	88.76 (11)	88.05 (11)	92.29 (9)
N1—Co1—N2	83.42 (11)	84.46 (13)	81.97 (12)	83.64 (11)	82.78 (13)	81.09 (11)
O1—Co1—N3	85.58 (10)	93.56 (13)	86.90 (9)	88.33 (11)	87.79 (10)	90.40 (10)
O3—Co1—N3	177.35 (11)	87.97 (12)	175.76 (10)	174.27 (11)	176.29 (13)	173.30 (10)
N1—Co1—N3	92.81 (10)	177.14 (14)	90.68 (10)	90.59 (12)	91.55 (11)	90.96 (10)
N2—Co1—N3	97.07 (11)	95.29 (12)	94.70 (11)	95.66 (12)	95.02 (12)	93.83 (11)
O1—Co1—N4	87.57 (11)	176.82 (13)	89.92 (9)	88.07 (10)	89.74 (11)	93.55 (9)
O3—Co1—N4	94.54 (11)	85.66 (12)	93.45 (10)	92.89 (11)	92.72 (12)	92.71 (10)
N1—Co1—N4	94.38 (11)	93.85 (13)	94.71 (11)	95.19 (11)	93.54 (13)	92.08 (10)
N2—Co1—N4	177.80 (11)	92.28 (12)	175.77 (11)	177.92 (12)	176.20 (13)	171.33 (11)
N3—Co1—N4	82.99 (12)	83.32 (14)	82.68 (11)	82.61 (12)	84.08 (12)	80.88 (12)

Table S3. Hydrogen–bonding interactions (lengths Å in and angles in º) in structures of complexes **1-6**.

D—H···*A*	*D*—H (Å)	H···*A* (Å)	*D*···*A* (Å)	*D*—H···*A* (º)	Symmetry code
**1**					
O6—H61···O2^{i}	0.84	1.98	2.794 (5)	164	(i) −*x*+1, −*y*+1, −*z*
O6—H62···O2^{ii}	0.84	2.57	3.251 (5)	139	(ii) *x*−1, *y*, *z*
**2**					
O6—H61···O2	0.82	2.35	3.000 (7)	136	
O7—H71···O4	0.85	2.22	3.006 (7)	154	
**3**					
O4—H37···O2^{i}	0.82	1.99	2.752 (6)	154	(i) *x*, −*y*+1/2, *z*−1/2
O5—H591···O4	0.82	1.70	2.498 (6)	163	
**4**					
O4—H41···N9	0.83	2.15	2.964 (8)	165	
O4—H41···N10	0.83	1.49	2.168 (8)	136	
O4—H42···O8^{i}	0.88	2.58	3.418 (8)	159	(i) −*x*+1, −*y*+1, −*z*+1
O5—H52···O4	0.83	2.17	2.811 (8)	134	
O7—H71···O2	0.87	2.07	2.674 (8)	125	
N9—H91···O4^{ii}	0.84	2.22	2.756 (8)	122	(ii) −*x*+2, −*y*+1, −*z*+1
N9—H101···O4	1.14	1.84	2.964 (8)	168	
O5—H396···O6	0.83	2.01	2.818 (8)	166	
O8—H397···O7^{v}	0.83	1.92	2.742 (8)	172	(v) *x*, *y*+1, *z*
**5**					
O6—H61···O2	0.85	2.23	3.075 (6)	176	
O7—H72···O4	0.83	2.42	3.220 (6)	161	
**6**					
O5—H52···O2	0.82	2.42	3.050 (6)	134	
O6—H61···O5	0.82	2.39	3.150 (6)	154	
O5—H394···O1	0.82	2.49	3.128 (6)	136	

FIGURES

Figure S1. IR spectra (KBr) of the complexes.

Complex **1**

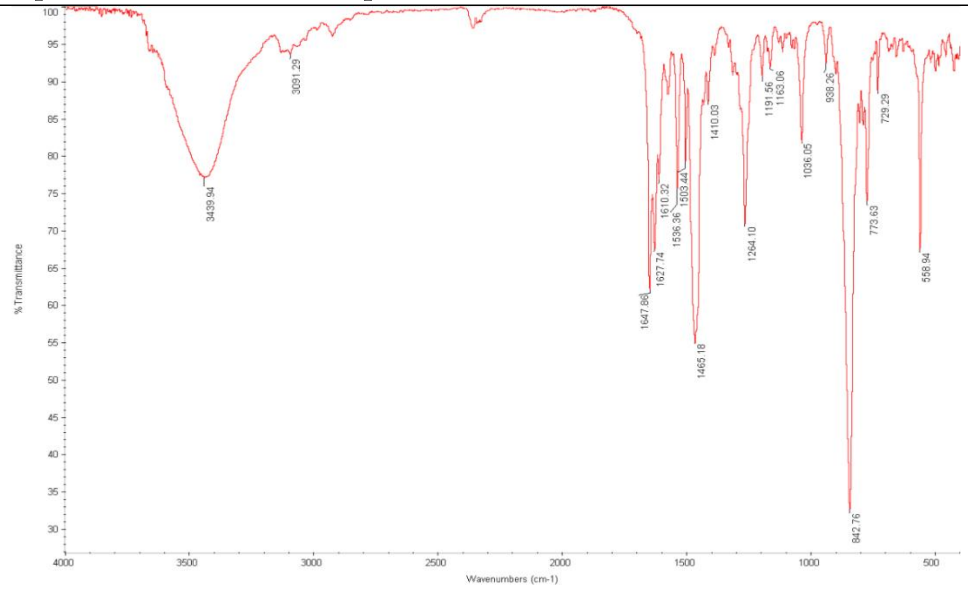

Complex **2**

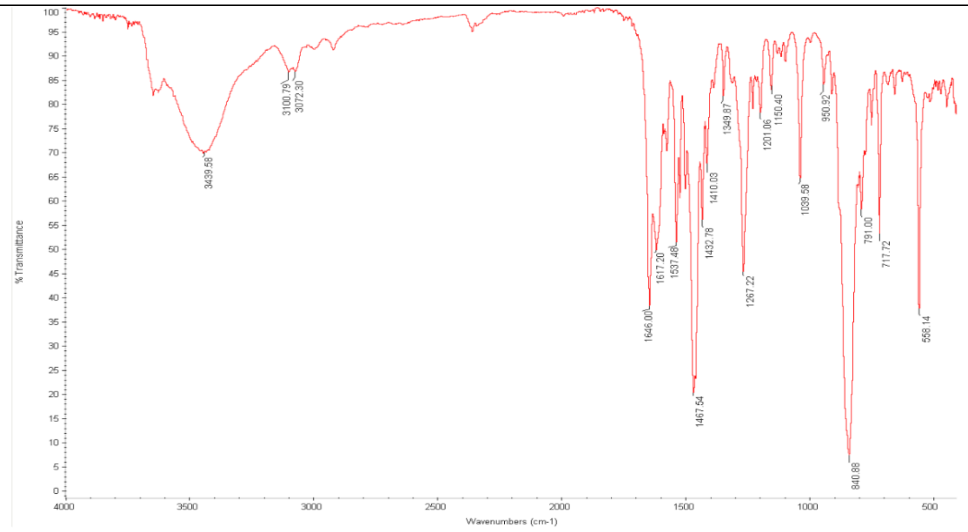

Complex **3**

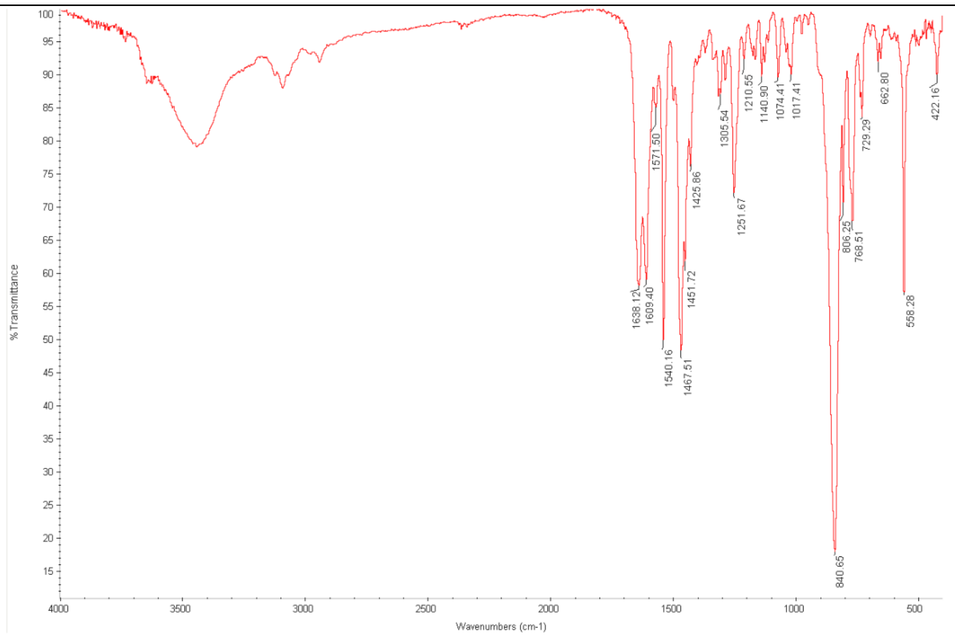

Complex **4**

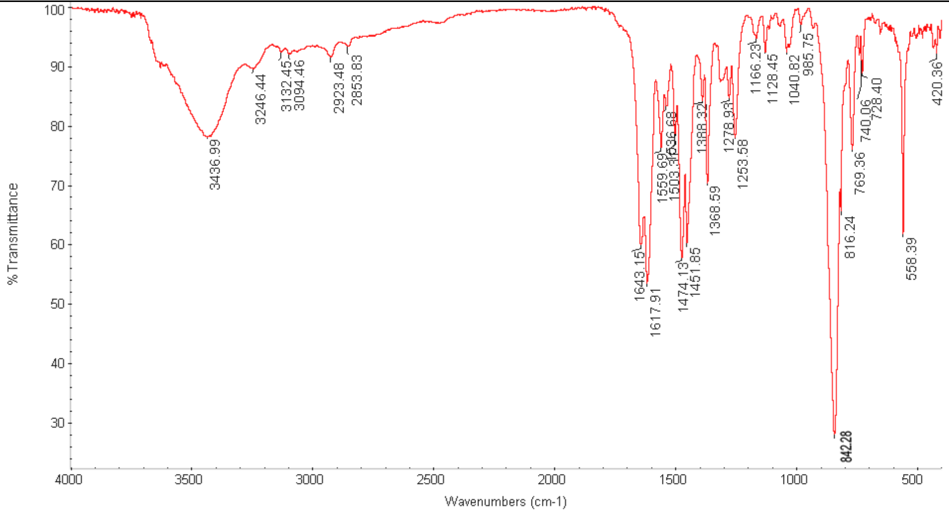

Complex **5**

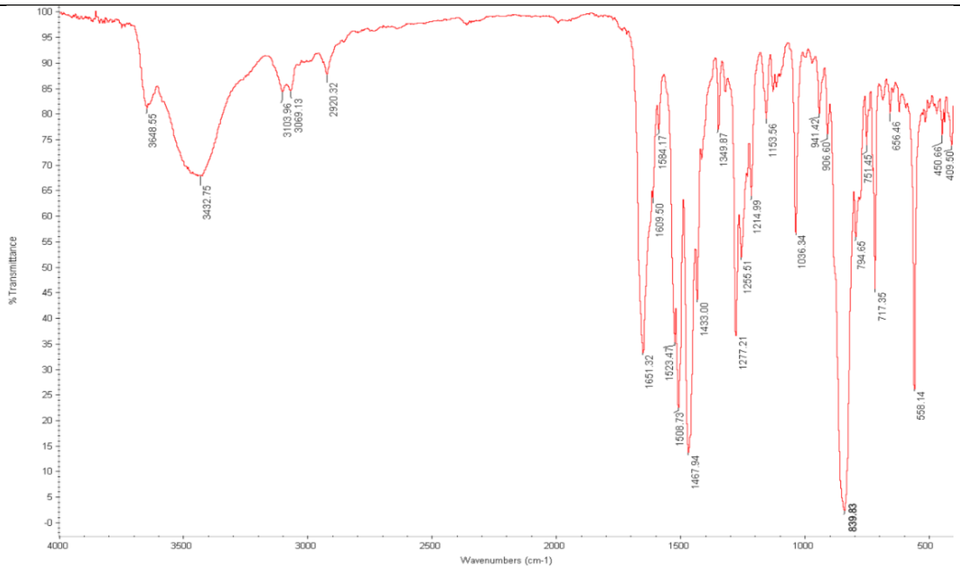

Complex **6**

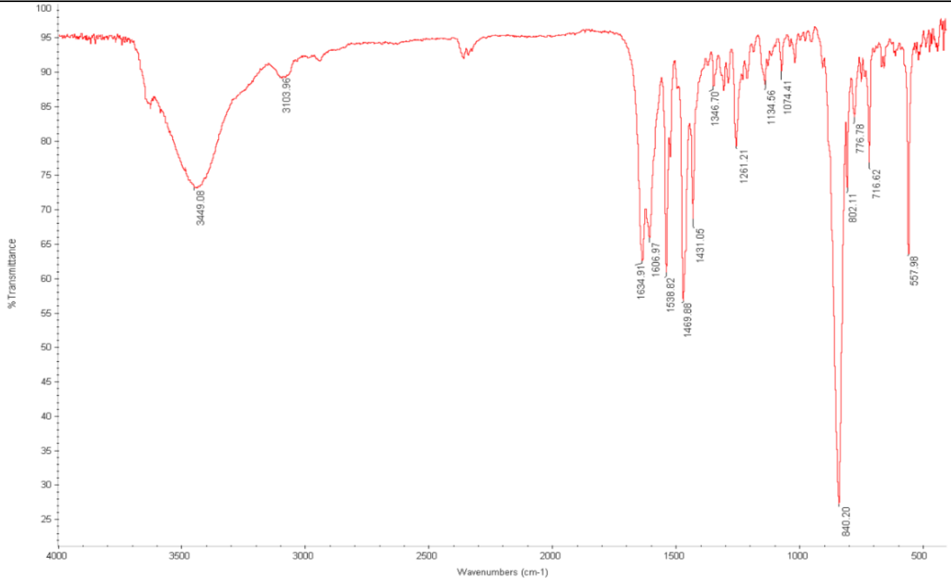
% Transmittance
Wavenumbers (cm-1)
3449.08
3103.96
1634.91
1606.97
1538.82
1469.88
1431.05
1346.70
1261.21
1134.56
1074.41
840.20
802.11
776.78
716.62
557.98

Figure S2. ^{1}H NMR spectra of the complexes in a 95:5 mixture of D_2O : DMSO-d_6.

Complex **1**

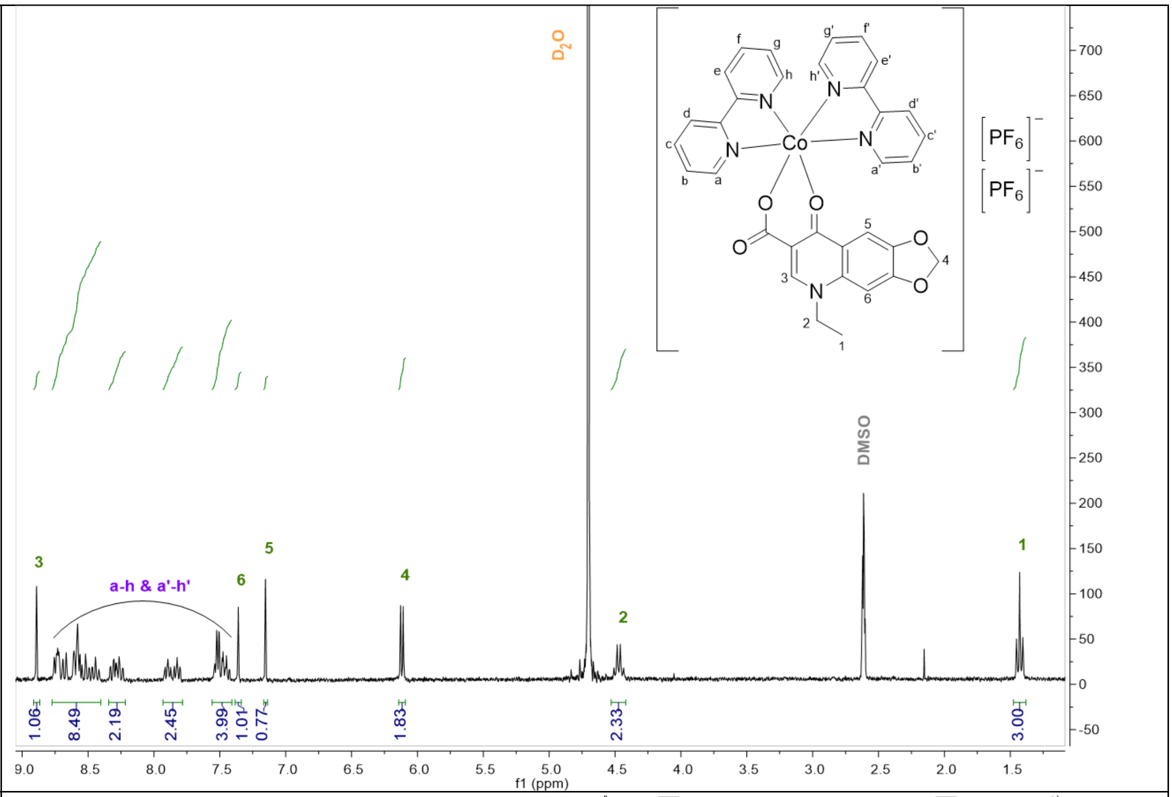

Complex **2**

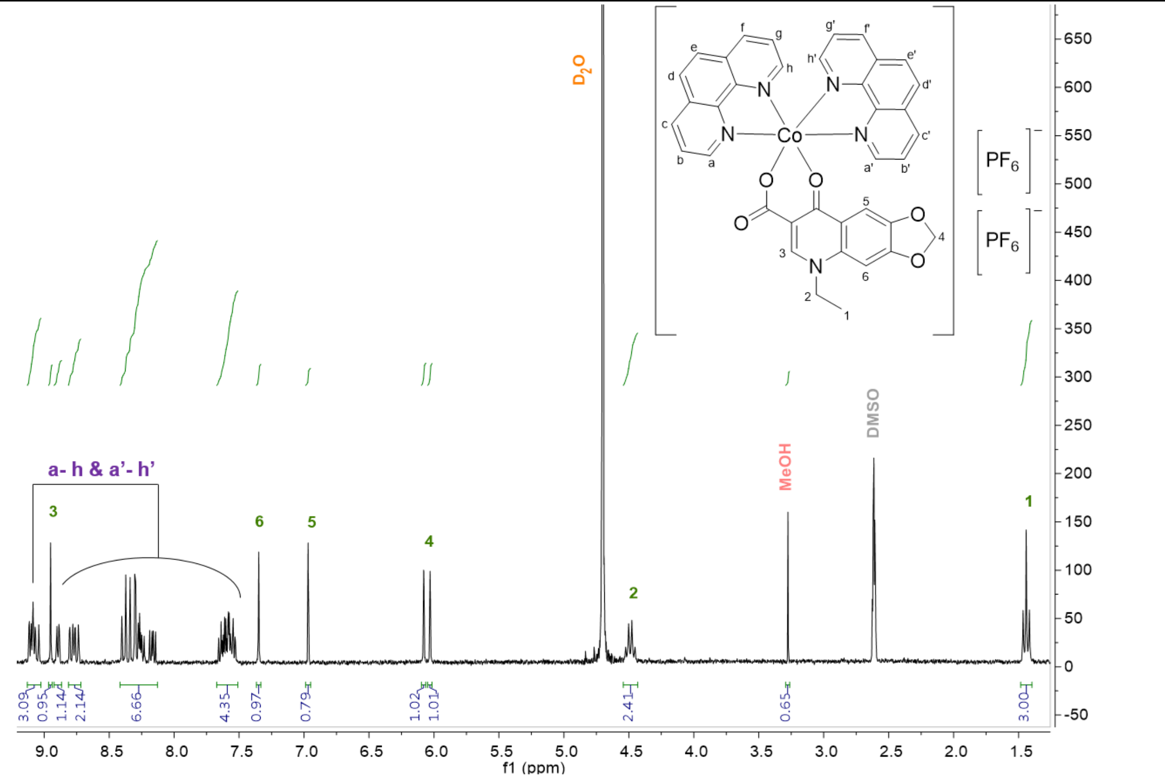

Complex **3**

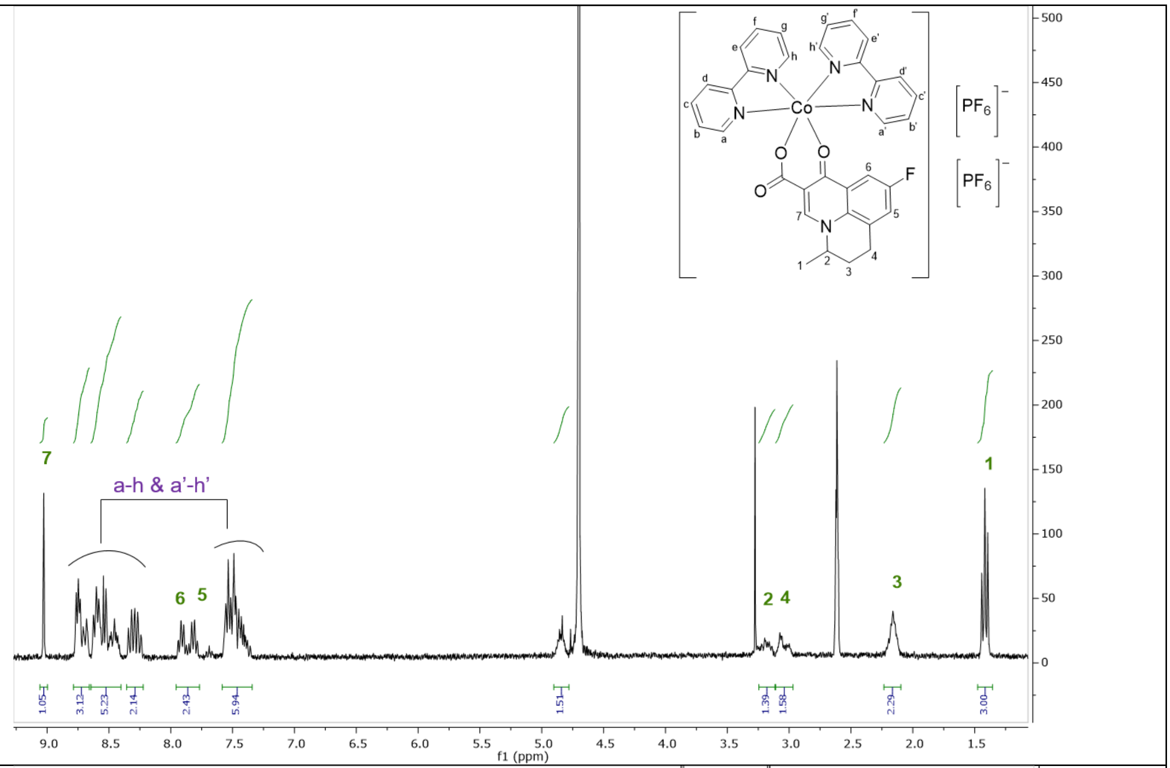

Complex **4**

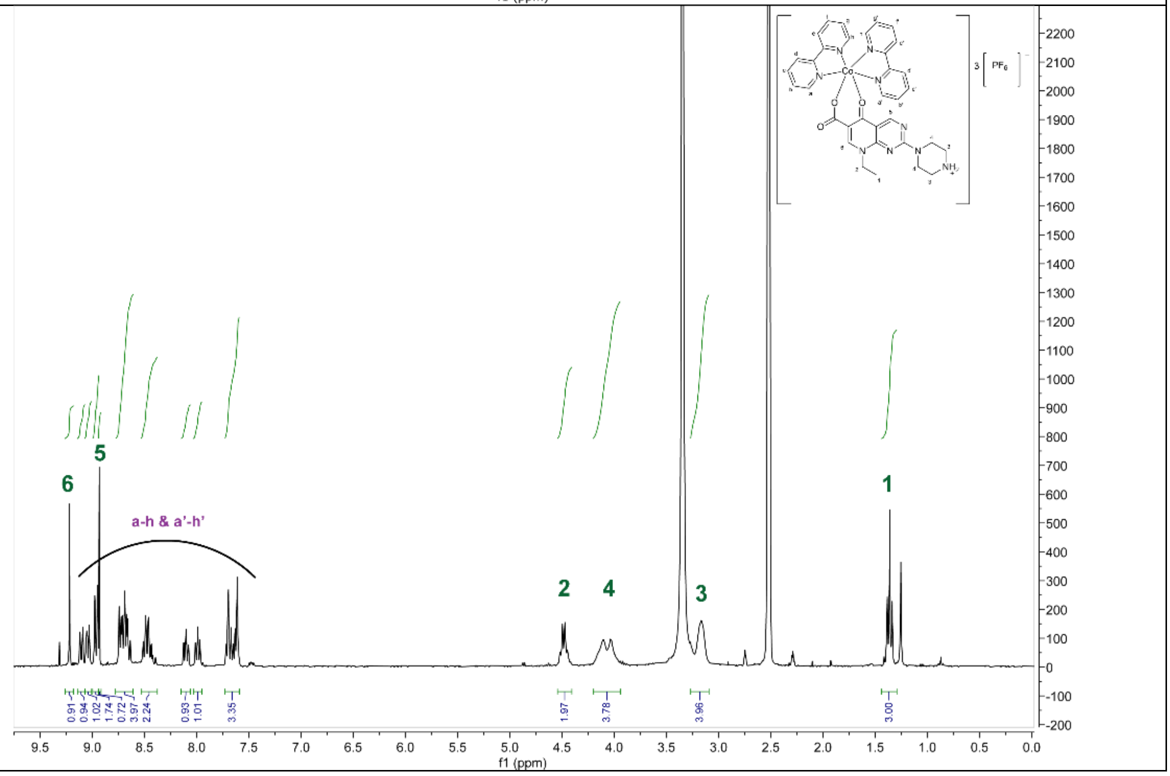

Complex **5**

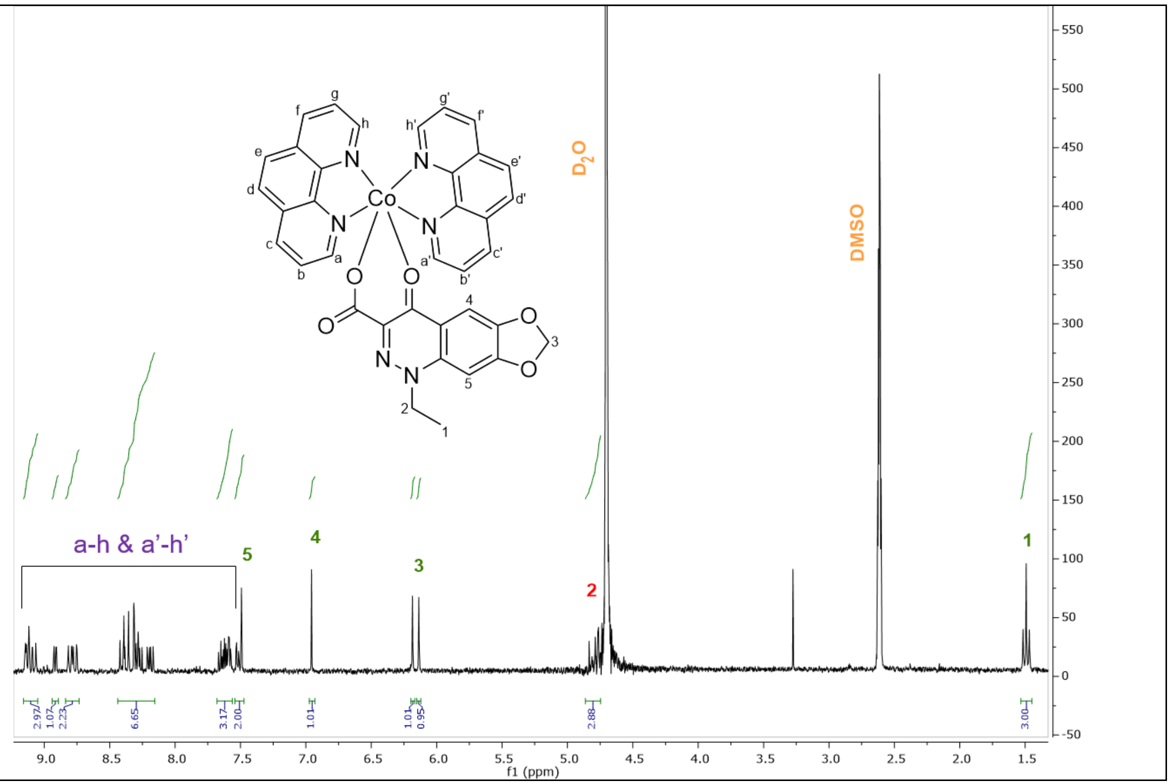

a-h & a'-h'
5
4
3
2
1
D2O
DMSO
Co
2.97
1.07
2.23
6.65
3.17
2.00
1.01
1.01
0.95
2.88
3.00
f1 (ppm)

Figure S3. ^{1}H NMR spectra of complex **1** in a 95:5 mixture of D_2O : DMSO-d_6 for two different time intervals (0 h and 24 h).

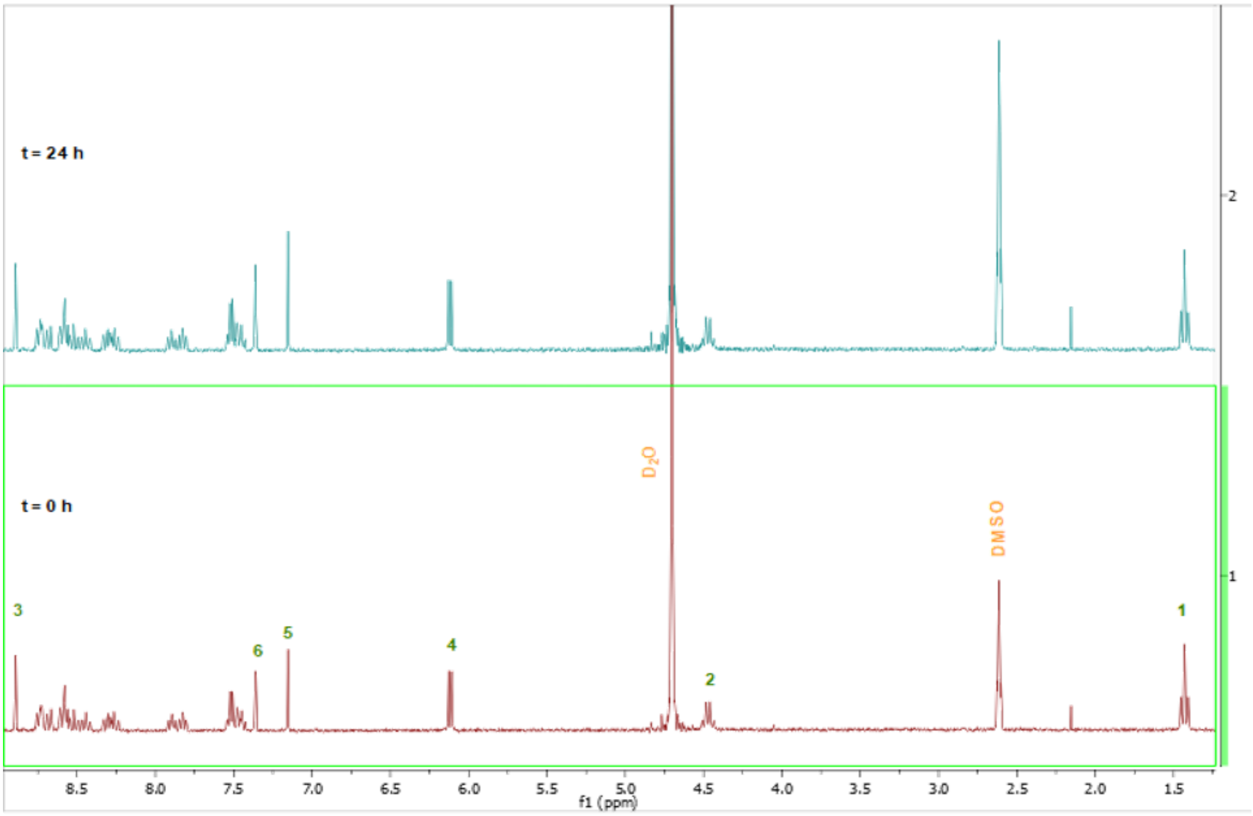

Figure S4. UV–vis spectra of a CT DNA buffer solution, in the presence of increasing amounts of the complexes.
The arrows show the changes upon increasing amounts of the compounds.

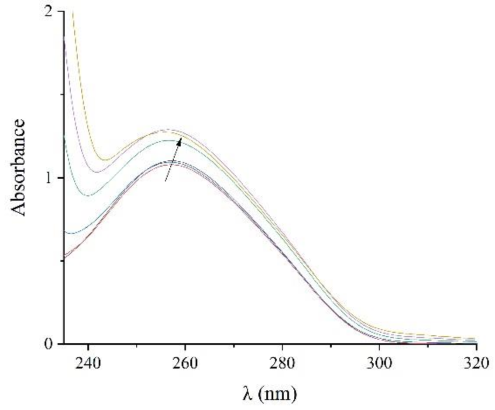	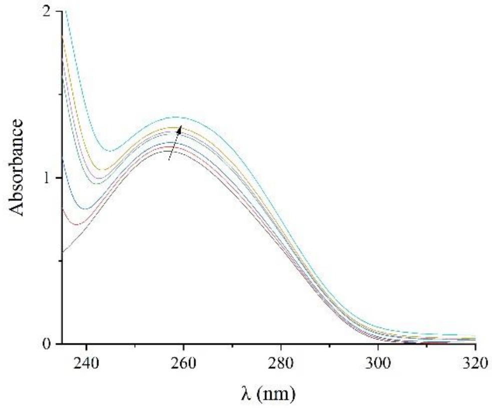
Complex **1**	Complex **2**
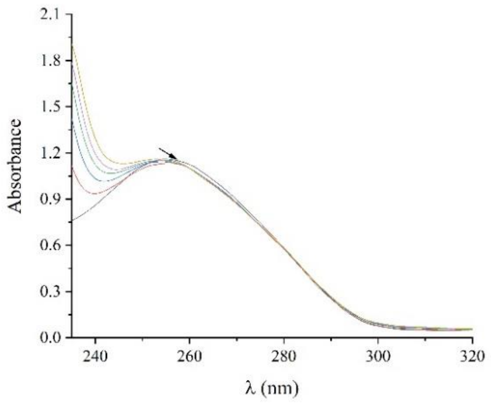	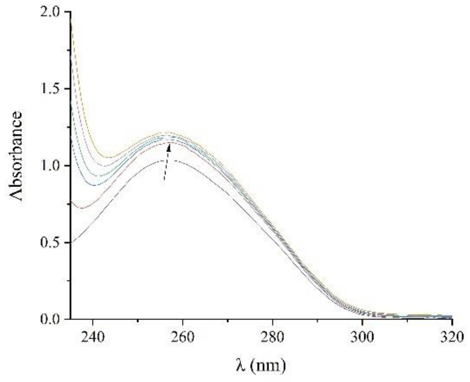
Complex **3**	Complex **6**
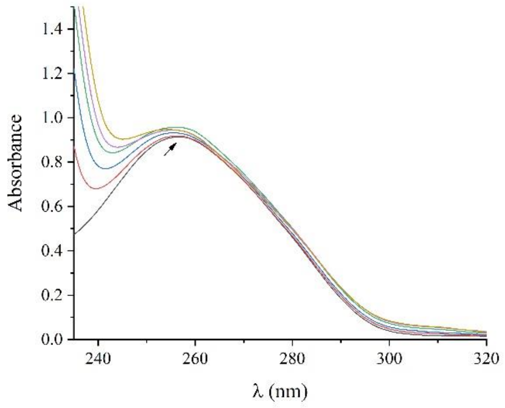	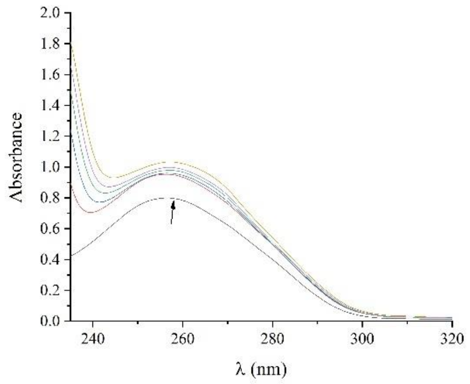
Complex **a**	Complex **b**

Figure S5. UV−vis spectra of a DMSO solution of the complexes, in the presence of increasing amounts of CT DNA.

The concentrations of the solution of the compounds are given in parentheses. The arrows show the changes upon increasing amounts of CT DNA.

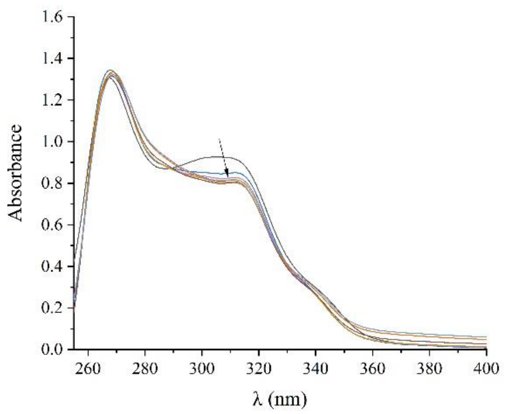

Complex **1** (30 μM)

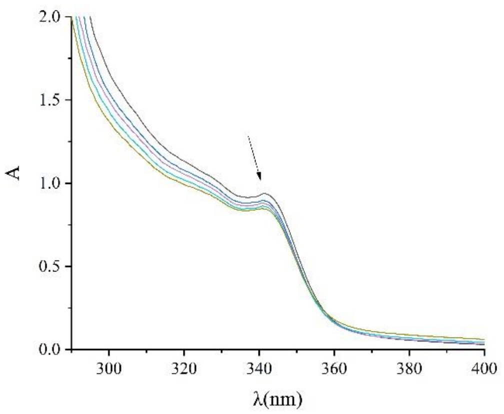

Complex **2** (50 μM)

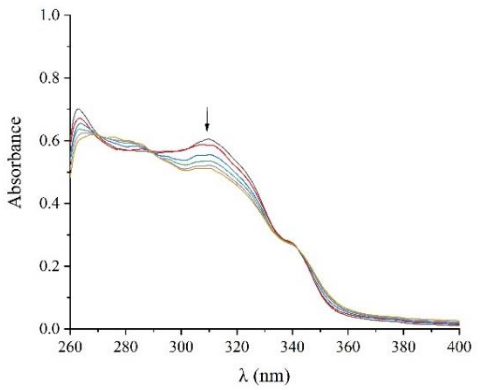

Complex **3** (30 μM)

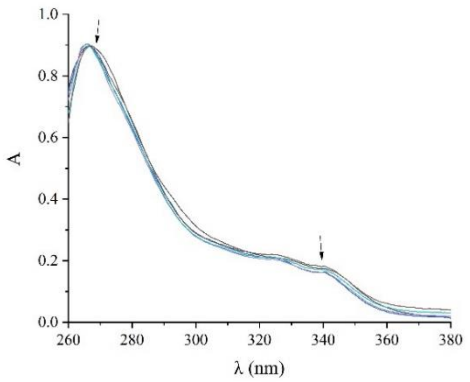

Complex **6** (40 μM)

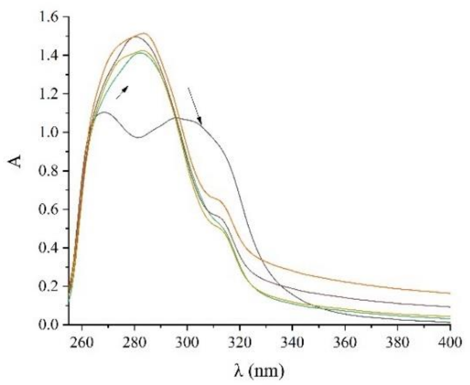

Complex **a** (50 μM)

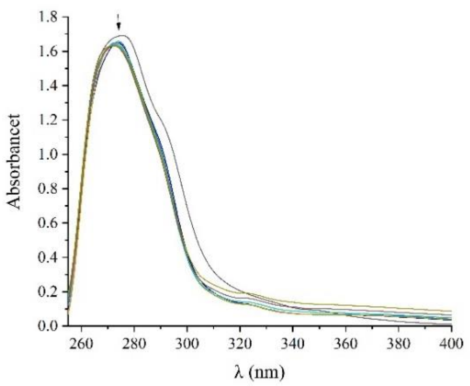

Complex **b** (50 μM)

Figure S6. Plots of $\frac{[\mathrm{DNA}]}{(\varepsilon_A-\varepsilon_f)}$ *versus* [DNA] for the complexes.

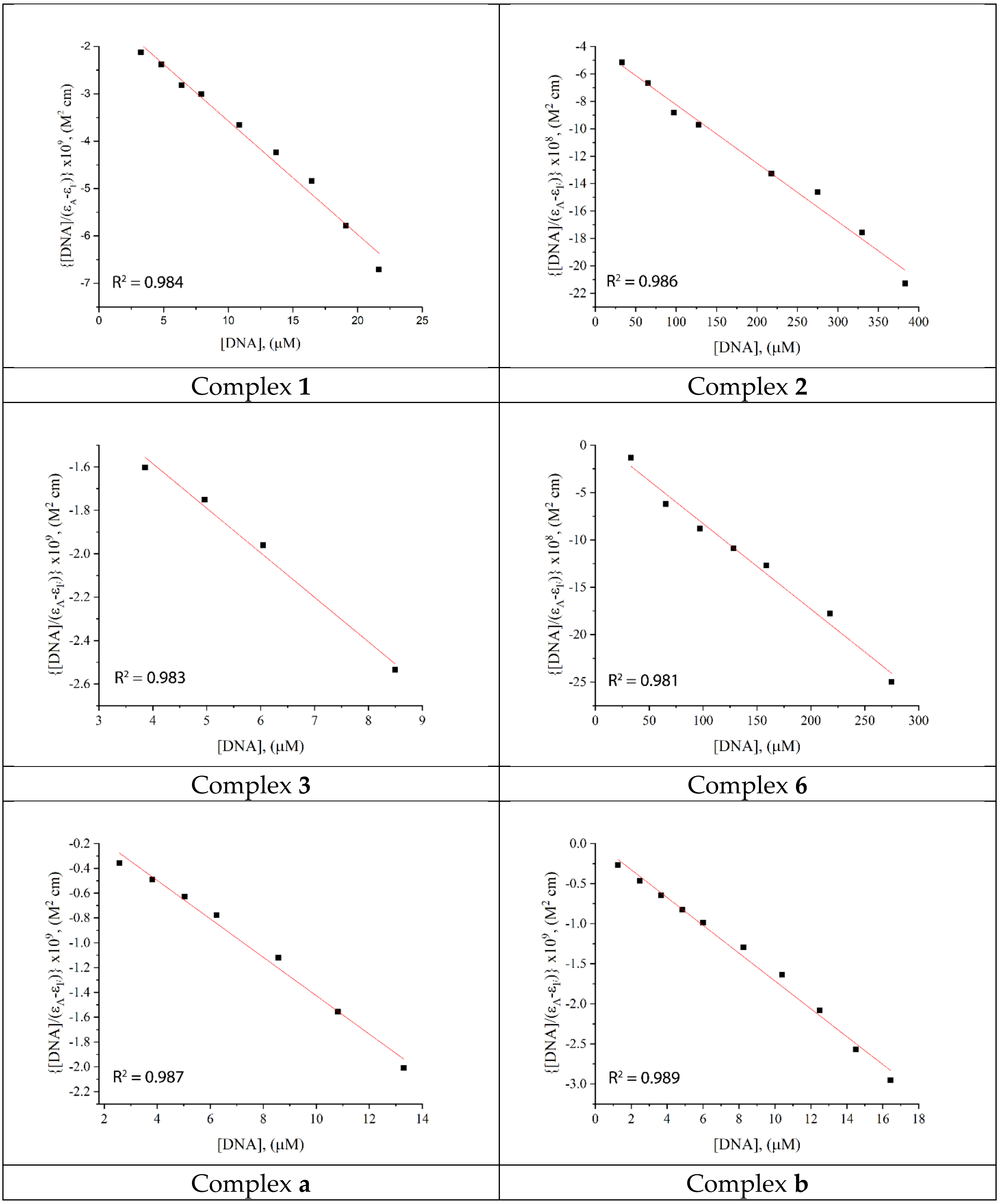

Figure S7. Cyclic voltammograms of the complexes in the absence (black line) or presence (green line) of CT DNA.

12 mL solution of the complexes (0.33 mM, 1/2 DMSO/buffer) were used. Scan rate = 100 mV s^{-1}. Supporting electrolyte = buffer solution. The arrows show the changes upon addition of CT DNA (0.5 mL CT DNA 3 mM).

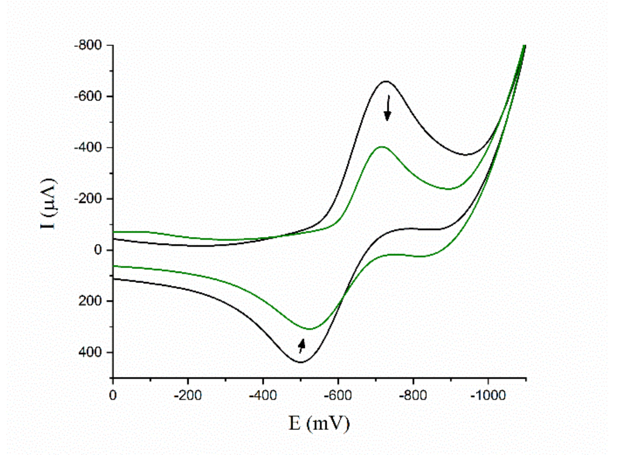

Complex **1**

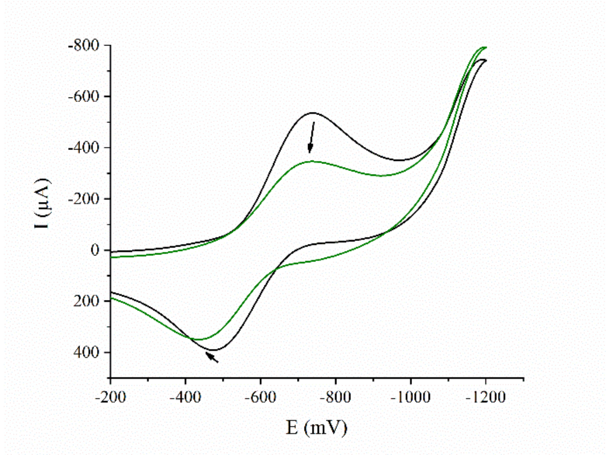

Complex **2**

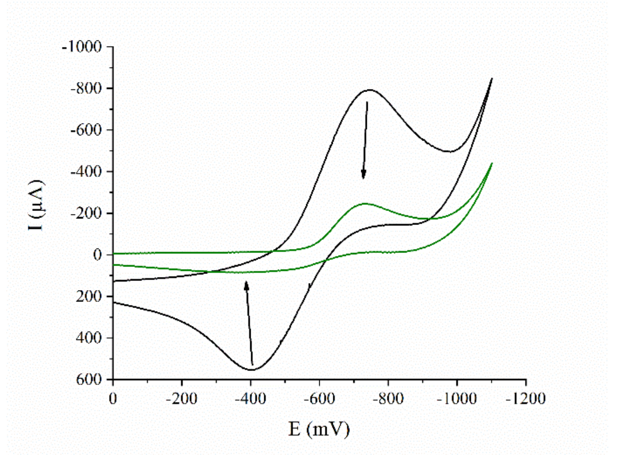

Complex **3**

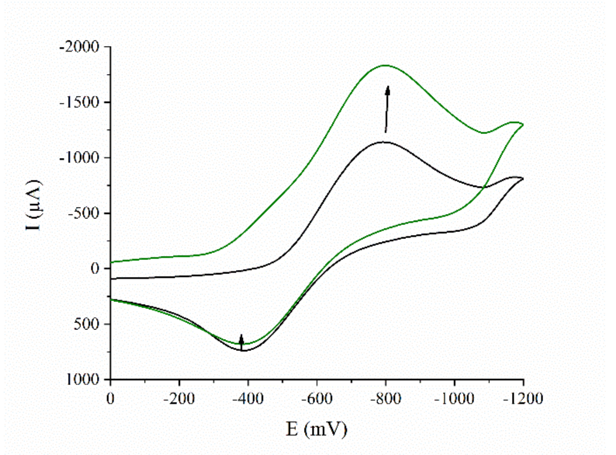

Complex **6**

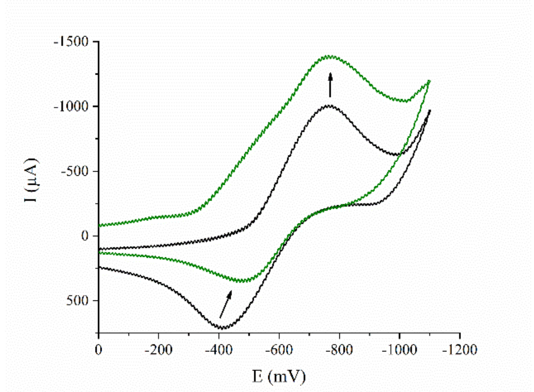

Complex **a**

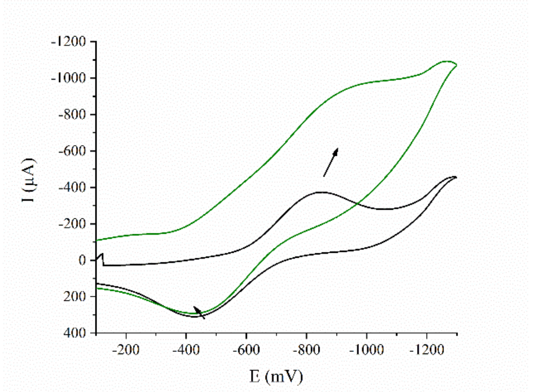

Complex **b**

Figure S8. Fluorescence emission spectra for EB-DNA in buffer solution in the absence and presence of increasing amounts of the compounds.
Conditions: $\lambda_{excitation}$ = 540 nm. [EB] = 40 μM. [DNA] = 45 μM. Buffer solution: 150 mM NaCl and 15 mM trisodium citrate at pH 7.0. The arrow shows the changes of intensity upon increasing amounts of the compound.

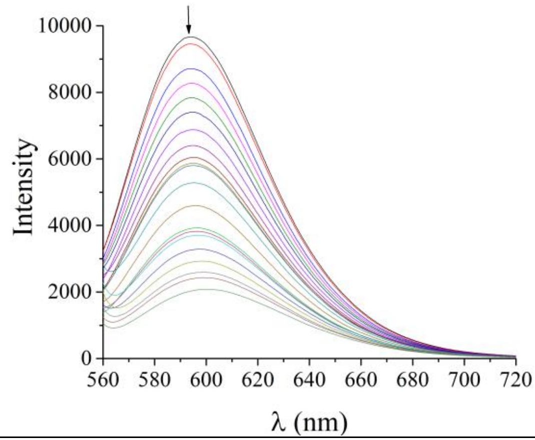

Complex **1**

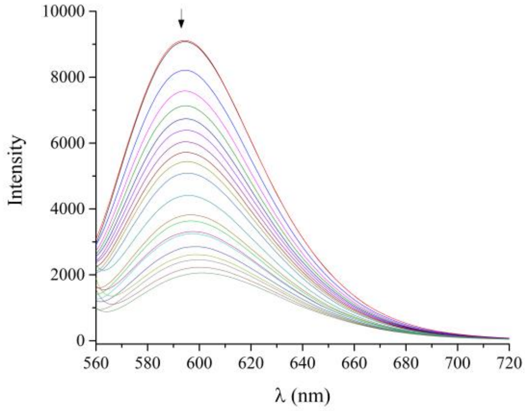

Complex **2**

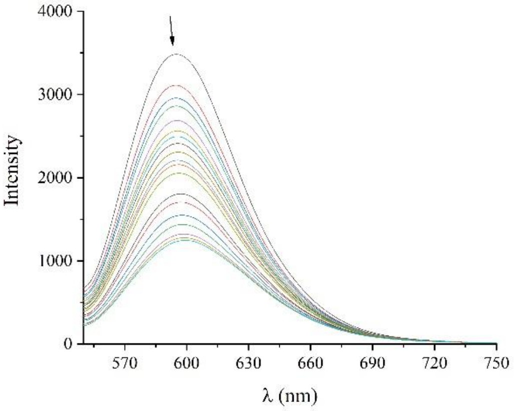

Complex **3**

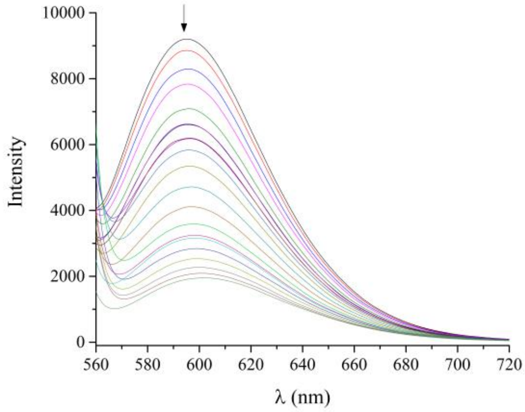

Complex **6**

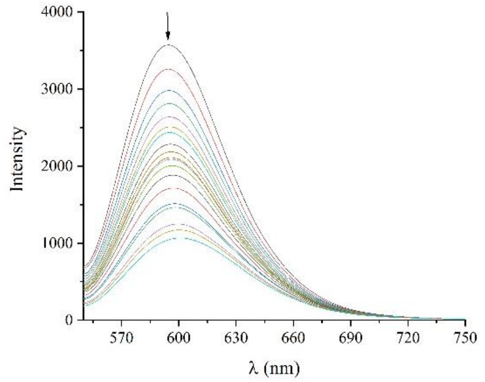

Complex **a**

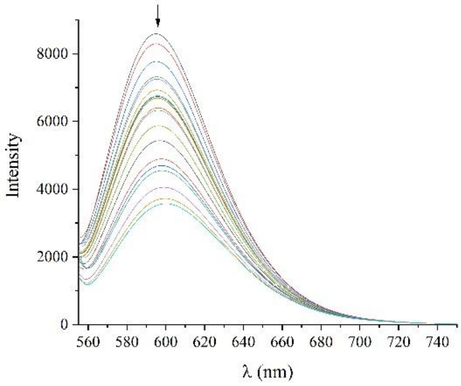

Complex **b**

Figure S9. Stern–Volmer plots of the EB–DNA quenching experiments upon addition of the compounds.

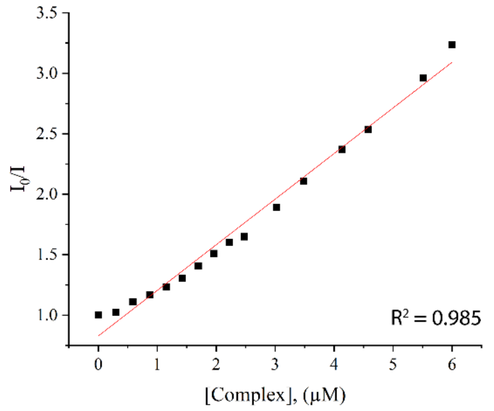

Complex **1**

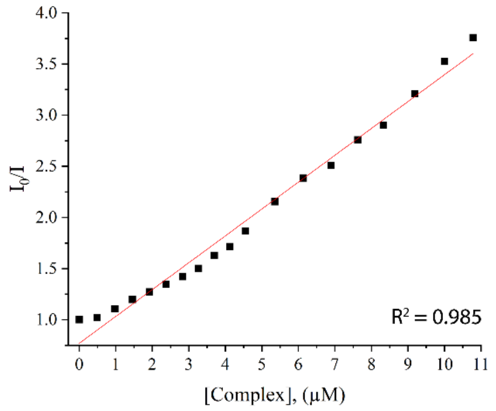

Complex **2**

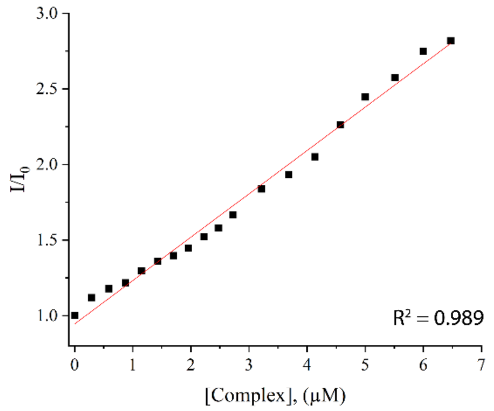

Complex **3**

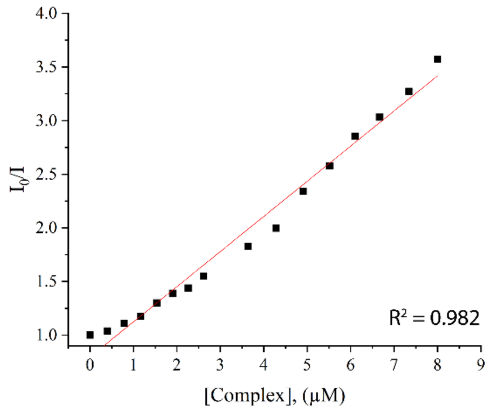

Complex **6**

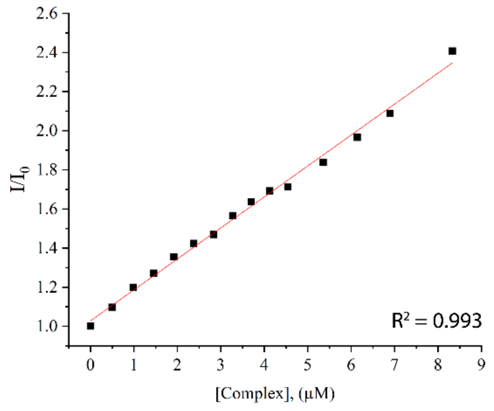

Complex **a**

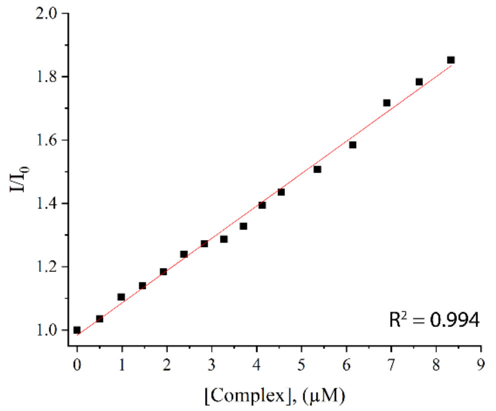

Complex **b**

Figure S10. Fluorescence emission spectra of BSA in buffer solution in the presence of increasing amounts of the compounds.
Conditions: $\lambda_{excitation}$ = 295 nm. [BSA] = 3 μM. Buffer solution: 150 mM NaCl and 15 mM trisodium citrate at pH 7.0. The arrow shows the changes of intensity upon increasing amounts of the compound.

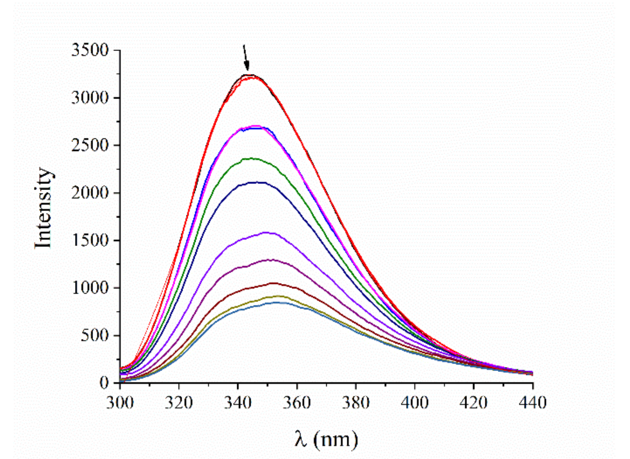

Complex **1**

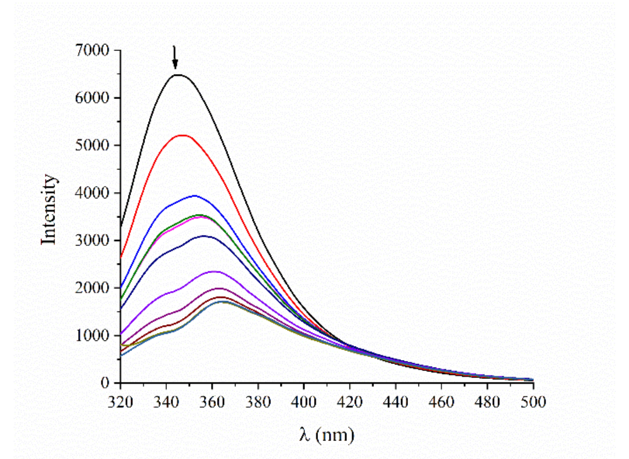

Complex **2**

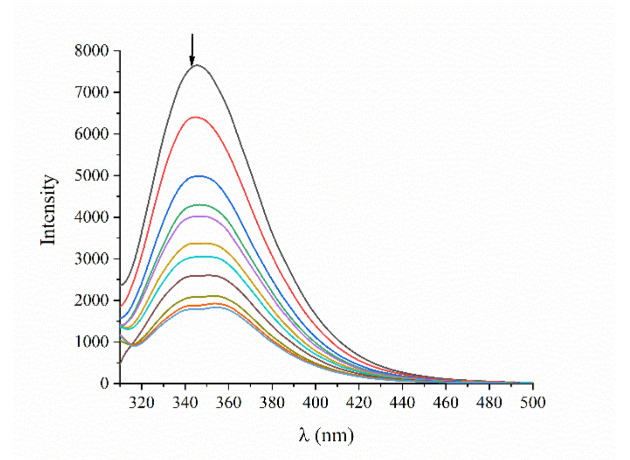

Complex **3**

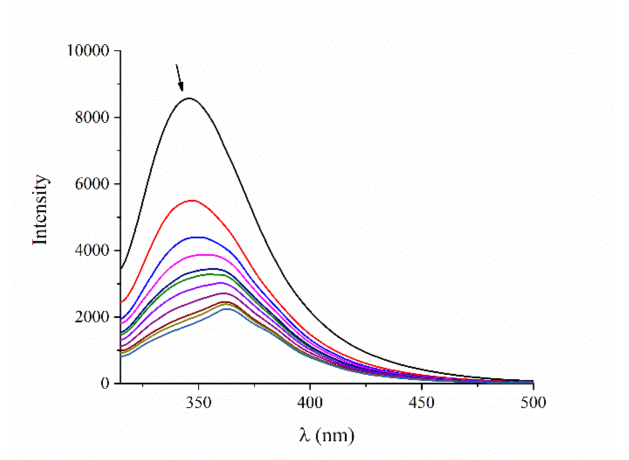

Complex **6**

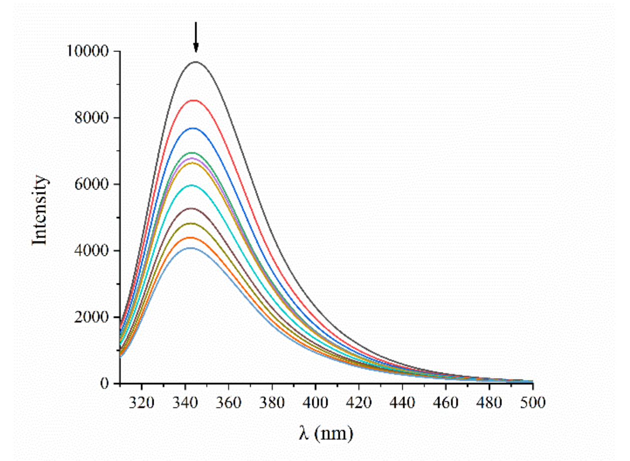

Complex **a**

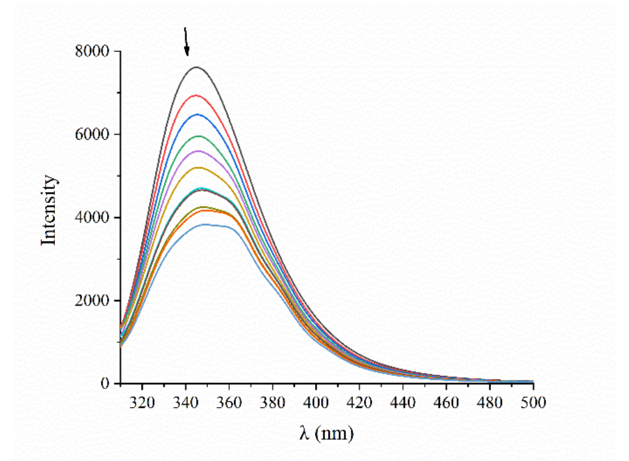

Complex **b**

Figure S11. Fluorescence emission spectra of HSA in buffer solution in the presence of increasing amounts of the compounds.
Conditions: $\lambda_{excitation}$= 295 nm. [HSA] = 3 µM. Buffer solution: 150 mM NaCl and 15 mM trisodium citrate at pH 7.0. The arrow shows the changes of intensity upon increasing amounts of the compound.

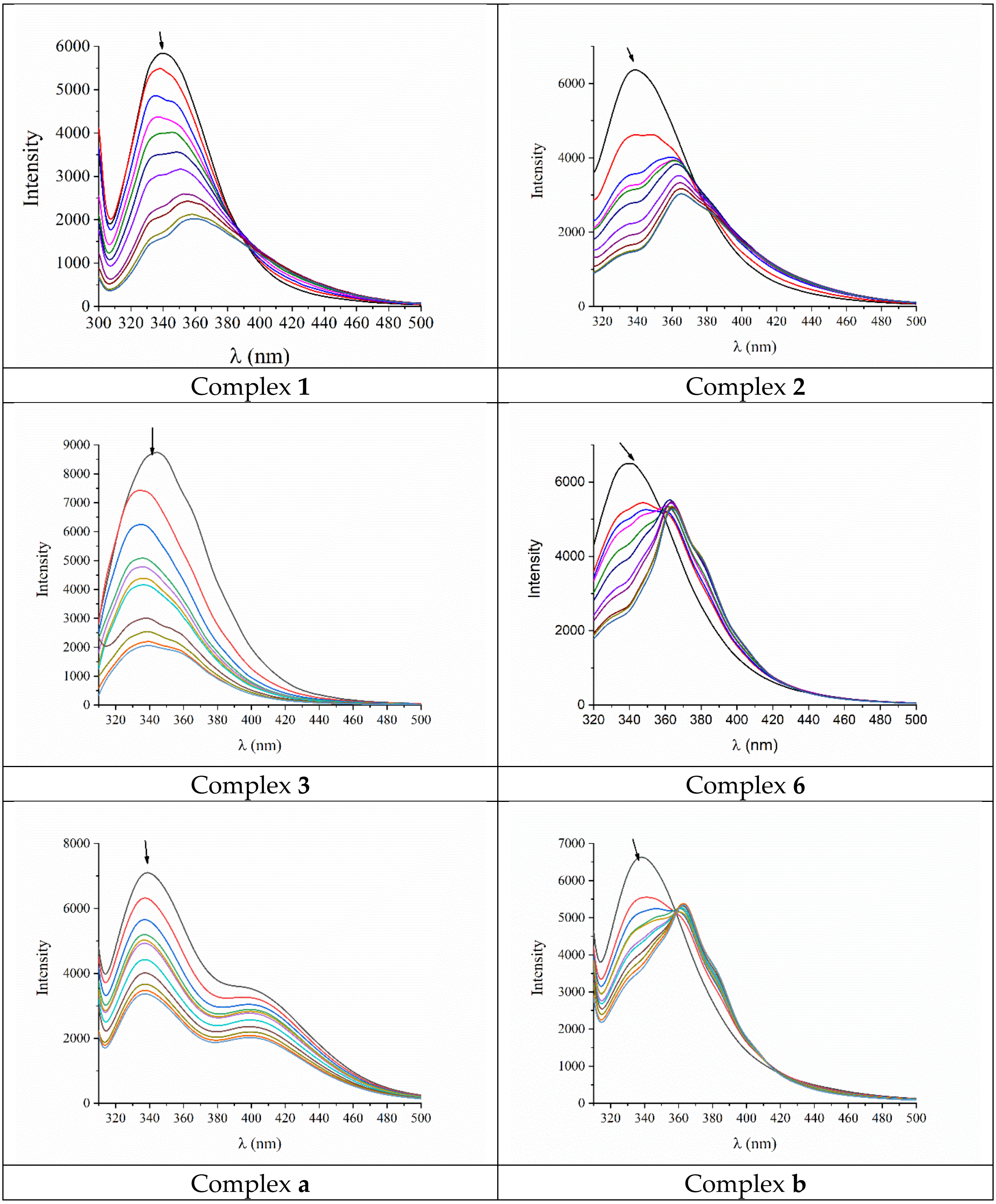

Figure S12. Stern–Volmer plots of the BSA-quenching experiments upon addition of the compounds.

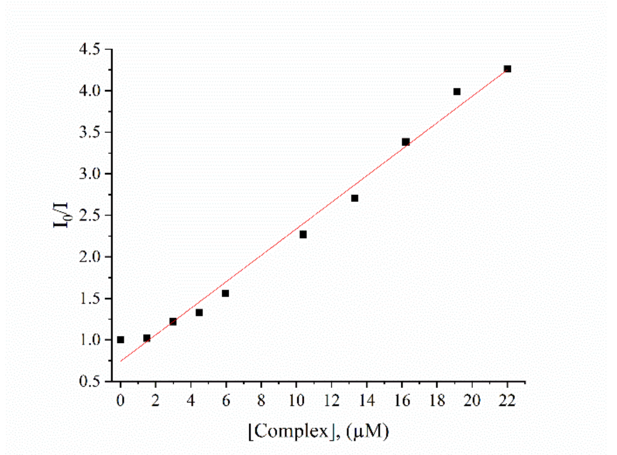

Complex **1**

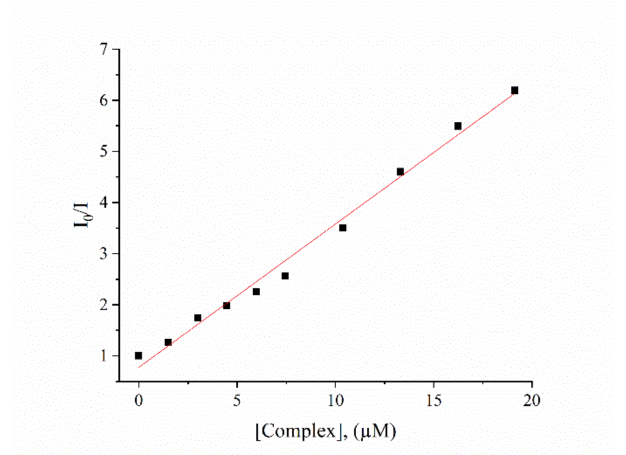

Complex **2**

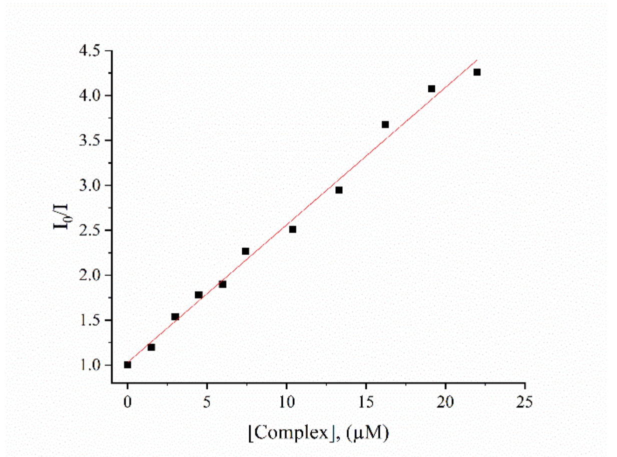

Complex **3**

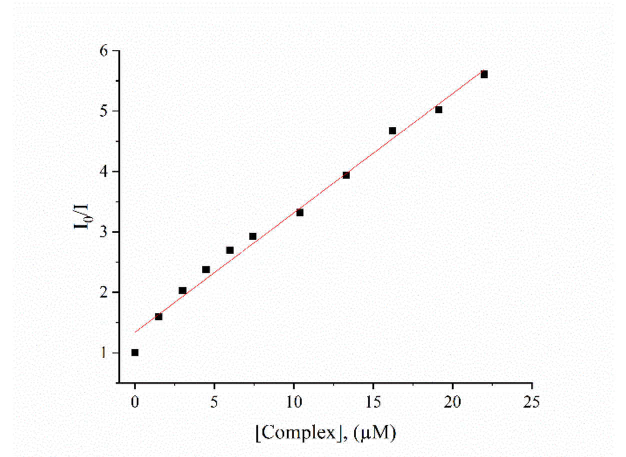

Complex **6**

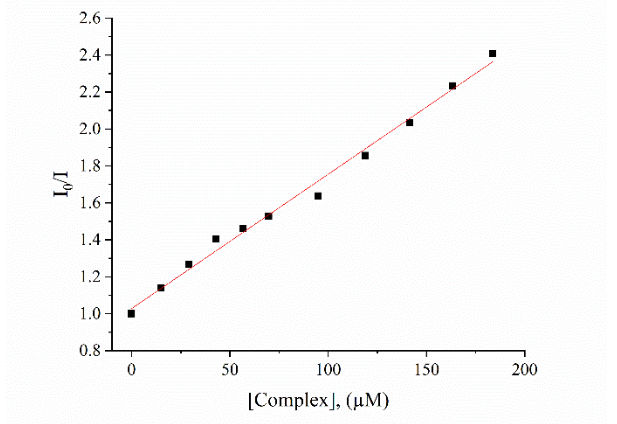

Complex **a**

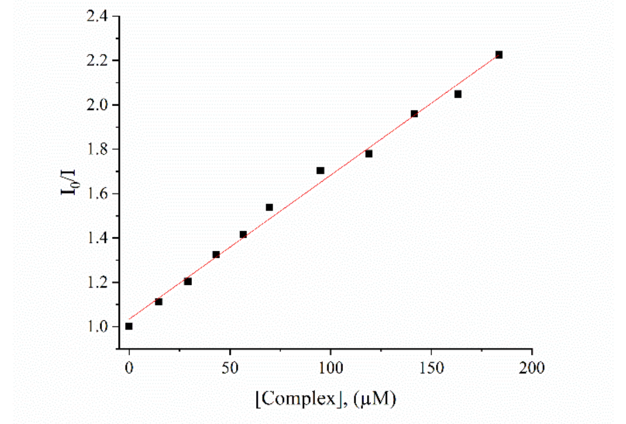

Complex **b**

Figure S13. Stern–Volmer plots of the HSA-quenching experiments upon addition of the compounds.

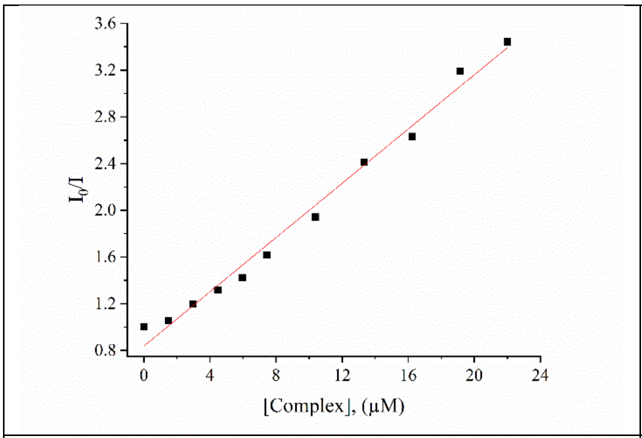

Complex **1**

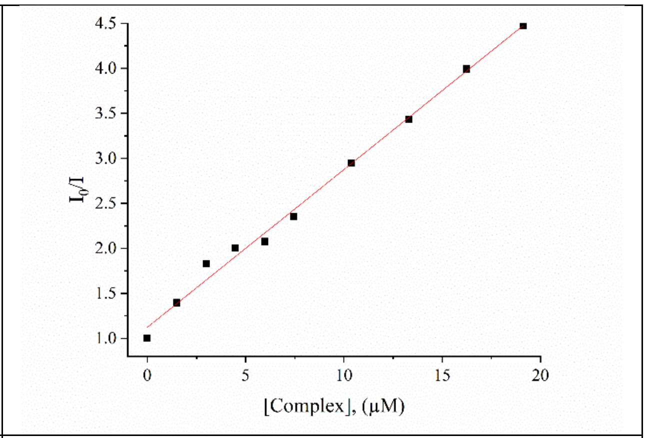

Complex **2**

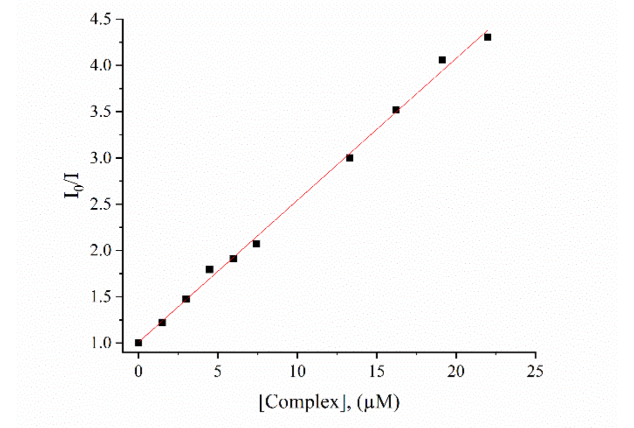

Complex **3**

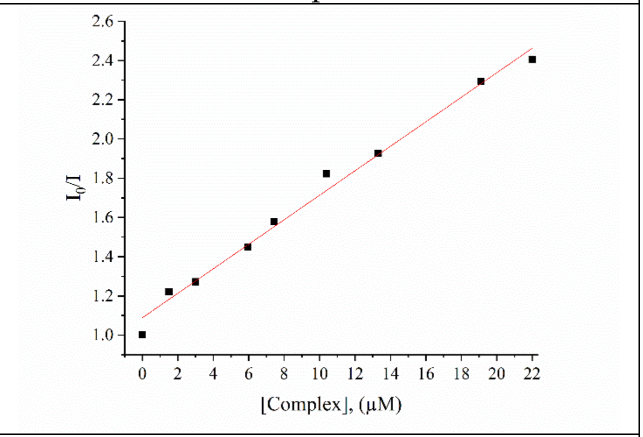

Complex **6**

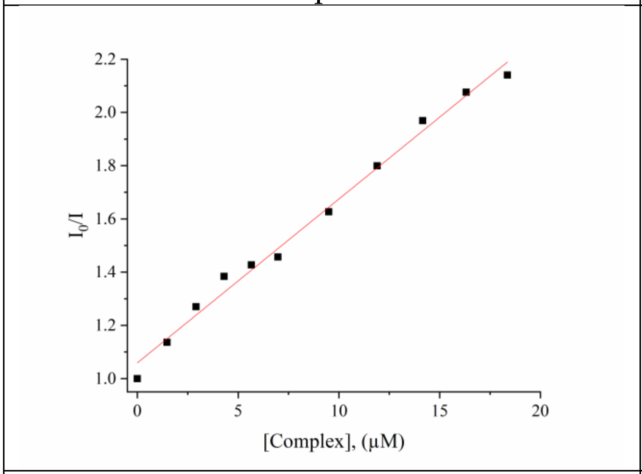

Complex **a**

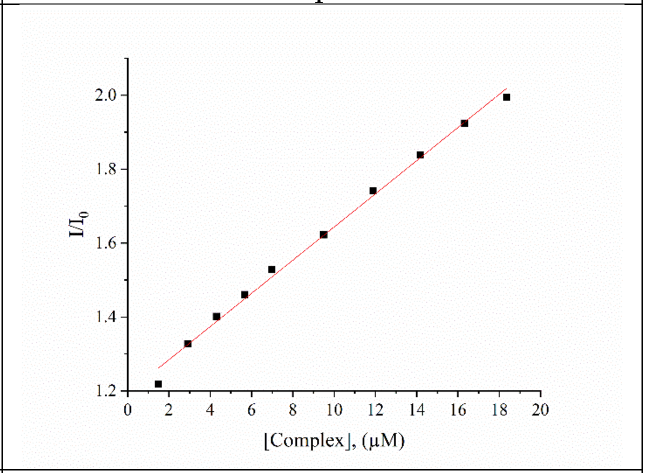

Complex **b**

Figure S14. Scatchard plots of the BSA-quenching experiments upon addition of the compounds.

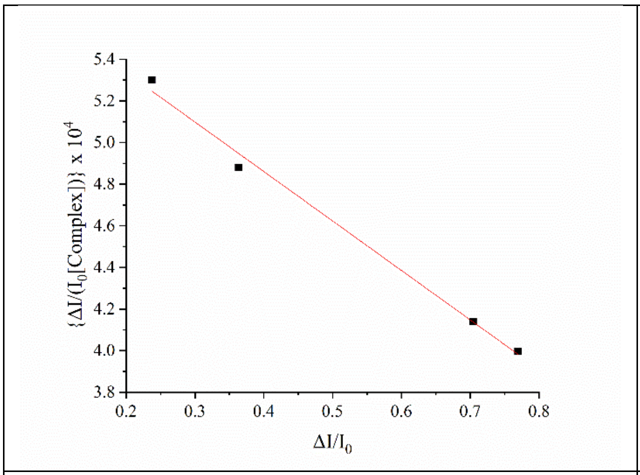

Complex **1**

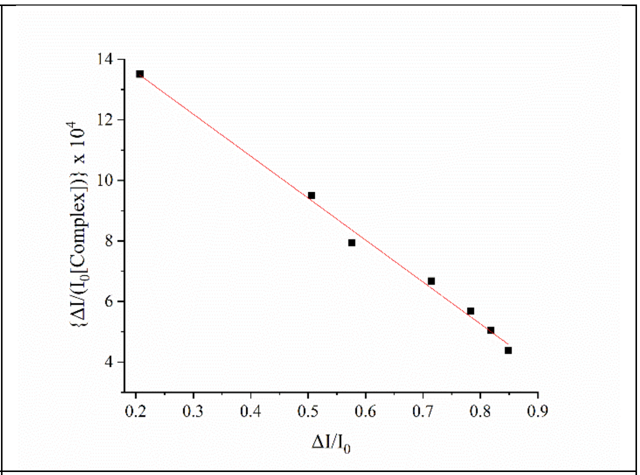

Complex **2**

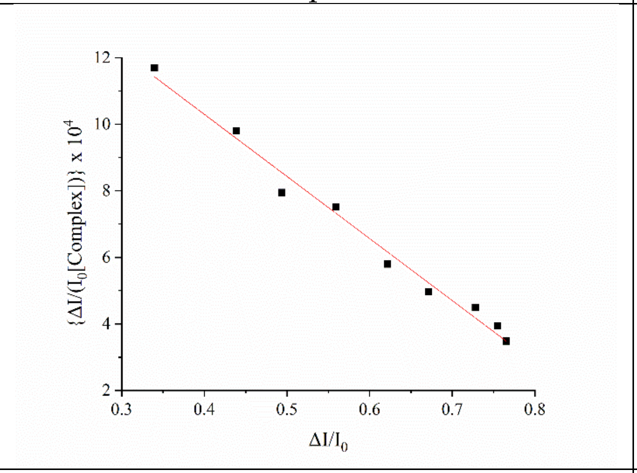

Complex **3**

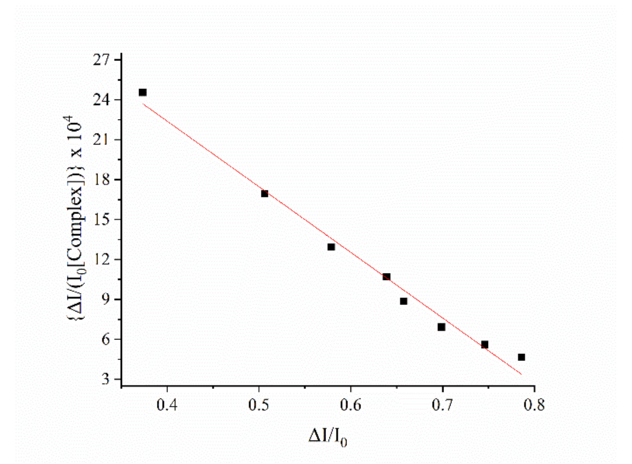

Complex **4**

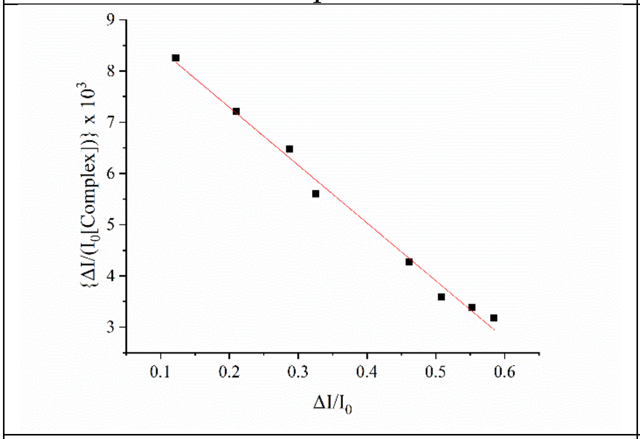

Complex **a**

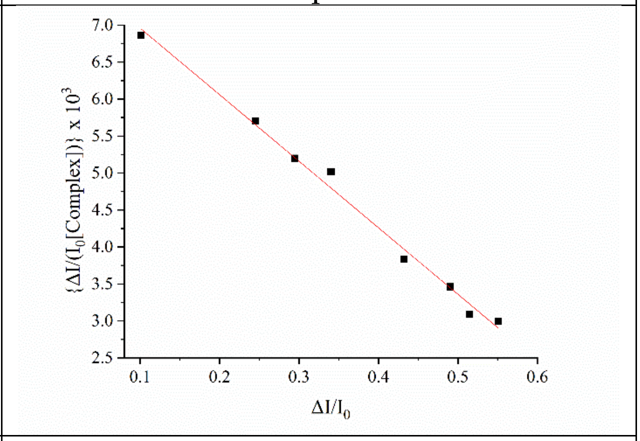

Complex **b**

Figure S15. Scatchard plots of the HSA-quenching experiments upon addition of the compounds.

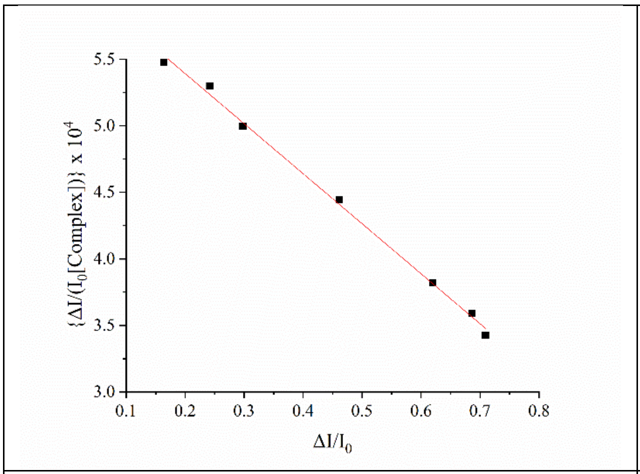

Complex **1**

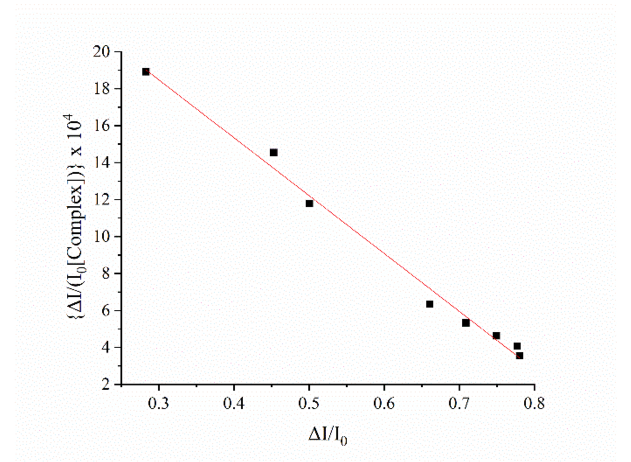

Complex **2**

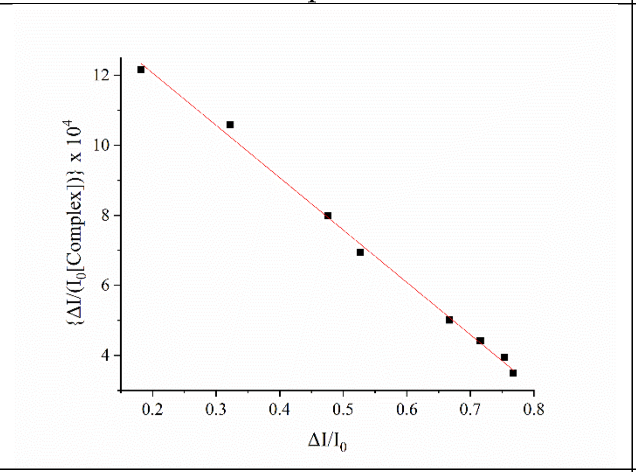

Complex **3**

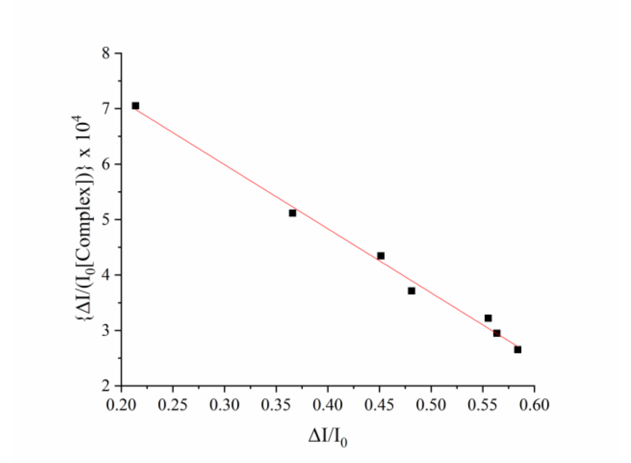

Complex **6**

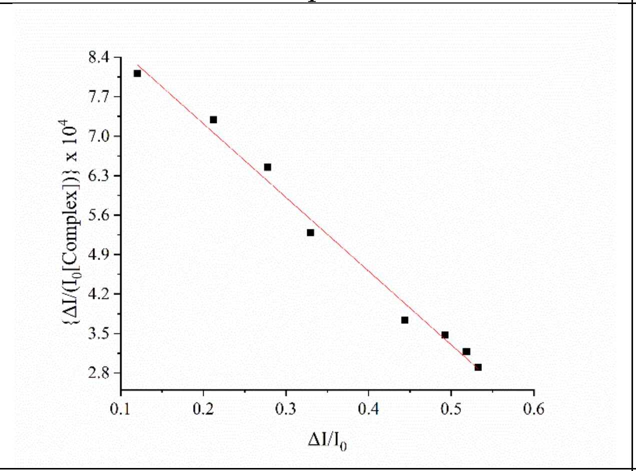

Complex **a**

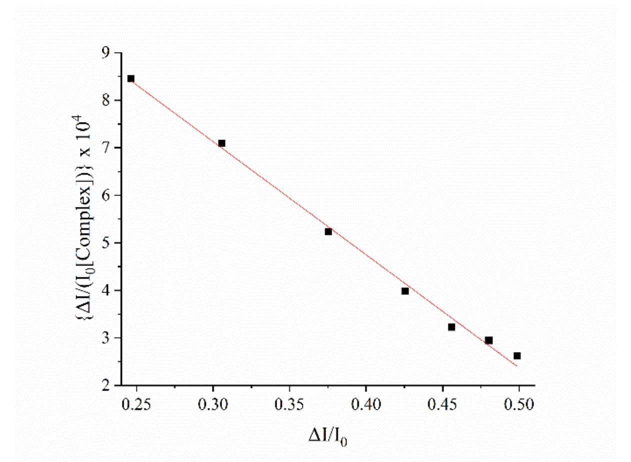

Complex **b**
