# Supplementary material for: Synthesis, Characterization and Biological Profile of Cationic Cobalt Complexes with First-Generation Quinolones
Source: Molecules. 2025 Jun 19;30(12):2646. doi: 10.3390/molecules30122646 (PMC12196173; doi:10.3390/molecules30122646)

checkCIF/PLATON report

Structure factors have been supplied for datablock(s) I

THIS REPORT IS FOR GUIDANCE ONLY. IF USED AS PART OF A REVIEW PROCEDURE FOR PUBLICATION, IT SHOULD NOT REPLACE THE EXPERTISE OF AN EXPERIENCED CRYSTALLOGRAPHIC REFEREE.

No syntax errors found.   CIF dictionary   Interpreting this report

Datablock: I

Bond precision:	C-C = 0.0057 A		Wavelength=0.71073
Cell:	a=16.218(2)	b=14.0178(19)	c=17.274(2)
	alpha=90	beta=95.715(4)	gamma=90
Temperature:	295 K		

	Calculated	Reported
Volume	3907.6(8)	3907.5(9)
Space group	P 21/c	P 21/c
Hall group	-P 2ybc	?
Moiety formula	2(C34 H27 Co F N5 O3), 4(F6 P), C H4 O, H2 O	C34 H27 Co F N5 O3, 2(F6P), 0.5(C H4 O), 0.5(H2 O)
Sum formula	C69 H60 Co2 F26 N10 O8 P4	C34.50 H30 Co1 F13 N5 O4 P2
Mr	1893.01	946.50
Dx,g cm-3	1.609	1.609
Z	2	4
Mu (mm-1)	0.629	0.629
F000	1912.0	1912.0
F000'	1915.54	
h,k,lmax	19,17,21	19,17,21
Nref	7470	7446
Tmin,Tmax	0.866,0.910	0.890,0.910
Tmin'	0.860	

Correction method= # Reported T Limits: Tmin=0.890 Tmax=0.910
AbsCorr = NUMERICAL

Data completeness= 0.997   Theta(max)= 25.745

R(reflections)= 0.0521(5688)   wR2(reflections)= 0.0994(5688)

S = 1.000   Npar= 525

The following ALERTS were generated. Each ALERT has the format
test-name_ALERT_alert-type_alert-level.
Click on the hyperlinks for more details of the test.

Alert level C

```
PLAT041_ALERT_1_C Calc. and Reported SumFormula    Strings  Differ     Please Check
PLAT042_ALERT_1_C Calc. and Reported MoietyFormula Strings  Differ     Please Check
PLAT220_ALERT_2_C NonSolvent   Resd 1  C   Ueq(max)/Ueq(min) Range        4.8 Ratio
PLAT241_ALERT_2_C High   'MainMol' Ueq as Compared to Neighbors of        C21 Check
PLAT260_ALERT_2_C Large Average Ueq of Residue Including        P1      0.132 Check
PLAT911_ALERT_3_C Missing FCF Refl Between Thmin & STh/L=    0.600         11 Report
PLAT992_ALERT_5_C Repd & Actual _reflns_number_gt Values Differ by         24 Check
```

Alert level G

```
PLAT002_ALERT_2_G Number of Distance or Angle Restraints on AtSite         15 Note
PLAT007_ALERT_5_G Number of Unrefined Donor-H Atoms .............           3 Report
PLAT045_ALERT_1_G Calculated and Reported Z Differ by a Factor ...      0.500 Check
PLAT244_ALERT_4_G Low    'Solvent' Ueq as Compared to Neighbors of         P2 Check
PLAT244_ALERT_4_G Low    'Solvent' Ueq as Compared to Neighbors of         P1 Check
PLAT300_ALERT_4_G Atom Site Occupancy of F12        Constrained at        0.5 Check
PLAT300_ALERT_4_G Atom Site Occupancy of F13        Constrained at        0.5 Check
PLAT300_ALERT_4_G Atom Site Occupancy of F14        Constrained at        0.5 Check
PLAT300_ALERT_4_G Atom Site Occupancy of F15        Constrained at        0.5 Check
PLAT300_ALERT_4_G Atom Site Occupancy of F16        Constrained at        0.5 Check
PLAT300_ALERT_4_G Atom Site Occupancy of F17        Constrained at        0.5 Check
PLAT300_ALERT_4_G Atom Site Occupancy of F18        Constrained at        0.5 Check
PLAT300_ALERT_4_G Atom Site Occupancy of F19        Constrained at        0.5 Check
PLAT300_ALERT_4_G Atom Site Occupancy of F20        Constrained at        0.5 Check
PLAT300_ALERT_4_G Atom Site Occupancy of F21        Constrained at        0.5 Check
PLAT300_ALERT_4_G Atom Site Occupancy of F22        Constrained at        0.5 Check
PLAT300_ALERT_4_G Atom Site Occupancy of F23        Constrained at        0.5 Check
PLAT300_ALERT_4_G Atom Site Occupancy of F4         Constrained at        0.5 Check
PLAT300_ALERT_4_G Atom Site Occupancy of F5         Constrained at        0.5 Check
PLAT300_ALERT_4_G Atom Site Occupancy of F6         Constrained at        0.5 Check
PLAT300_ALERT_4_G Atom Site Occupancy of F7         Constrained at        0.5 Check
PLAT300_ALERT_4_G Atom Site Occupancy of F8         Constrained at        0.5 Check
PLAT300_ALERT_4_G Atom Site Occupancy of F9         Constrained at        0.5 Check
PLAT300_ALERT_4_G Atom Site Occupancy of F10        Constrained at        0.5 Check
PLAT300_ALERT_4_G Atom Site Occupancy of F11        Constrained at        0.5 Check
PLAT300_ALERT_4_G Atom Site Occupancy of O5         Constrained at        0.5 Check
PLAT300_ALERT_4_G Atom Site Occupancy of C35        Constrained at        0.5 Check
PLAT300_ALERT_4_G Atom Site Occupancy of H591       Constrained at        0.5 Check
PLAT300_ALERT_4_G Atom Site Occupancy of H592       Constrained at        0.5 Check
PLAT300_ALERT_4_G Atom Site Occupancy of H593       Constrained at        0.5 Check
PLAT300_ALERT_4_G Atom Site Occupancy of H594       Constrained at        0.5 Check
PLAT300_ALERT_4_G Atom Site Occupancy of O4         Constrained at        0.5 Check
PLAT300_ALERT_4_G Atom Site Occupancy of H36        Constrained at        0.5 Check
PLAT300_ALERT_4_G Atom Site Occupancy of H37        Constrained at        0.5 Check
PLAT302_ALERT_4_G Anion/Solvent/Minor-Residue Disorder (Resd  2  )        86% Note
PLAT302_ALERT_4_G Anion/Solvent/Minor-Residue Disorder (Resd  3  )        57% Note
PLAT302_ALERT_4_G Anion/Solvent/Minor-Residue Disorder (Resd  4  )       100% Note
PLAT302_ALERT_4_G Anion/Solvent/Minor-Residue Disorder (Resd  5  )       100% Note
PLAT304_ALERT_4_G Non-Integer Number of Atoms in ..... (Resd  5  )       1.50 Check
PLAT432_ALERT_2_G Short Inter X...Y Contact  F7       ..C23      .       2.93 Ang.
```

```
                                            x,1/2-y,-1/2+z  =      3_554 Check
PLAT432_ALERT_2_G Short Inter X...Y Contact  O4       ..C35      .       2.99 Ang.
                                                     x,y,z  =      1_555 Check
PLAT769_ALERT_4_G CIF Embedded explicitly supplied scattering data     Please Note
PLAT793_ALERT_4_G Model has Chirality at C13         (Centro SPGR)          S Verify
PLAT794_ALERT_5_G Tentative Bond Valency for Co1       (III)      .       3.30 Info
PLAT808_ALERT_5_G No Parseable SHELXL Style Weighting Scheme Found     Please Check
PLAT860_ALERT_3_G Number of Least-Squares Restraints ............         13 Note
PLAT882_ALERT_1_G No Datum for _diffrn_reflns_av_unetI/netI ......     Please Do !
PLAT912_ALERT_4_G Missing # of FCF Reflections Above STh/L=  0.600         16 Note
PLAT929_ALERT_5_G No Weight Pars,Obs and Calc R1,wR2,S not Checked          ! Info
PLAT960_ALERT_3_G Number of Intensities with I  <   - 2*sig(I) ...         56 Check
```

```
   0 ALERT level A = Most likely a serious problem - resolve or explain
   0 ALERT level B = A potentially serious problem, consider carefully
   7 ALERT level C = Check. Ensure it is not caused by an omission or oversight
  50 ALERT level G = General information/check it is not something unexpected

   4 ALERT type 1 CIF construction/syntax error, inconsistent or missing data
   6 ALERT type 2 Indicator that the structure model may be wrong or deficient
   3 ALERT type 3 Indicator that the structure quality may be low
  39 ALERT type 4 Improvement, methodology, query or suggestion
   5 ALERT type 5 Informative message, check
```

It is advisable to attempt to resolve as many as possible of the alerts in all categories. Often the minor alerts point to easily fixed oversights, errors and omissions in your CIF or refinement strategy, so attention to these fine details can be worthwhile. In order to resolve some of the more serious problems it may be necessary to carry out additional measurements or structure refinements. However, the purpose of your study may justify the reported deviations and the more serious of these should normally be commented upon in the discussion or experimental section of a paper or in the "special_details" fields of the CIF. checkCIF was carefully designed to identify outliers and unusual parameters, but every test has its limitations and alerts that are not important in a particular case may appear. Conversely, the absence of alerts does not guarantee there are no aspects of the results needing attention. It is up to the individual to critically assess their own results and, if necessary, seek expert advice.

Publication of your CIF in IUCr journals

A basic structural check has been run on your CIF. These basic checks will be run on all CIFs submitted for publication in IUCr journals (*Acta Crystallographica*, *Journal of Applied Crystallography*, *Journal of Synchrotron Radiation*); however, if you intend to submit to *Acta Crystallographica Section C* or *E* or *IUCrData*, you should make sure that full publication checks are run on the final version of your CIF prior to submission.

Publication of your CIF in other journals

Please refer to the *Notes for Authors* of the relevant journal for any special instructions relating to CIF submission.

PLATON version of 12/09/2022; check.def file version of 09/08/2022

Datablock I - ellipsoid plot

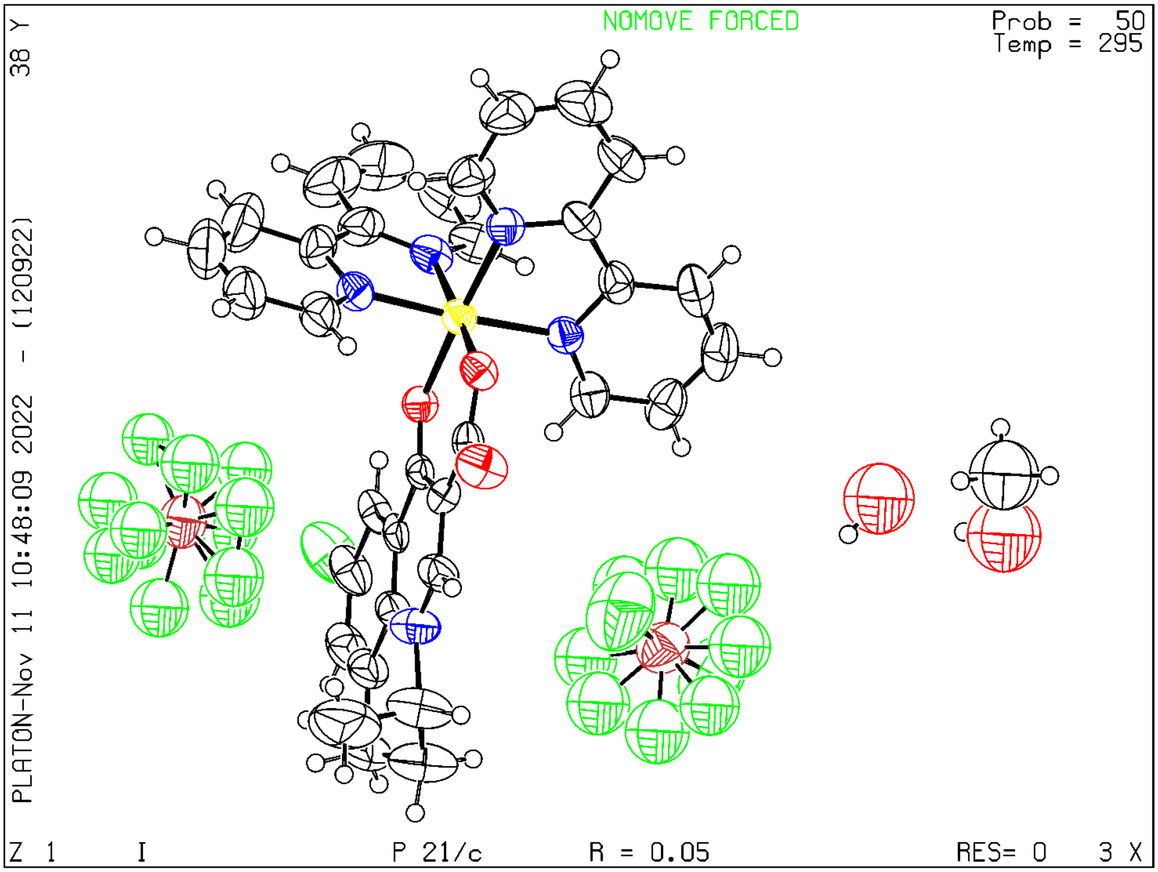

Supplement: Supplementary file 1 [file molecules-30-02646-s001.zip › Tialiou - Psomas, Checkcif of 3.pdf]
