# Supplementary material for: Synthesis, Characterization and Biological Profile of Cationic Cobalt Complexes with First-Generation Quinolones
Source: Molecules. 2025 Jun 19;30(12):2646. doi: 10.3390/molecules30122646 (PMC12196173; doi:10.3390/molecules30122646)

checkCIF/PLATON report

Structure factors have been supplied for datablock(s) I

THIS REPORT IS FOR GUIDANCE ONLY. IF USED AS PART OF A REVIEW PROCEDURE FOR PUBLICATION, IT SHOULD NOT REPLACE THE EXPERTISE OF AN EXPERIENCED CRYSTALLOGRAPHIC REFEREE.

No syntax errors found. CIF dictionary Interpreting this report

Datablock: I

Bond precision:	C-C = 0.0058 A		Wavelength=0.71073
Cell:	a=10.3630(15)	b=16.4879(19)	c=16.672(2)
	alpha=61.688(6)	beta=87.777(7)	gamma=84.807(6)
Temperature:	294 K		

	Calculated	Reported
Volume	2497.6(6)	2497.6(6)
Space group	P -1	P -1
Hall group	-P 1	?
Moiety formula	C34 H32 Co N9 O3, 2(F3 P0.50), F6 P, C H4 O, H2 O	C35 H38.00 Co1 F12 N9 O5 P2
Sum formula	C35 H38 Co F12 N9 O5 P2	C35 H38.00 Co1 F12 N9 O5 P2
Mr	1013.61	1013.60
Dx,g cm-3	1.348	1.348
Z	2	2
Mu (mm-1)	0.498	0.498
F000	1032.0	1032.0
F000'	1033.76	
h,k,lmax	12,20,20	12,20,20
Nref	9722	9554
Tmin,Tmax	0.898,0.947	0.910,0.950
Tmin'	0.896	

Correction method= # Reported T Limits: Tmin=0.910 Tmax=0.950
AbsCorr = NUMERICAL

Data completeness= 0.983 Theta(max)= 25.919

R(reflections)= 0.0567(6905) wR2(reflections)= 0.1251(6905)

S = 1.000 Npar= 617

The following ALERTS were generated. Each ALERT has the format
test-name_ALERT_alert-type_alert-level.
Click on the hyperlinks for more details of the test.

Alert level C

```
PLAT041_ALERT_1_C Calc. and Reported SumFormula    Strings  Differ     Please Check
PLAT042_ALERT_1_C Calc. and Reported MoietyFormula Strings  Differ     Please Check
PLAT260_ALERT_2_C Large Average Ueq of Residue Including       P1      0.102 Check
PLAT910_ALERT_3_C Missing # of FCF Reflection(s) Below Theta(Min).         5 Note
PLAT911_ALERT_3_C Missing FCF Refl Between Thmin & STh/L=    0.600        34 Report
```

Alert level G

```
PLAT002_ALERT_2_G Number of Distance or Angle Restraints on AtSite        24 Note
PLAT007_ALERT_5_G Number of Unrefined Donor-H Atoms .............         10 Report
PLAT093_ALERT_1_G No s.u.'s on H-positions, Refinement Reported as     mixed Check
PLAT231_ALERT_4_G Hirshfeld Test (Solvent)   P1       --F4       .       6.5 s.u.
PLAT244_ALERT_4_G Low    'Solvent' Ueq as Compared to Neighbors of        P1 Check
PLAT300_ALERT_4_G Atom Site Occupancy of N9         Constrained at       0.5 Check
PLAT300_ALERT_4_G Atom Site Occupancy of N10        Constrained at       0.5 Check
PLAT300_ALERT_4_G Atom Site Occupancy of C12        Constrained at       0.5 Check
PLAT300_ALERT_4_G Atom Site Occupancy of C13        Constrained at       0.5 Check
PLAT300_ALERT_4_G Atom Site Occupancy of C14        Constrained at       0.5 Check
PLAT300_ALERT_4_G Atom Site Occupancy of C35        Constrained at       0.5 Check
PLAT300_ALERT_4_G Atom Site Occupancy of C36        Constrained at       0.5 Check
PLAT300_ALERT_4_G Atom Site Occupancy of C37        Constrained at       0.5 Check
PLAT300_ALERT_4_G Atom Site Occupancy of H91        Constrained at       0.5 Check
PLAT300_ALERT_4_G Atom Site Occupancy of H101       Constrained at       0.5 Check
PLAT300_ALERT_4_G Atom Site Occupancy of H111       Constrained at       0.5 Check
PLAT300_ALERT_4_G Atom Site Occupancy of H112       Constrained at       0.5 Check
PLAT300_ALERT_4_G Atom Site Occupancy of H113       Constrained at       0.5 Check
PLAT300_ALERT_4_G Atom Site Occupancy of H114       Constrained at       0.5 Check
PLAT300_ALERT_4_G Atom Site Occupancy of H121       Constrained at       0.5 Check
PLAT300_ALERT_4_G Atom Site Occupancy of H122       Constrained at       0.5 Check
PLAT300_ALERT_4_G Atom Site Occupancy of H131       Constrained at       0.5 Check
PLAT300_ALERT_4_G Atom Site Occupancy of H132       Constrained at       0.5 Check
PLAT300_ALERT_4_G Atom Site Occupancy of H141       Constrained at       0.5 Check
PLAT300_ALERT_4_G Atom Site Occupancy of H142       Constrained at       0.5 Check
PLAT300_ALERT_4_G Atom Site Occupancy of H351       Constrained at       0.5 Check
PLAT300_ALERT_4_G Atom Site Occupancy of H352       Constrained at       0.5 Check
PLAT300_ALERT_4_G Atom Site Occupancy of H361       Constrained at       0.5 Check
PLAT300_ALERT_4_G Atom Site Occupancy of H362       Constrained at       0.5 Check
PLAT300_ALERT_4_G Atom Site Occupancy of H371       Constrained at       0.5 Check
PLAT300_ALERT_4_G Atom Site Occupancy of H372       Constrained at       0.5 Check
PLAT300_ALERT_4_G Atom Site Occupancy of P3         Constrained at       0.5 Check
PLAT300_ALERT_4_G Atom Site Occupancy of F17        Constrained at      0.25 Check
PLAT300_ALERT_4_G Atom Site Occupancy of F18        Constrained at      0.25 Check
PLAT300_ALERT_4_G Atom Site Occupancy of F19        Constrained at      0.25 Check
PLAT300_ALERT_4_G Atom Site Occupancy of F20        Constrained at      0.25 Check
PLAT300_ALERT_4_G Atom Site Occupancy of F21        Constrained at      0.25 Check
PLAT300_ALERT_4_G Atom Site Occupancy of F22        Constrained at      0.25 Check
PLAT300_ALERT_4_G Atom Site Occupancy of F23        Constrained at      0.25 Check
PLAT300_ALERT_4_G Atom Site Occupancy of F24        Constrained at      0.25 Check
PLAT300_ALERT_4_G Atom Site Occupancy of F25        Constrained at      0.25 Check
PLAT300_ALERT_4_G Atom Site Occupancy of F26        Constrained at      0.25 Check
```

```
PLAT300_ALERT_4_G Atom Site Occupancy of F27       Constrained at       0.25 Check
PLAT300_ALERT_4_G Atom Site Occupancy of F28       Constrained at       0.25 Check
PLAT300_ALERT_4_G Atom Site Occupancy of P2        Constrained at        0.5 Check
PLAT300_ALERT_4_G Atom Site Occupancy of F7        Constrained at        0.5 Check
PLAT300_ALERT_4_G Atom Site Occupancy of F8        Constrained at        0.5 Check
PLAT300_ALERT_4_G Atom Site Occupancy of F9        Constrained at       0.25 Check
PLAT300_ALERT_4_G Atom Site Occupancy of F10       Constrained at       0.25 Check
PLAT300_ALERT_4_G Atom Site Occupancy of F11       Constrained at       0.25 Check
PLAT300_ALERT_4_G Atom Site Occupancy of F12       Constrained at       0.25 Check
PLAT300_ALERT_4_G Atom Site Occupancy of F13       Constrained at       0.25 Check
PLAT300_ALERT_4_G Atom Site Occupancy of F14       Constrained at       0.25 Check
PLAT300_ALERT_4_G Atom Site Occupancy of F15       Constrained at       0.25 Check
PLAT300_ALERT_4_G Atom Site Occupancy of F16       Constrained at       0.25 Check
PLAT300_ALERT_4_G Atom Site Occupancy of O7        Constrained at        0.5 Check
PLAT300_ALERT_4_G Atom Site Occupancy of C38       Constrained at        0.5 Check
PLAT300_ALERT_4_G Atom Site Occupancy of H71       Constrained at        0.5 Check
PLAT300_ALERT_4_G Atom Site Occupancy of H381      Constrained at        0.5 Check
PLAT300_ALERT_4_G Atom Site Occupancy of H382      Constrained at        0.5 Check
PLAT300_ALERT_4_G Atom Site Occupancy of H383      Constrained at        0.5 Check
PLAT300_ALERT_4_G Atom Site Occupancy of O8        Constrained at        0.5 Check
PLAT300_ALERT_4_G Atom Site Occupancy of C39       Constrained at        0.5 Check
PLAT300_ALERT_4_G Atom Site Occupancy of H391      Constrained at        0.5 Check
PLAT300_ALERT_4_G Atom Site Occupancy of H392      Constrained at        0.5 Check
PLAT300_ALERT_4_G Atom Site Occupancy of H393      Constrained at        0.5 Check
PLAT300_ALERT_4_G Atom Site Occupancy of H397      Constrained at        0.5 Check
PLAT300_ALERT_4_G Atom Site Occupancy of O4        Constrained at        0.5 Check
PLAT300_ALERT_4_G Atom Site Occupancy of H41       Constrained at        0.5 Check
PLAT300_ALERT_4_G Atom Site Occupancy of H42       Constrained at        0.5 Check
PLAT300_ALERT_4_G Atom Site Occupancy of O5        Constrained at       0.25 Check
PLAT300_ALERT_4_G Atom Site Occupancy of H52       Constrained at       0.25 Check
PLAT300_ALERT_4_G Atom Site Occupancy of H396      Constrained at       0.25 Check
PLAT300_ALERT_4_G Atom Site Occupancy of O6        Constrained at       0.25 Check
PLAT300_ALERT_4_G Atom Site Occupancy of H61       Constrained at       0.25 Check
PLAT300_ALERT_4_G Atom Site Occupancy of H62       Constrained at       0.25 Check
PLAT301_ALERT_3_G Main Residue  Disorder .............(Resd  1  )          9% Note
PLAT302_ALERT_4_G Anion/Solvent/Minor-Residue Disorder (Resd  2  )        100% Note
PLAT302_ALERT_4_G Anion/Solvent/Minor-Residue Disorder (Resd  3  )        100% Note
PLAT302_ALERT_4_G Anion/Solvent/Minor-Residue Disorder (Resd  5  )        100% Note
PLAT302_ALERT_4_G Anion/Solvent/Minor-Residue Disorder (Resd  6  )        100% Note
PLAT302_ALERT_4_G Anion/Solvent/Minor-Residue Disorder (Resd  7  )        100% Note
PLAT302_ALERT_4_G Anion/Solvent/Minor-Residue Disorder (Resd  8  )        100% Note
PLAT302_ALERT_4_G Anion/Solvent/Minor-Residue Disorder (Resd  9  )        100% Note
PLAT304_ALERT_4_G Non-Integer Number of Atoms in ..... (Resd  2  )       3.50 Check
PLAT304_ALERT_4_G Non-Integer Number of Atoms in ..... (Resd  3  )       3.50 Check
PLAT304_ALERT_4_G Non-Integer Number of Atoms in ..... (Resd  7  )       1.50 Check
PLAT304_ALERT_4_G Non-Integer Number of Atoms in ..... (Resd  8  )       0.75 Check
PLAT304_ALERT_4_G Non-Integer Number of Atoms in ..... (Resd  9  )       0.75 Check
PLAT432_ALERT_2_G Short Inter X...Y Contact  F3       ..C39      .       2.85 Ang.
                                              -x,1-y,-z  =      2_565 Check
PLAT432_ALERT_2_G Short Inter X...Y Contact  O4       ..C35      .       2.76 Ang.
                                                  x,y,z  =      1_555 Check
PLAT432_ALERT_2_G Short Inter X...Y Contact  O4       ..C39      .       2.82 Ang.
                                            1-x,1-y,1-z  =      2_666 Check
PLAT432_ALERT_2_G Short Inter X...Y Contact  O8       ..C38      .       2.42 Ang.
                                                x,1+y,z  =      1_565 Check
PLAT432_ALERT_2_G Short Inter X...Y Contact  F14      ..C34      .       2.95 Ang.
                                               -1+x,y,z  =      1_455 Check
```

```
PLAT432_ALERT_2_G Short Inter X...Y Contact  O6       ..C35      .       2.78 Ang.
                                                     x,y,z  =      1_555 Check
PLAT432_ALERT_2_G Short Inter X...Y Contact  C28      ..C38      .       3.15 Ang.
                                                 1-x,-y,-z  =      2_655 Check
PLAT769_ALERT_4_G CIF Embedded explicitly supplied scattering data     Please Note
PLAT779_ALERT_4_G Suspect or Irrelevant (Bond) Angle(s) in CIF ...      22.20 Deg.
              F27  -P3   -F21     1_555   1_555   1_555 ......     #  230 Check
PLAT790_ALERT_4_G Centre of Gravity not Within Unit Cell: Resd.  #          5 Note
              C H4 O
PLAT794_ALERT_5_G Tentative Bond Valency for Co1       (III)     .       3.40 Info
PLAT808_ALERT_5_G No Parseable SHELXL Style Weighting Scheme Found     Please Check
PLAT811_ALERT_5_G No ADDSYM Analysis: Too Many Excluded Atoms ....          ! Info
PLAT860_ALERT_3_G Number of Least-Squares Restraints ............          22 Note
PLAT882_ALERT_1_G No Datum for _diffrn_reflns_av_unetI/netI ......     Please Do !
PLAT912_ALERT_4_G Missing # of FCF Reflections Above STh/L=  0.600        155 Note
PLAT929_ALERT_5_G No Weight Pars,Obs and Calc R1,wR2,S not Checked          ! Info
PLAT960_ALERT_3_G Number of Intensities with I  <   - 2*sig(I) ...        110 Check
```

---

0 **ALERT level A** = Most likely a serious problem - resolve or explain
0 **ALERT level B** = A potentially serious problem, consider carefully
5 **ALERT level C** = Check. Ensure it is not caused by an omission or oversight
107 **ALERT level G** = General information/check it is not something unexpected

4 ALERT type 1 CIF construction/syntax error, inconsistent or missing data
9 ALERT type 2 Indicator that the structure model may be wrong or deficient
5 ALERT type 3 Indicator that the structure quality may be low
89 ALERT type 4 Improvement, methodology, query or suggestion
5 ALERT type 5 Informative message, check

It is advisable to attempt to resolve as many as possible of the alerts in all categories. Often the minor alerts point to easily fixed oversights, errors and omissions in your CIF or refinement strategy, so attention to these fine details can be worthwhile. In order to resolve some of the more serious problems it may be necessary to carry out additional measurements or structure refinements. However, the purpose of your study may justify the reported deviations and the more serious of these should normally be commented upon in the discussion or experimental section of a paper or in the "special_details" fields of the CIF. checkCIF was carefully designed to identify outliers and unusual parameters, but every test has its limitations and alerts that are not important in a particular case may appear. Conversely, the absence of alerts does not guarantee there are no aspects of the results needing attention. It is up to the individual to critically assess their own results and, if necessary, seek expert advice.

Publication of your CIF in IUCr journals

A basic structural check has been run on your CIF. These basic checks will be run on all CIFs submitted for publication in IUCr journals (*Acta Crystallographica*, *Journal of Applied Crystallography*, *Journal of Synchrotron Radiation*); however, if you intend to submit to *Acta Crystallographica Section C* or *E* or *IUCrData*, you should make sure that full publication checks are run on the final version of your CIF prior to submission.

Publication of your CIF in other journals

Please refer to the *Notes for Authors* of the relevant journal for any special instructions relating to CIF submission.

PLATON version of 12/09/2022; check.def file version of 09/08/2022

Datablock I - ellipsoid plot

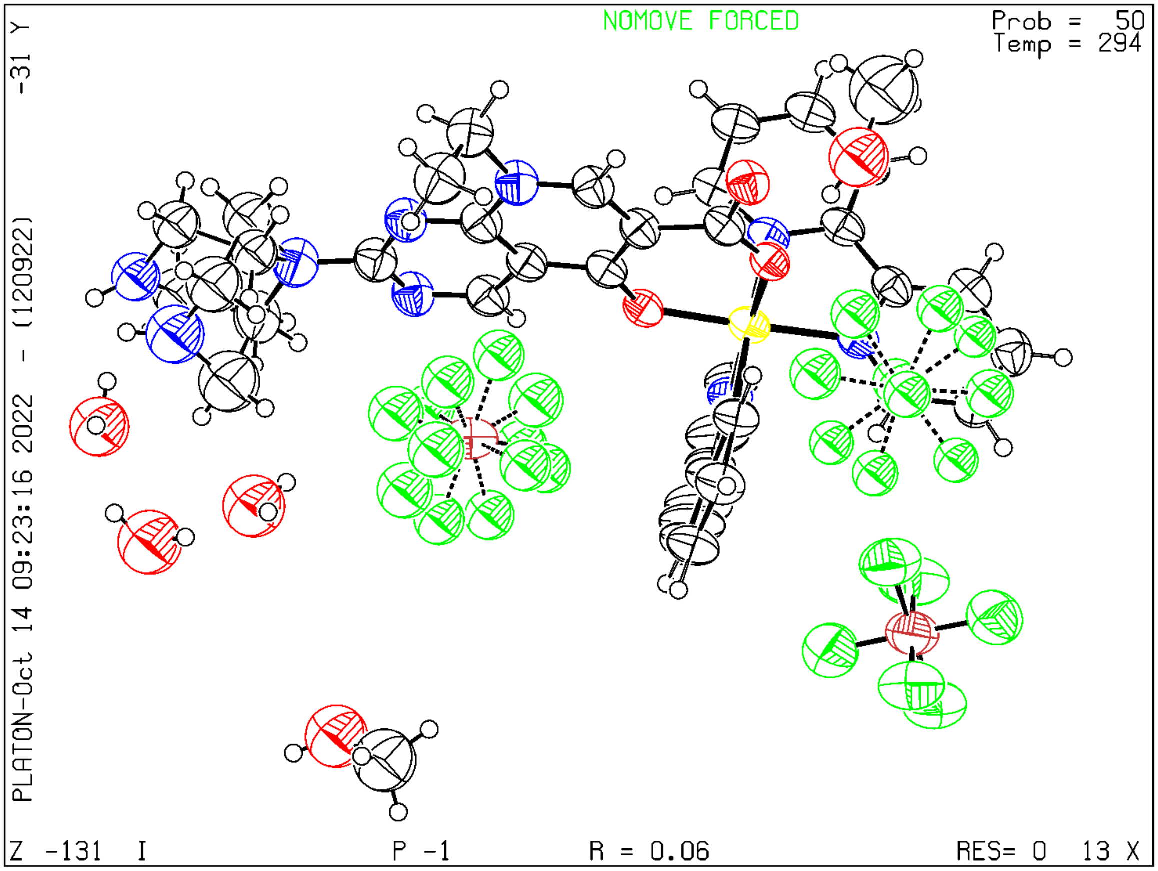

Supplement: Supplementary file 1 [file molecules-30-02646-s001.zip › Tialiou - Psomas, Checkcif of 4.pdf]
