# Supplementary material for: Synthesis, Characterization and Biological Profile of Cationic Cobalt Complexes with First-Generation Quinolones
Source: Molecules. 2025 Jun 19;30(12):2646. doi: 10.3390/molecules30122646 (PMC12196173; doi:10.3390/molecules30122646)

checkCIF/PLATON report

Structure factors have been supplied for datablock(s) I

THIS REPORT IS FOR GUIDANCE ONLY. IF USED AS PART OF A REVIEW PROCEDURE FOR PUBLICATION, IT SHOULD NOT REPLACE THE EXPERTISE OF AN EXPERIENCED CRYSTALLOGRAPHIC REFEREE.

No syntax errors found. CIF dictionary Interpreting this report

Datablock: I

Bond precision:	C-C = 0.0064 A		Wavelength=0.71073
Cell:	a=14.5285(17)	b=18.108(2)	c=15.4842(16)
	alpha=90	beta=90.389(3)	gamma=90
Temperature:	295 K		

	Calculated	Reported
Volume	4073.5(8)	4073.5(8)
Space group	P 21/a	P 21/a
Hall group	-P 2yab	?
Moiety formula	2(C36 H25 Co N6 O5), 4(F6 P), 2(C H4 O), H2 O	C36 H25 Co N6 O5, 2(F6 P), C H4 O, 0.5(H2 O)
Sum formula	C74 H60 Co2 F24 N12 O13 P4	C37 H30 Co1 F12 N6 O6.50 P2
Mr	2023.08	1011.54
Dx,g cm-3	1.649	1.649
Z	2	4
Mu (mm-1)	0.611	0.611
F000	2044.0	2044.0
F000'	2047.60	
h,k,lmax	17,22,19	17,22,18
Nref	7915	7705
Tmin,Tmax	0.896,0.918	0.910,0.920
Tmin'	0.885	

Correction method= # Reported T Limits: Tmin=0.910 Tmax=0.920
AbsCorr = NUMERICAL

Data completeness= 0.973 Theta(max)= 25.910

R(reflections)= 0.0598(5801) wR2(reflections)= 0.1177(5801)

S = 1.000 Npar= 575

The following ALERTS were generated. Each ALERT has the format
test-name_ALERT_alert-type_alert-level.
Click on the hyperlinks for more details of the test.

Alert level C

```
PLAT041_ALERT_1_C Calc. and Reported SumFormula    Strings  Differ     Please Check
PLAT042_ALERT_1_C Calc. and Reported MoietyFormula Strings  Differ     Please Check
PLAT220_ALERT_2_C NonSolvent   Resd 1  C   Ueq(max)/Ueq(min) Range        3.7 Ratio
PLAT260_ALERT_2_C Large Average Ueq of Residue Including       P1       0.134 Check
PLAT341_ALERT_3_C Low Bond Precision on  C-C Bonds ...............    0.00638 Ang.
PLAT911_ALERT_3_C Missing FCF Refl Between Thmin & STh/L=    0.600         51 Report
PLAT992_ALERT_5_C Repd & Actual _reflns_number_gt Values Differ by         25 Check
```

Alert level G

```
PLAT002_ALERT_2_G Number of Distance or Angle Restraints on AtSite         13 Note
PLAT007_ALERT_5_G Number of Unrefined Donor-H Atoms ..............          3 Report
PLAT045_ALERT_1_G Calculated and Reported Z Differ by a Factor ...      0.500 Check
PLAT128_ALERT_4_G Alternate Setting for Input Space Group  P21/a        P21/c Note
PLAT244_ALERT_4_G Low    'Solvent' Ueq as Compared to Neighbors of         P2 Check
PLAT244_ALERT_4_G Low    'Solvent' Ueq as Compared to Neighbors of         P1 Check
PLAT300_ALERT_4_G Atom Site Occupancy of F7         Constrained at        0.5 Check
PLAT300_ALERT_4_G Atom Site Occupancy of F8         Constrained at        0.5 Check
PLAT300_ALERT_4_G Atom Site Occupancy of F9         Constrained at        0.5 Check
PLAT300_ALERT_4_G Atom Site Occupancy of F10        Constrained at        0.5 Check
PLAT300_ALERT_4_G Atom Site Occupancy of F11        Constrained at        0.5 Check
PLAT300_ALERT_4_G Atom Site Occupancy of F12        Constrained at        0.5 Check
PLAT300_ALERT_4_G Atom Site Occupancy of F13        Constrained at        0.5 Check
PLAT300_ALERT_4_G Atom Site Occupancy of F14        Constrained at        0.5 Check
PLAT300_ALERT_4_G Atom Site Occupancy of F15        Constrained at        0.5 Check
PLAT300_ALERT_4_G Atom Site Occupancy of F16        Constrained at        0.5 Check
PLAT300_ALERT_4_G Atom Site Occupancy of F17        Constrained at        0.5 Check
PLAT300_ALERT_4_G Atom Site Occupancy of F18        Constrained at        0.5 Check
PLAT300_ALERT_4_G Atom Site Occupancy of O7         Constrained at        0.5 Check
PLAT300_ALERT_4_G Atom Site Occupancy of H71        Constrained at        0.5 Check
PLAT300_ALERT_4_G Atom Site Occupancy of H72        Constrained at        0.5 Check
PLAT302_ALERT_4_G Anion/Solvent/Minor-Residue Disorder (Resd  2  )        86% Note
PLAT302_ALERT_4_G Anion/Solvent/Minor-Residue Disorder (Resd  5  )       100% Note
PLAT304_ALERT_4_G Non-Integer Number of Atoms in ..... (Resd  5  )       1.50 Check
PLAT398_ALERT_2_G Deviating  C-O-C   Angle From 120 for O4       .      103.9 Degree
PLAT398_ALERT_2_G Deviating  C-O-C   Angle From 120 for O5       .      109.2 Degree
PLAT432_ALERT_2_G Short Inter X...Y Contact  F8       ..C14      .       2.92 Ang.
                                             -1/2+x,1/2-y,z  =      3_455 Check
PLAT432_ALERT_2_G Short Inter X...Y Contact  F12      ..C28      .       2.91 Ang.
                                             -1/2+x,1/2-y,z  =      3_455 Check
PLAT432_ALERT_2_G Short Inter X...Y Contact  F18      ..C13      .       2.80 Ang.
                                                      x,y,z  =      1_555 Check
PLAT769_ALERT_4_G CIF Embedded explicitly supplied scattering data     Please Note
PLAT790_ALERT_4_G Centre of Gravity not Within Unit Cell: Resd.  #          3 Note
              F6 P
PLAT794_ALERT_5_G Tentative Bond Valency for Co1       (III)     .       3.43 Info
PLAT808_ALERT_5_G No Parseable SHELXL Style Weighting Scheme Found     Please Check
PLAT860_ALERT_3_G Number of Least-Squares Restraints .............         12 Note
PLAT882_ALERT_1_G No Datum for _diffrn_reflns_av_unetI/netI ......     Please Do !
PLAT910_ALERT_3_G Missing # of FCF Reflection(s) Below Theta(Min).          3 Note
```

```
PLAT912_ALERT_4_G Missing # of FCF Reflections Above STh/L=  0.600        159 Note
PLAT929_ALERT_5_G No Weight Pars,Obs and Calc R1,wR2,S not Checked          ! Info
PLAT940_ALERT_3_G Fsqd Refinement With I > n * Sigma(I) Only .....     Please Check
PLAT960_ALERT_3_G Number of Intensities with I  <   - 2*sig(I) ...          4 Check
```

```
   0 ALERT level A = Most likely a serious problem - resolve or explain
   0 ALERT level B = A potentially serious problem, consider carefully
   7 ALERT level C = Check. Ensure it is not caused by an omission or oversight
  40 ALERT level G = General information/check it is not something unexpected

   4 ALERT type 1 CIF construction/syntax error, inconsistent or missing data
   8 ALERT type 2 Indicator that the structure model may be wrong or deficient
   6 ALERT type 3 Indicator that the structure quality may be low
  24 ALERT type 4 Improvement, methodology, query or suggestion
   5 ALERT type 5 Informative message, check
```

It is advisable to attempt to resolve as many as possible of the alerts in all categories. Often the minor alerts point to easily fixed oversights, errors and omissions in your CIF or refinement strategy, so attention to these fine details can be worthwhile. In order to resolve some of the more serious problems it may be necessary to carry out additional measurements or structure refinements. However, the purpose of your study may justify the reported deviations and the more serious of these should normally be commented upon in the discussion or experimental section of a paper or in the "special_details" fields of the CIF. checkCIF was carefully designed to identify outliers and unusual parameters, but every test has its limitations and alerts that are not important in a particular case may appear. Conversely, the absence of alerts does not guarantee there are no aspects of the results needing attention. It is up to the individual to critically assess their own results and, if necessary, seek expert advice.

Publication of your CIF in IUCr journals

A basic structural check has been run on your CIF. These basic checks will be run on all CIFs submitted for publication in IUCr journals (*Acta Crystallographica*, *Journal of Applied Crystallography*, *Journal of Synchrotron Radiation*); however, if you intend to submit to *Acta Crystallographica Section C* or *E* or *IUCrData*, you should make sure that full publication checks are run on the final version of your CIF prior to submission.

Publication of your CIF in other journals

Please refer to the *Notes for Authors* of the relevant journal for any special instructions relating to CIF submission.

PLATON version of 12/09/2022; check.def file version of 09/08/2022

Datablock I - ellipsoid plot

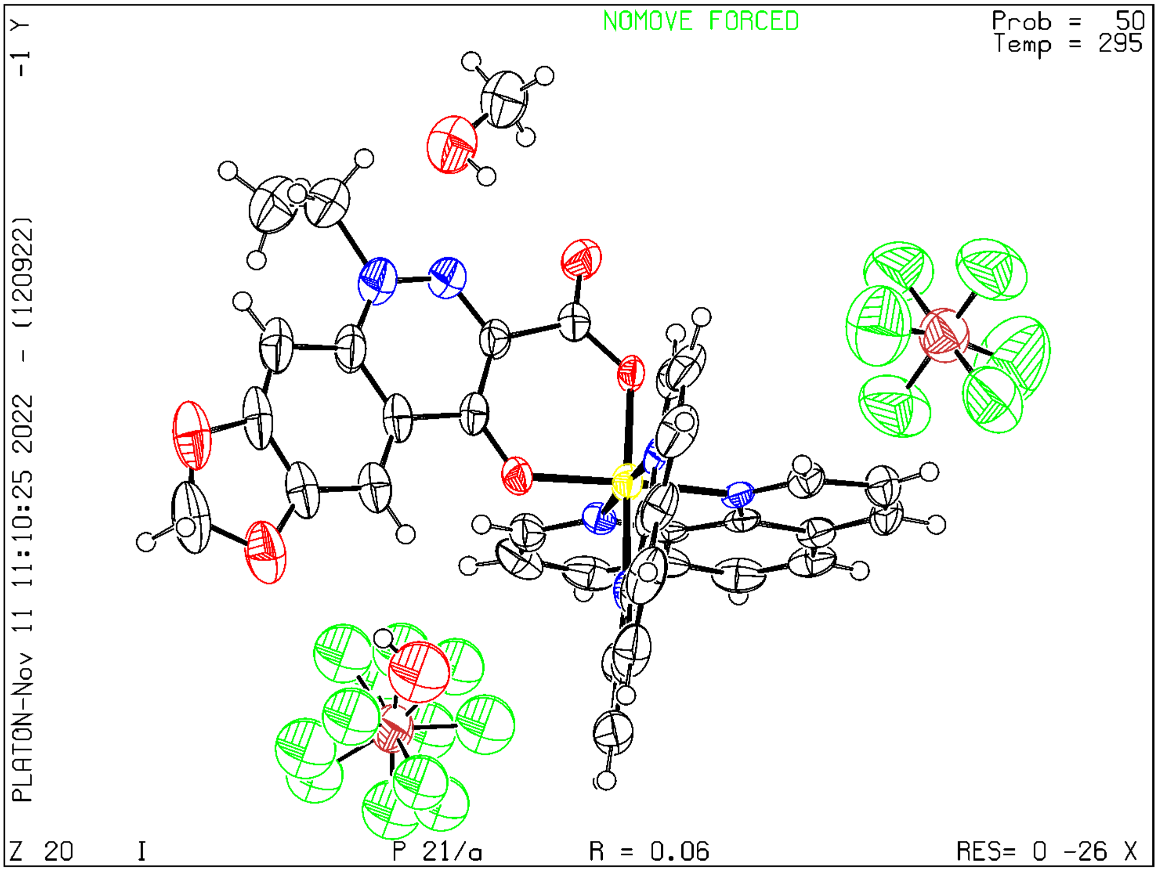

Supplement: Supplementary file 1 [file molecules-30-02646-s001.zip › Tialiou - Psomas, Checkcif of 5.pdf]
