# Supplementary material for: Synthesis, Characterization and Biological Profile of Cationic Cobalt Complexes with First-Generation Quinolones
Source: Molecules. 2025 Jun 19;30(12):2646. doi: 10.3390/molecules30122646 (PMC12196173; doi:10.3390/molecules30122646)

checkCIF/PLATON report

Structure factors have been supplied for datablock(s) I

THIS REPORT IS FOR GUIDANCE ONLY. IF USED AS PART OF A REVIEW PROCEDURE FOR PUBLICATION, IT SHOULD NOT REPLACE THE EXPERTISE OF AN EXPERIENCED CRYSTALLOGRAPHIC REFEREE.

No syntax errors found. CIF dictionary Interpreting this report

Datablock: I

Bond precision:	C-C = 0.0057 A		Wavelength=0.71073
Cell:	a=17.840(3)	b=13.639(2)	c=17.109(3)
	alpha=90	beta=105.694(4)	gamma=90
Temperature:	295 K		

	Calculated	Reported
Volume	4007.8(11)	4007.6(11)
Space group	P 21/c	P 1 21/c 1
Hall group	-P 2ybc	?
Moiety formula	2(C38 H27 Co F N5 O3), 2(F6 P), C H4 O, 2(H2 O)	C38 H27 Co F N5 O3, 2(F6 P), 0.5(C H4 O), H2 O
Sum formula	C77 H62 Co2 F14 N10 O9 P2	C38.50 H31 Co1 F7 N5 O4.50 P1
Mr	1717.17	858.59
Dx,g cm-3	1.423	1.423
Z	2	4
Mu (mm-1)	0.548	0.548
F000	1752.0	1752.0
F000'	1754.80	
h,k,lmax	21,16,21	21,16,20
Nref	7765	7598
Tmin,Tmax	0.883,0.916	0.900,0.920
Tmin'	0.877	

Correction method= # Reported T Limits: Tmin=0.900 Tmax=0.920
AbsCorr = NUMERICAL

Data completeness= 0.978 Theta(max)= 25.868

R(reflections)= 0.0548(5450) wR2(reflections)= 0.1068(5450)

S = 1.000 Npar= 508

The following ALERTS were generated. Each ALERT has the format
test-name_ALERT_alert-type_alert-level.
Click on the hyperlinks for more details of the test.

Alert level C

```
PLAT041_ALERT_1_C Calc. and Reported SumFormula    Strings  Differ     Please Check
PLAT042_ALERT_1_C Calc. and Reported MoietyFormula Strings  Differ     Please Check
PLAT260_ALERT_2_C Large Average Ueq of Residue Including        P1      0.127 Check
PLAT911_ALERT_3_C Missing FCF Refl Between Thmin & STh/L=    0.600         34 Report
PLAT918_ALERT_3_C Reflection(s) with I(obs) much Smaller I(calc) .          4 Check
```

Alert level G

```
FORMU01_ALERT_1_G  There is a discrepancy between the atom counts in the
            _chemical_formula_sum and _chemical_formula_moiety. This is
            usually due to the moiety formula being in the wrong format.
            Atom count from _chemical_formula_sum:   C38.5 H31 Co1 F7 N5 O4.5 P1
            Atom count from _chemical_formula_moiety:C38.5 H31 Co1 F13 N5 O4.5 P2
PLAT002_ALERT_2_G Number of Distance or Angle Restraints on AtSite          8 Note
PLAT007_ALERT_5_G Number of Unrefined Donor-H Atoms .............          5 Report
PLAT045_ALERT_1_G Calculated and Reported Z Differ by a Factor ...      0.500 Check
PLAT244_ALERT_4_G Low    'Solvent' Ueq as Compared to Neighbors of         P1 Check
PLAT300_ALERT_4_G Atom Site Occupancy of F4         Constrained at        0.5 Check
PLAT300_ALERT_4_G Atom Site Occupancy of F5         Constrained at        0.5 Check
PLAT300_ALERT_4_G Atom Site Occupancy of F6         Constrained at        0.5 Check
PLAT300_ALERT_4_G Atom Site Occupancy of F7         Constrained at        0.5 Check
PLAT300_ALERT_4_G Atom Site Occupancy of F8         Constrained at        0.5 Check
PLAT300_ALERT_4_G Atom Site Occupancy of F9         Constrained at        0.5 Check
PLAT300_ALERT_4_G Atom Site Occupancy of F10        Constrained at        0.5 Check
PLAT300_ALERT_4_G Atom Site Occupancy of F11        Constrained at        0.5 Check
PLAT300_ALERT_4_G Atom Site Occupancy of O6         Constrained at        0.5 Check
PLAT300_ALERT_4_G Atom Site Occupancy of C39        Constrained at        0.5 Check
PLAT300_ALERT_4_G Atom Site Occupancy of H61        Constrained at        0.5 Check
PLAT300_ALERT_4_G Atom Site Occupancy of H391       Constrained at        0.5 Check
PLAT300_ALERT_4_G Atom Site Occupancy of H392       Constrained at        0.5 Check
PLAT300_ALERT_4_G Atom Site Occupancy of H393       Constrained at        0.5 Check
PLAT300_ALERT_4_G Atom Site Occupancy of O4         Constrained at        0.5 Check
PLAT300_ALERT_4_G Atom Site Occupancy of H41        Constrained at        0.5 Check
PLAT300_ALERT_4_G Atom Site Occupancy of H42        Constrained at        0.5 Check
PLAT300_ALERT_4_G Atom Site Occupancy of O5         Constrained at        0.5 Check
PLAT300_ALERT_4_G Atom Site Occupancy of H52        Constrained at        0.5 Check
PLAT300_ALERT_4_G Atom Site Occupancy of H394       Constrained at        0.5 Check
PLAT302_ALERT_4_G Anion/Solvent/Minor-Residue Disorder (Resd  2  )        57% Note
PLAT302_ALERT_4_G Anion/Solvent/Minor-Residue Disorder (Resd  3  )       100% Note
PLAT302_ALERT_4_G Anion/Solvent/Minor-Residue Disorder (Resd  4  )       100% Note
PLAT302_ALERT_4_G Anion/Solvent/Minor-Residue Disorder (Resd  5  )       100% Note
PLAT304_ALERT_4_G Non-Integer Number of Atoms in ..... (Resd  4  )       1.50 Check
PLAT304_ALERT_4_G Non-Integer Number of Atoms in ..... (Resd  5  )       1.50 Check
PLAT769_ALERT_4_G CIF Embedded explicitly supplied scattering data     Please Note
PLAT790_ALERT_4_G Centre of Gravity not Within Unit Cell: Resd.  #          4 Note
```

```
              H2 O
PLAT793_ALERT_4_G Model has Chirality at C11         (Centro SPGR)          R Verify
PLAT794_ALERT_5_G Tentative Bond Valency for Co1       (III)     .       2.79 Info
PLAT808_ALERT_5_G No Parseable SHELXL Style Weighting Scheme Found     Please Check
PLAT860_ALERT_3_G Number of Least-Squares Restraints ............          8 Note
PLAT882_ALERT_1_G No Datum for _diffrn_reflns_av_unetI/netI ......     Please Do !
PLAT910_ALERT_3_G Missing # of FCF Reflection(s) Below Theta(Min).          2 Note
PLAT912_ALERT_4_G Missing # of FCF Reflections Above STh/L=  0.600        150 Note
PLAT929_ALERT_5_G No Weight Pars,Obs and Calc R1,wR2,S not Checked          ! Info
PLAT960_ALERT_3_G Number of Intensities with I  <   - 2*sig(I) ...        109 Check
```

```
  0 ALERT level A = Most likely a serious problem - resolve or explain
  0 ALERT level B = A potentially serious problem, consider carefully
  5 ALERT level C = Check. Ensure it is not caused by an omission or oversight
 42 ALERT level G = General information/check it is not something unexpected

  5 ALERT type 1 CIF construction/syntax error, inconsistent or missing data
  2 ALERT type 2 Indicator that the structure model may be wrong or deficient
  5 ALERT type 3 Indicator that the structure quality may be low
 31 ALERT type 4 Improvement, methodology, query or suggestion
  4 ALERT type 5 Informative message, check
```

It is advisable to attempt to resolve as many as possible of the alerts in all categories. Often the minor alerts point to easily fixed oversights, errors and omissions in your CIF or refinement strategy, so attention to these fine details can be worthwhile. In order to resolve some of the more serious problems it may be necessary to carry out additional measurements or structure refinements. However, the purpose of your study may justify the reported deviations and the more serious of these should normally be commented upon in the discussion or experimental section of a paper or in the "special_details" fields of the CIF. checkCIF was carefully designed to identify outliers and unusual parameters, but every test has its limitations and alerts that are not important in a particular case may appear. Conversely, the absence of alerts does not guarantee there are no aspects of the results needing attention. It is up to the individual to critically assess their own results and, if necessary, seek expert advice.

Publication of your CIF in IUCr journals

A basic structural check has been run on your CIF. These basic checks will be run on all CIFs submitted for publication in IUCr journals (*Acta Crystallographica*, *Journal of Applied Crystallography*, *Journal of Synchrotron Radiation*); however, if you intend to submit to *Acta Crystallographica Section C* or *E* or *IUCrData*, you should make sure that full publication checks are run on the final version of your CIF prior to submission.

Publication of your CIF in other journals

Please refer to the *Notes for Authors* of the relevant journal for any special instructions relating to CIF submission.

PLATON version of 12/09/2022; check.def file version of 09/08/2022

Datablock I - ellipsoid plot

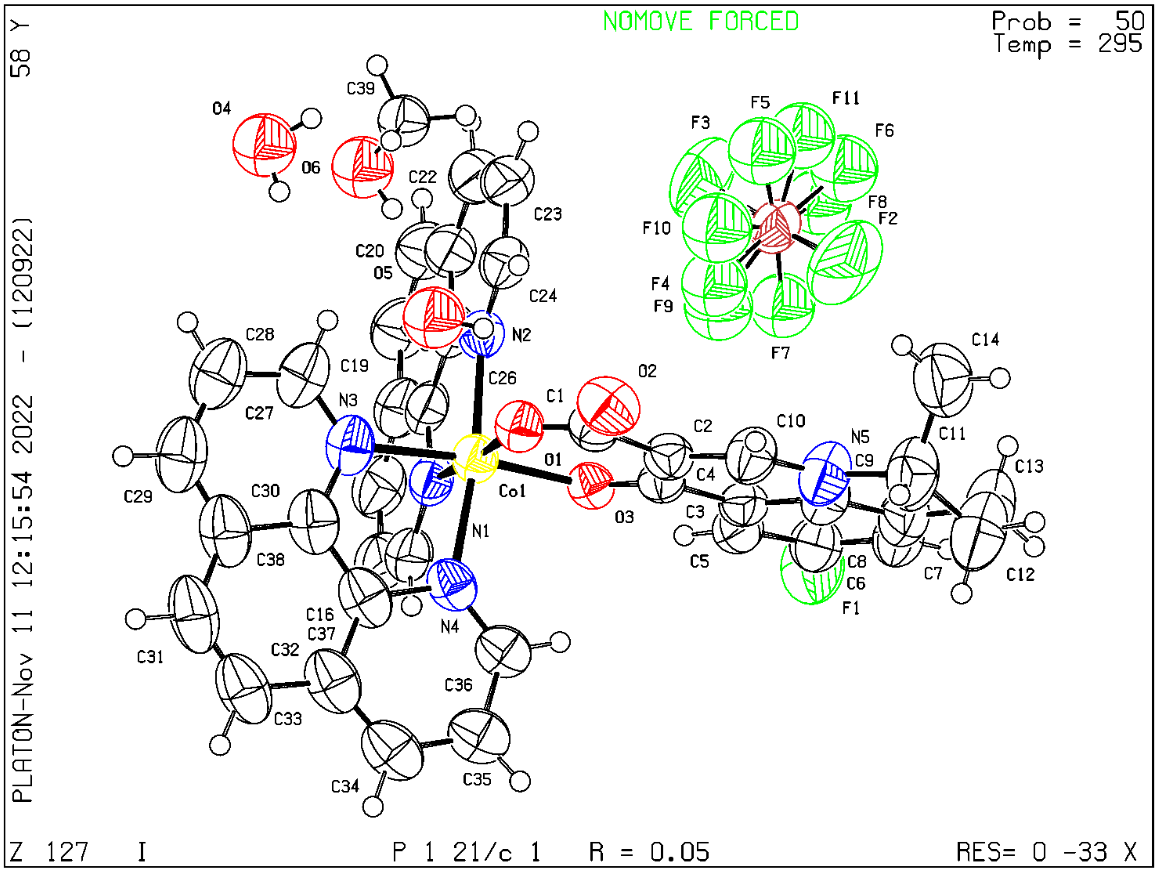

Supplement: Supplementary file 1 [file molecules-30-02646-s001.zip › Tialiou - Psomas, Checkcif of 6.pdf]
